# Gene Expression Signature of BRAF Inhibitor Resistant Melanoma Spheroids

**DOI:** 10.1007/s12253-020-00837-9

**Published:** 2020-07-01

**Authors:** Viktoria Koroknai, Vikas Patel, István Szász, Róza Ádány, Margit Balazs

**Affiliations:** 1 Public Health Research Institute, University of Debrecen, Kassai St 26/B, Debrecen, H-4028 Hungary; 2 MTA-DE Public Health Research Group, University of Debrecen, Kassai str. 26/b, Debrecen, 4028 Hungary; 3 Doctoral School of Health Sciences, University of Debrecen, Kassai St 26/B, Debrecen, H-4028 Hungary

**Keywords:** 2D and 3D cell culture, Spheroids, Gene expression and BRAF inhibitor resistance, Melanoma cell lines

## Abstract

In vitro cell cultures are frequently used to define the molecular background of drug resistance. The majority of currently available data have been obtained from 2D in vitro cultures, however, 3D cell culture systems (spheroids) are more likely to behave similarly to in vivo conditions. Our major aim was to compare the gene expression signature of 2D and 3D cultured BRAFV600E mutant melanoma cell lines. We successfully developed BRAF-drug resistant cell lines from paired primary/metastatic melanoma cell lines in both 2D and 3D in vitro cultures. Using Affymetrix Human Gene 1.0 ST arrays, we determined the gene expression pattern of all cell lines. Our analysis revealed 1049 genes (562 upregulated and 487 downregulated) that were differentially expressed between drug-sensitive cells grown under different cell cultures. Pathway analysis showed that the differently expressed genes were mainly associated with the cell cycle, p53, and other cancer-related pathways. The number of upregulated genes (72 genes) was remarkably fewer when comparing the resistant adherent cells to cells that grow in 3D, and were associated with cell adhesion molecules and IGF1R signalling. Only 1% of the upregulated and 5.6% of the downregulated genes were commonly altered between the sensitive and the resistant spheroids. Interestingly, we found several genes (*BNIP3, RING*1 and *ABHD4*) with inverse expression signature between sensitive and resistant spheroids, which are involved in anoikis resistance and cell cycle regulation. In summary, our study highlights gene expression alterations that might help to understand the development of acquired resistance in melanoma cells in tumour tissue.

**Electronic supplementary material:**

The online version of this article (10.1007/s12253-020-00837-9) contains supplementary material, which is available to authorized users.

## Introduction

Melanoma is the most serious type of skin cancer, which develops from pigment-producing cells known as melanocytes [1]. Approximately 40–60% of melanomas harbour an activating mutation in the BRAF oncogene. The most common mutation is a substitution of valine to glutamic acid (V600E) that became one of the most successful therapeutic targets of metastatic melanomas [2]. Pre-clinical and clinical studies show that targeting BRAF using RAF-selective inhibitors results in remarkable tumour shrinkage in BRAFV600E mutant melanomas; however, many of the treated patients exhibit therapy resistance due to the highly heterogeneous tumour profile [3]. Molecular mechanisms associated with BRAF inhibitor (BRAFi) resistance have shown that signal transduction pathways, such as the IGF1R/PI3K/AKT and MAPK are over-activated when the RAS/RAF/MAPK/ERK pathway is blocked by BRAFi. Combined treatment targeting the MAPK and PI3K pathways is a promising strategy to overcome BRAFi resistance in BARF mutated tumours. Combining MAPK and PI3K signaling pathway inhibitors is effective treatment of advanced stage and metastatic melanoma [4]. Studies have highlighted that overexpression of different proteins including EGFR, CRAF, N-RAS, cyclin D1 and FGF Receptor 3 also contribute to BRAFi resistance [5]. It was just recently described that long-term vemurafenib treatment in BRAF-mutant melanoma cells can lead to increased migration in association with elevated EGFR expression [6]. Molnar et al. found that high levels of EGFR are associated with a lower sensitivity against BRAF- and EGFR inhibitors and cells with high EGFR expression show significantly lower sensitivity to vemurafenib treatment and represents higher Erk activation [6]. By using preclinical model, it was proven that EGFR inhibition enhanced the antitumor effect of vemurafenib in BRAF-mutant human melanoma [7]. Beside different genetic alterations, BRAF and MITF amplifications as well as PTEN loss are also responsible for resistance to targeted therapies [8]. Amplification of MITF was found in BRAF/MEK inhibitor resistant tumours [9], which is probably associated with growth advantage when the MAPK pathway is inhibited [10].

Cell culture is a widely used in vitro tool to gain insight into cellular events [11]. The traditionally used 2D cell cultures have several limitations if we compare them to the 3D tumour tissues, including differences in cellular communication, cell morphology, cell and extracellular medium interactions that are responsible for differentiation, proliferation, gene and protein expression, responsiveness to stimuli, drug metabolism and other cellular functions. Therefore, the 2D cell culture conditions do not accurately reflect the natural structure of tissue [12]. Several studies have also suggested that adherent cell culturing changes the gene expression pattern due to unlimited access to oxygen, nutrients, metabolites and signalling molecules [13, 14].

3D spheroid culture is an improved cellular model that offers more contact space for mechanical inputs for cell adhesion, accurate atmosphere for cell migration, differentiation, survival, and growth, variable access to oxygen, nutrients, metabolites, and signalling molecules [15]. The presence of different cell types, including proliferative and necrotic cell populations, reflects the heterogeneity of tumour tissues [16]. Spheroid cell culture also reflects growth kinetics, metabolic activity and resistance to radiotherapy and chemotherapy that is more similar to tumour cells in vivo [17]. Extended cell to cell, cell to extracellular fluid interaction also alters gene expression patterns that play a crucial role in proliferation, angiogenesis, migration, invasion and drug response due to limited access to nutrients, oxygen, and waste products into and out of the compact spheroids. This results in a more compact composition of spheroids, consisting of proliferating cells, followed by quiescent cells in the middle and necrotic cells in the centre of the spheroid [18]. Three-dimensional cultures show a reduction in proliferation and increase in Beta4 and Beta1 integrin’s that are markers for cell polarization and differentiation [19].

The gene expression pattern of 3D spheroid is more comparable to in vivo solid tumours than cells cultured in monolayer [15]. Overexpressed genes are involved in cancer progression, invasion and metastasis development in other types of cancer cells including colorectal cancer and hepatocellular carcinoma besides melanoma [20]. Moreover, upregulation of metabolic, stress-response, structural, signal transduction, and cellular transport proteins in spheroids compared to 2D cultured cells have been described before [21]. Several studies described that 3D cultured cells were more resistant against an anti-cancer drug (5-FU) than 2D cultured cells due to less drug penetration into the centre cell mass (quiescent cells) and since the drug especially targets only the outer, proliferating cells [15]. On the other hand, Tirapazamine (TPZ) was more effective in spheroids than 2D cultures, likely because TPZ is more potent in during oxygen consumption [22]. It was described that structural modifications of the architecture of tumour cell cultures result in a significant upregulation of the expression of a number of genes previously shown to play a role in melanoma progression and metastatic process [18]. Three dimensional in vitro tumour models could enhance drug manufacturer’s capability to develop more effective drugs for cancer treatment [23].

The aim of our study was to develop reproducible three-dimensional melanoma spheroid models from BRAFV600E mutant melanoma cell lines that are sensitive and resistant to a BRAF inhibitor (BRAFi). Concurrently, we aimed to compare the gene expression signature of the 2D and 3D melanoma cell lines in both sensitive and resistant model systems. We successfully generated spheroids form BRAFV600E mutant BRAFi sensitive primary WM983A and metastatic WM983B cell lines originated from the same patient. Resistant cell lines were established through long-term, high dose vemurafenib analog PLX4720 inhibitor treatment.

## Material and Methods

### Cell Culture

#### 2D Cell Culture

Melanoma cell lines (WM983A and WM983B) were obtained from the Coriell Institute for Medical Research (Camden, New Jersey, USA). Cell lines were cultured in RPMI-1640 growth medium (Lonza Group Ltd., Basel, Switzerland) and supplemented with 10% foetal bovine serum (FBS from Gibco, Carlsbad, California, USA), 2 mmol/l glutamine and 50 mg/ml penicillin and streptomycin. Cell cultures was maintained at 37 °C under 5% CO_2_ atmosphere. Both cell lines harbour the BRAFV600E mutation and are wild type for NRAS. The clinicopathological characteristics of the cell lines are summarized in Table 1.

**Table. 1:** Characteristics of human melanoma cell lines

Cell line	Sex/age (age)	Origin^a^	Growth phase^b^	Histologic type^c^	BRAF ^d^	*NRAS*^d^
WM983A	Male/54	primary	VGP	NM	V600E	wt^e^
WM983B		metastasis	–	–	V600E	wt

^a^tumor type of melanomas the cell lines were derived from, ^b^VGP: vertical growth phase, ^c^NM: nodular melanoma, ^d^BRAF and NRAS mutation status, ^e^wt: wild-type

#### Establishment of BRAF Inhibitor Resistant Cell Lines

Resistant cell lines were established as described before, by continuously increasing the concentration of Vemurafenib analogue PLX4720 with every passage for 3 months [24]. In brief, WM983A, WM983B cell lines were seeded at low densities in T25 flasks until cell confluence reached about 80%. Then, the cells were switched to medium containing 5 μM PLX4720 and cultured. The surviving cells were given medium containing PLX4720 every 3 days until they reached 80% confluence (~10 weeks). The resistant cell lines were designated as WM983A^RES^ and WM983B^RES^.

#### 3D Cell Culture

Spheroid cultures were established by seeding 1.8 × 10^4^ cells/well into Corning® Costar® Ultra-Low Attachment 6 well plates containing RPMI-1640 supplemented with 2 mmol/l glutamine and 50 mg/ml penicillin and streptomycin. After 72 h the medium was complemented with 10%, FBS and the spheroids were grown for a week in complete medium. The visible spheroids were transferred into a cell culture flask and leaved to attach (~ 6 h). After attachment, the spheroids were washed, cell debris were removed by 1XPBS. Spheroids were assigned as WM983A^SPH^, WM983B^SPH^, WM983A^RES-SPH^ and WM983B^RES-SPH^.

#### RNA Isolation and Microarray Hybridization

Total RNA were extracted by using RNeasy Mini Kit (Qiagen GmbH, Hilden, Germany) according to the manufacturer’s instructions. RNA quantity and concentration were determined by using a Nano Drop ND-1000 UV Vis spectrophotometer. Only RNA samples with 260/280 nm ratio greater than 1.8 were used for further analysis. RNA quality was determined using an Agilent 2100 Bioanalyzer (Agilent Technologies Inc., Santa Clara, California, USA). RNA integrity were evaluated by RNA Integrity Number and samples with high integrity number (RNA integrity number > 7.5) were included for Affymetrix Human Gene 1.0 microarrays (Affymetrix Inc., Santa Clara, California, USA). Labelling, hybridization, and imaging setup were performed by UD-GenoMed Medical Genomic Technologies Ltd. (Clinical Genomic Center, University of Debrecen, Debrecen, Hungary) using 500 ng of sample RNA.

#### Gene Expression Analysis

Analysis of gene expression microarray data were carried out as described previously [25]. After background correction, log2 transformation and normalization, intensity data were inserted to Bioconductor BRB-Array Tools 4.6.0 Richard Simon and Amy Peng Lam (National Cancer Institute, Bethesda, USA). After normalization, quality control, and filtering steps of the data, 9653 genes were used in further analyses. To reveal the differentially expressed genes between cells growing in 2D and 3D, paired t-tests with a random variance model were applied, considering a *P* value 0.01 or less to be statistically significant. The microarray data were deposited in the Gene Expression Omnibus (GEO) repository (http://www.ncbi.nlm.nih.gov/gds) under accession under accession numbers GSE114443 and GSE148638. The filtering and normalization were performed as previously described [25].

#### Pathway Analysis

To identify significant pathways that are associated with specific gene expression patterns, we used web-based application EnrichR (http://amp.pharm.mssm.edu/Enrichr/#). Only significantly altered signalling pathway (*p* < 0.05) were included, applying FDR < 0.05 (Benjamini Hochberg adjusted) as a cut-off and considered pathways presented with at least 5 genes.

#### Real Time Quantitative PCR

Relative expression of commonly altered genes were determined by performing real-time PCR (qRT-PCR) by using a Light Cycler 480 Real-Time PCR System (Roche Diagnostics GmbH, Mannheim, Germany). Reverse transcription of the total RNA (600 ng) was performed by using a High Capacity cDNA Archive Kit (Applied Bio-systems, Foster City, California, USA). SYBR Premix Ex Taq (Takara Holding Inc., Kyoto, Japan) were used to perform qRT-PCR. Raw PCR data were analysed using the Livak method (2-ΔΔCt) with glyceraldehyde-3-phosphate dehydrogenase (GAPDH) as an internal control gene and cultured melanocyte or pooled nevi (*n* = 8) as the calibrator sample [26]. The primer sequences are listed in Supplementary Table 1. We used these primers to validate the microarray data for the selected genes and to analyse transcript levels for genes with significant changed in different direction.

### Statistical Analysis

Statistical analyses were performed using SPSS 19.0 (SPSS Inc., Chicago, Illinois, USA). Pearson’s correlation coefficient was calculated to correlate the microarray and qPCR data. Only *P* values less than 0.05 were considered statistically significant. All included data were the average of at least three independent experiments with ± standard deviation.

## Results

### Gene Expression Profiles of BRAFi Sensitive and BRAFi Resistant Melanoma Cell Lines Cultured under 2D and in 3D Conditions

**Fig. 1:**
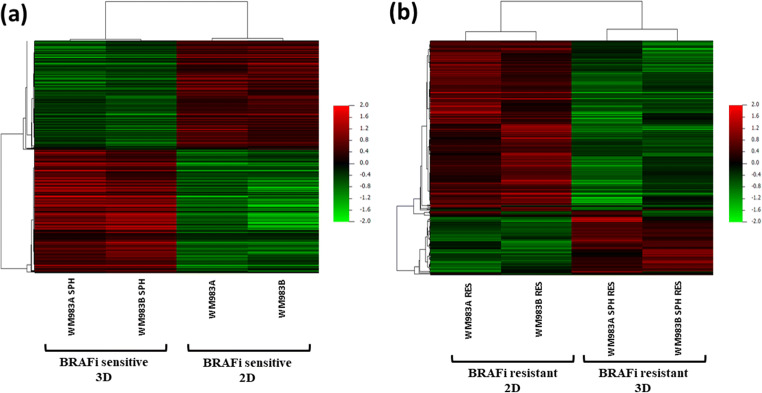
Unsupervised hierarchical clustering of genes that were differentially expressed in BRAFi sensitive and resistant melanoma cell lines cultured under 2D and 3D conditions. (**A**) Hierarchical cluster analysis was performed on 1049 genes that were differently expressed in BRAFi sensitive adherent cells (WM983A and WM983B) and spheroids (WM983A^SPH^ and WM983B^SPH^). (**B**) Hierarchical cluster analysis of 297 significantly altered genes in BRAFi resistant spheroids (WM983A^SPH-RES^ and WM983B^SPH-RES^) compared to the resistant 2D cultured cells (WM983^RES^ and WM983B^RES^). Cell lines are displayed vertically and genes displayed horizontally. The colour of each cell represents the median-adjusted expression value of each gene. Red colour indicates increased expression and green colour represents decreased expression

First, we analysed the gene expression profiles of the adherent WM983A and WM983B cell lines and their corresponding spheroids using Affymetrix Human gene 1.0 ST array. The gene expression patterns of the adherent cells were compared to the spheroids (WM98A^SPH^ and WM983B^SPH^). Figure 1. clearly shows the gene expression differences between the sensitive and resistant cell lines grown under different cell culture conditions.

Figure 1a displays the result of hierarchical clustering of 1049 genes that were differently expressed between the 2D and 3D cultured BRAFi sensitive cells. The list of genes are summarized in Supplementary Table 2. Among the differentially expressed genes, 562 were upregulated and 487 were downregulated in the spheroids compared to the adherent cell lines.

**Fig. 2:**
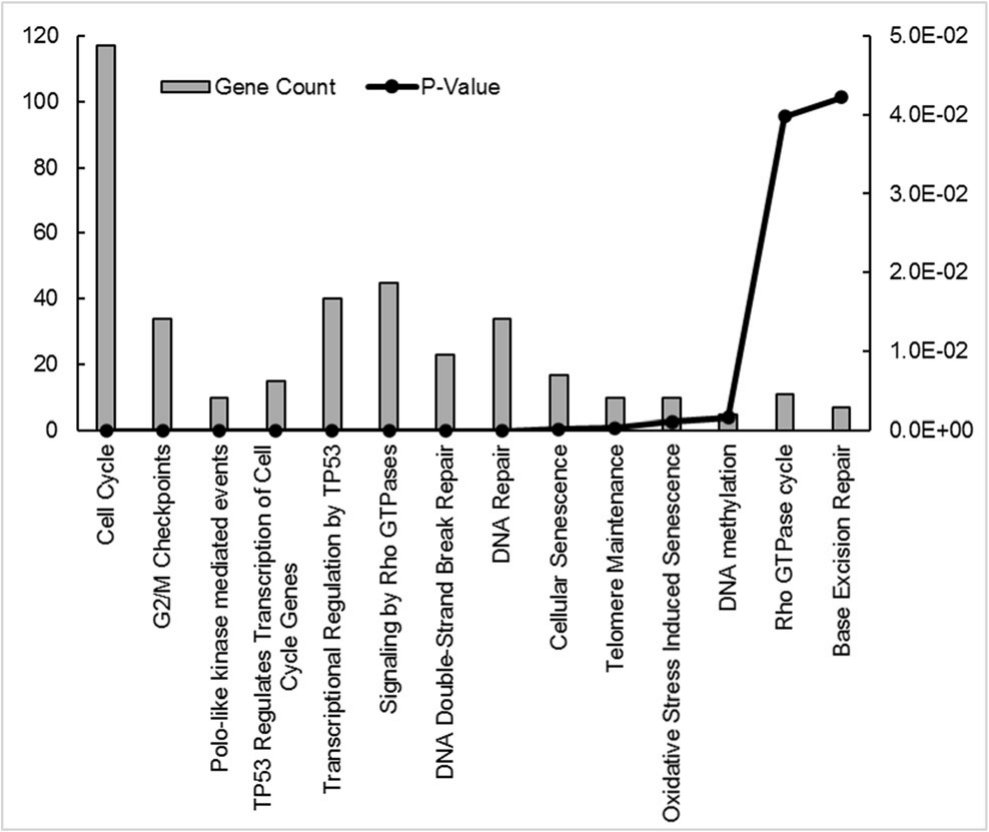
Pathway analysis of the significantly upregulated genes **(***n* = 562) in sensitive melanoma spheroid cells compared to the sensitive adherent cells. Altered molecular pathways with at least five observations are shown for the selected gene subset

**Fig. 3:**
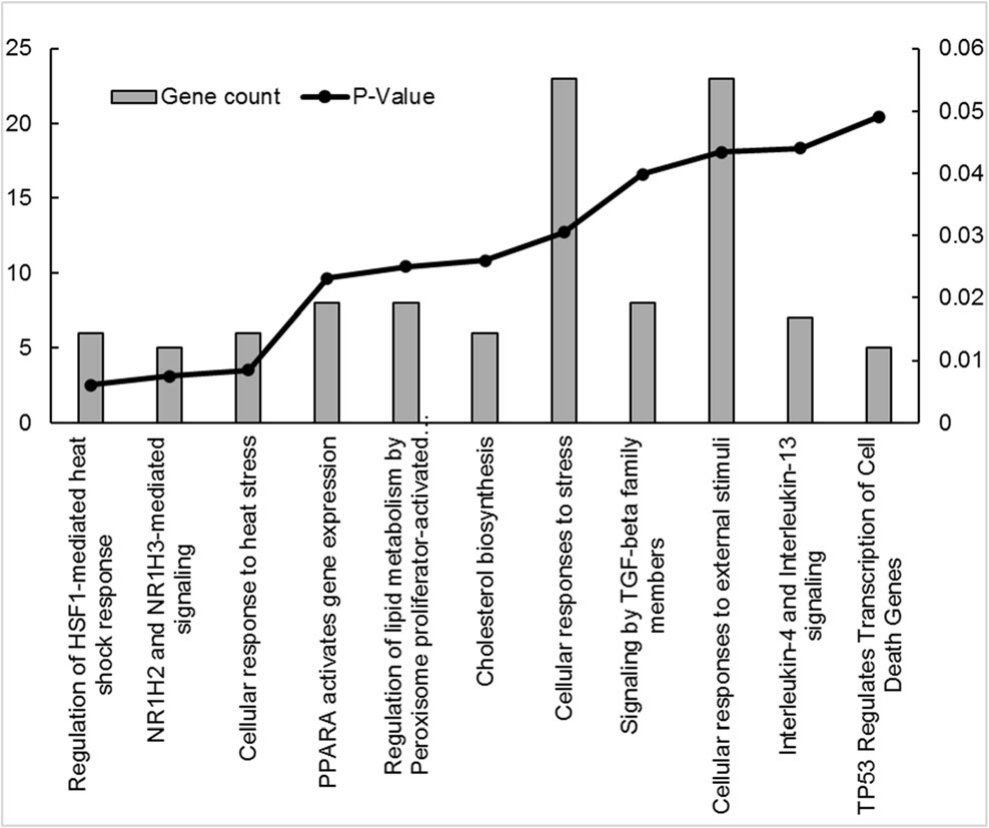
Pathway analysis of the significantly downregulated genes **(***n* = 487) in sensitive melanoma spheroid cells compared to the sensitive adherent cells. Altered molecular pathways with at least five observations are shown for the selected gene subset

In addition, we used pathway analysis to determine the functional association of all of the 1049 differently expressed genes in the BRAFi sensitive cells. A summary of the upregulated genes (*n* = 562) is listed in Supplementary Table 3. Based on the pathway analysis, upregulated genes are involved mainly in cell cycle regulation, G2/M checkpoints, DNA replication, the p53 signalling pathway, Rho GTP-ase signalling, DNA repair, and other cancer-related pathways (Fig. 2.). The list of the downregulated genes (*n* = 487) is presented in Supplementary Table 4. Downregulated genes were associated with cellular responses to external stimuli and stress, TP53 regulated transcription of cell death and other cancer related pathways (Fig. 3). The functional involvement of differently expressed genes was validated by using the Database for Annotation, Visualization and Integrated Discovery (DAVID).

**Fig. 4:**
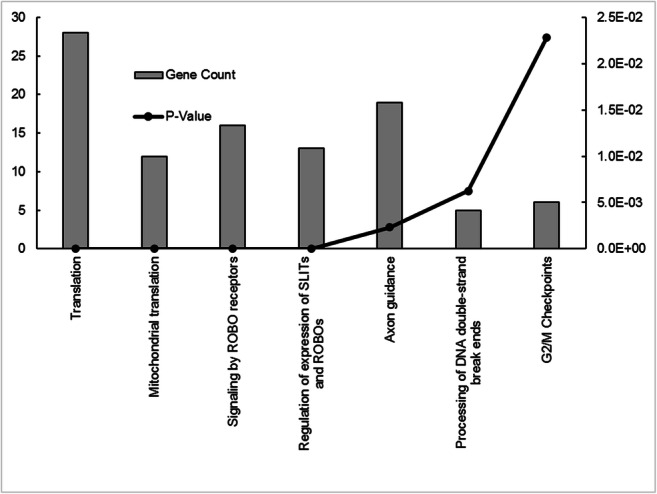
Pathway analysis of the significantly downregulated genes **(***n* = 225) in resistant melanoma spheroid cells compared to the resistant adherent cells. Altered molecular pathways with at least five observations are shown for the selected gene subset

Gene expression analysis of BRAFi resistant adherent melanoma cell lines (WM983A^RES^ and WM983B^RES^) and their corresponding spheroids (WM983A^RES-SPH^ and WM983B^RES-SPH^) revealed 297 significantly altered genes. Figure 1b shows the results of the unsupervised hierarchical cluster analysis of the resistant cell lines that were cultured under different conditions (2D and 3D). Based on the analysis 72 genes were upregulated and 225 were downregulated in the resistant spheroids compared to resistant adherent cell lines. Pathway analysis of the differentially downregulated genes (*n* = 225) revealed that the genes are mainly involved in cellular and mitochondrial translation, ROBO receptor signalling, axon guidance, G2/M checkpoints and other cancer related pathways (Fig. 4). Upregulated genes in the resistant spheroids were not significantly enriched in any pathway according to our criteria.

### Comparative Analysis of the Gene Expression Signature of the Sensitive and Resistant Cell Lines

**Fig. 5:**
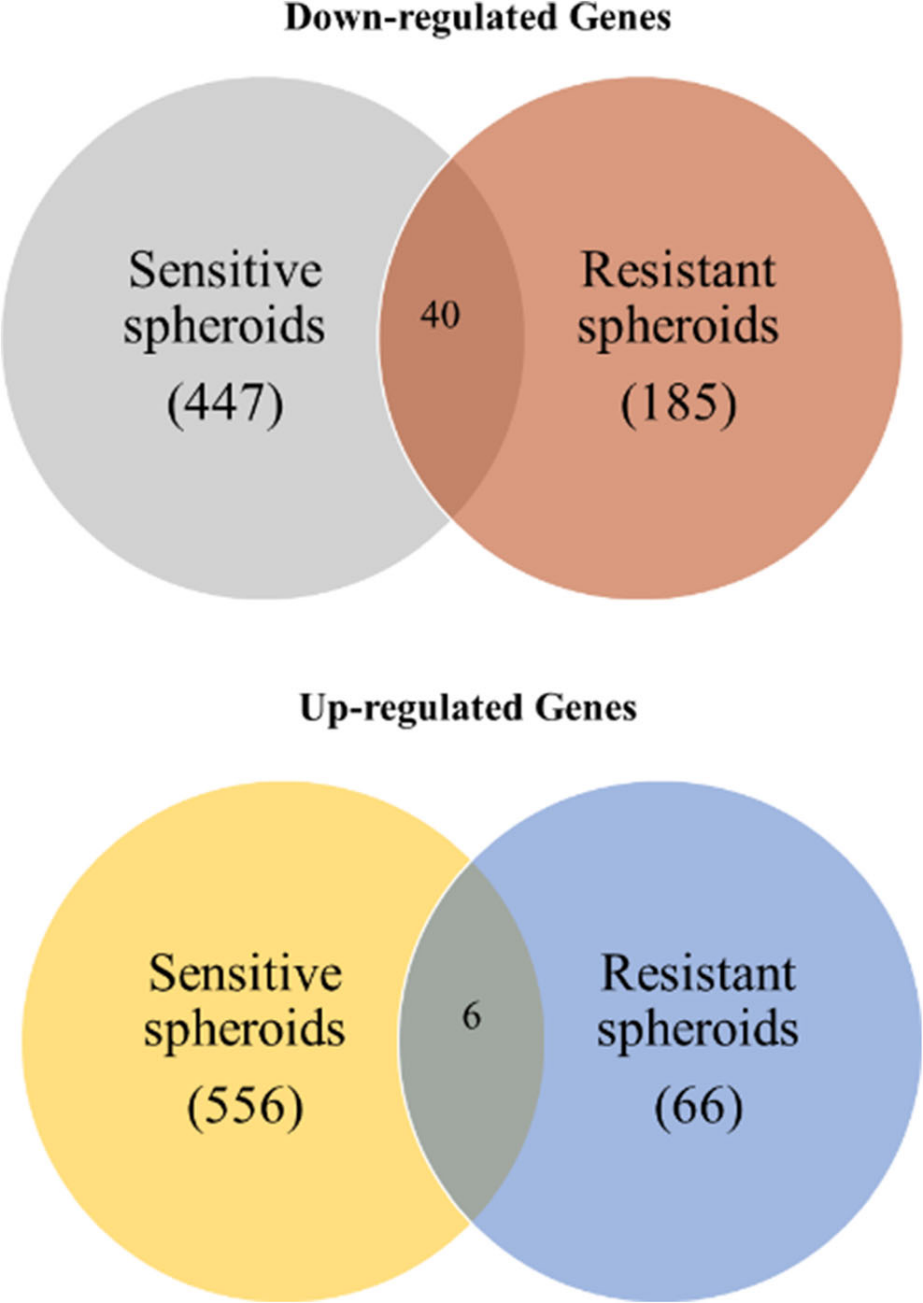
Comparison of upregulated genes in the BRAFi sensitive spheroids and resistant spheroids. Venn diagrams showing the number of genes differentially expressed by BRAFi resistant spheroids of melanoma cell lines (WM983ARES-SPH and WM983B RES-SPH, compared to respective sensitive spheroid (WM983ASPH and WM983B SPH). The diagram also shows the number of genes upregulated (6) and downregulated (40) in common between sensitive and resistant spheroid

To define the gene expression similarities and differences of the sensitive and resistant cell lines during spheroid formation, we compared the down- and upregulated genes of the WM983A and WM983B cell lines and defined the number of commonly and differently altered genes. As a result, we found a group of genes that were downregulated only in the sensitive (447 genes) and resistant (185 genes) spheroids, respectively. The number of upregulated genes in the BRAFi sensitive spheroids was 556, while 66 genes were found in the resistant spheroids (Fig. 5). Altogether 46 genes were commonly altered in both types of spheroids (Fig. 5). The list of the 46 genes are summarized in Supplementary Table 8. Most of the shared genes were downregulated (40 genes) including *MMP16*, *IGF1R*, *FLOT1* and *CEP19*. The 6 commonly upregulated genes (*HIST1H2BM, DDAH1, UCP2, MBD3L5, DEFB124* and *MLF2*) are involved in the Interleukin-2 signalling pathway and negative regulation of cell proliferation.

We also defined the main gene expression differences between the sensitive and resistant spheroids. We observed that a small portion (10 genes) of all upregulated genes (712 genes) were downregulated in the resistant spheroids, but upregulated in the sensitive spheroids (Table 2)*.* In contrast, *SCN8A*, *RING1* and *ABHD4* genes were downregulated in the sensitive spheroids and upregulated in the resistant spheroids. Some of the inversely expressed genes are involved in cell cycle (*CENPF, LOXL2, BNIP3*) and epigenetic (*HIST1H2BB*) regulation of gene expression.

**Table. 2:** Gene expression differences between sensitive and resistant spheroid formation of melanoma cells

Sl.	Gene	Fold-change	P value	Fold-change	P value
No.	symbol	in sensitive spheroids^1^		in resistant spheroid^2^	
1	*HIST1H2BB*	6.3	0.035	0.51	0.030
2	*CENPF*	4.37	0.024	0.54	0.019
2	*LOXL2*	4.36	0.028	0.62	0.046
4	*BNIP3*	1.91	0.032	0.32	0.007
5	*DCUN1D1*	1.89	0.019	0.47	0.025
6	*CMSS1*	1.68	0.018	0.59	0.034
7	*SMC3*	1.64	0.007	0.62	0.042
8	*ZNF639*	1.62	0.023	0.58	0.030
9	*IKBIP*	1.49	0.046	0.52	0.048
10	*IFT57*	1.4	0.029	0.61	0.043
11	*SCN8A*	0.63	0.001	2.66	0.012
12	*RING1*	0.45	0.032	2.17	0.029
13	*ABHD4*	0.45	0.044	1.89	0.037

^1^Comparison of gene expression between sensitive spheroids and sensitive monolayer cultures

^2^Comparison of gene expression between resistant spheroids and resistant monolayer cultures

### Validation of Microarray Data

We performed qRT-PCR to confirm the gene expression alterations of eight genes (*ABHD4, HIST1H2BB, SCN8A, CMSS1, DCUN1D1, IKBIP, SMC3* and *ZNF639).* Supplementary Table 7. summarizes the qRT-PCR data for all the sensitive and resistant cell lines. We compared the fold change levels that were obtained from Affymetrix microarray analysis with the qRT-PCR results, and found that five out of the eight genes tested (*DCUN1D1, CMSS1, ZNF639, ABHD4, and HIST1H2BB*) showed the same direction of gene expression in the sensitive- and in the resistant spheroids. In addition, strong correlation was observed between the Affymetrix array and qRT-PCR data in case of the *ABHD4* and *SCN8A* genes (R > 0.7; *P* value <0.05).

## Discussion

Three dimensional cell culturing, especially multicellular spheroids, has been gaining interest in molecular biology in the past decades to mimic the structure of tumour tissues more effectively than 2D monolayer cell cultures [11, 12]. Several methods can be applied to generate multicellular spheroids; however, extensive manipulation leads to alterations in the original features [27]. Development of 3D models for in vitro anti-cancer drug testing can give new insights into cancer cell behaviour [28]. Recently pharmaceutical companies are highly interested in new in vitro cellular models to test potential drugs [29]. The relevance of using 3D-, in addition to monolayer cell cultures was evaluated for breast cancer drug sensitivity and resistance by Breslin et al., and they concluded that the biological information represented by 3D and 2D cell cultures is substantially different [30]. They described that 3D cell cultures demonstrate higher innate resistance to anti-cancer drugs compared to the adherent cell cultures. It was just recently reported by Ryabaya et al. [31] that binimetinib (MEK inhibitor) combined with metformin is a promising therapy against melanoma and described that this combination of drugs has a synergistic effect on melanoma cells. In addition, the combined treatment provides pronounced spheroid disruption in comparison with either drug alone. Several studies have reported specific, differently expressed genes involved in BRAF inhibitor resistance in melanoma cell lines, however most of these studies are based on monolayer cell cultures [32]. However, very few data are available about the gene expression differences between melanoma cells cultured as monolayer (2D) and spheroids (3D) [14]. No data were published yet about the gene expression differences between drug sensitive and resistant melanoma cells growing under different cell culture (2D and 3D) conditions.

Our study is the first to investigate the gene expression differences between BRAFi (PLX4720, a vemurafenib analogue) sensitive and resistant melanoma cell lines growing under adherent- and three dimensional (spheroid) cell culture conditions. After long term treatment, we successfully developed BRAFi sensitive and resistant spheroids from primary tumour (WM983A) and metastasis (WM983B) originated cell lines, and compared the gene expression patterns to the corresponding monolayer cell lines. Based on our data we found that the gene expression signature of the BRAFi sensitive and resistant spheroids are highly different compared to the gene expression of the sensitive adherent cells. The number of differently expressed genes were 1049 (562 upregulated and 487 downregulated). Among the top 10-upregulated genes in the sensitive 3D cultured cells, we found *SPC25**,* *CCL2**, CCNE2* and *PLK1*. These genes have been identified previously to have functional roles in cell migration and metastasis formation and are all involved in cell cycle regulation through different pathways [33–35]. The top 10 downregulated genes are involved in different signalling pathways, such as the *CHN2* gene in the regulation of RAC1 activity, the *FOS* gene in EGFR signalling and *ITGA7* in integrin pathways and Akt signalling and in tumour initiation and progression [36–38].

When comparing the gene expression of BRAFi resistant spheroid cells to those growing in monolayer we found 297 differentially expressed (225 downregulated and 72 upregulated) genes. Pathway analysis of the downregulated genes showed that these genes are mainly involved in different translation pathways, ROBO receptor signalling, axon guidance, G2/M checkpoints and other cancer related pathways. Argast et al. has published previously that axon guidance genes are repressed by oncogenic B-Raf/MKK/ERK signalling in melanoma [39]. Genes of this signalling pathway were down-regulated in our resistant spheroids as well, including plexin B1 and semaphorin 3D genes, as well as R-RAS, known to mediate plexin-semaphorin signaling [39]. On the other hand a significant loss of the ROBO receptors was published in melanoma by Denk et al., and it is important to note that these receptors are best known for mediating axon guidance through attraction or repulsion of growth cones [40].

Differential expression analysis revealed a sets of genes that were differentially expressed in the resistant and sensitive spheroids. A group of genes were down- or upregulated only in the sensitive and resistant spheroids, respectively. Altogether 46 genes were commonly altered in both type of spheroids. Most of the shared genes (40 genes) were downregulated including *MMP16*, *IGF1R*, *FLOT1* and *CEP19* and are functionally involved in several types of cancers, including melanoma [39–41]. The 6 commonly upregulated genes (*HIST1H2BM, DDAH1, UCP2, MBD3L5, DEFB124* and *MLF2*) have roles in interleukin-2 signalling pathway and negative regulation of cell proliferation.

We also defined the main gene expression differences between the sensitive and resistant spheroids. We observed that a small portion (10 genes) of all the upregulated genes (712 genes) were downregulated in the resistant spheroids, but upregulated in the sensitive spheroids (Table 2)*.* In contrast, *SCN8A*, *RING1* and *ABHD4* genes were downregulated in the sensitive spheroids and upregulated in the resistant spheroids. Some of the inversely expressed genes are involved in the cell cycle (*CENPF, LOXL2, BNIP3*) and epigenetic (*HIST1H2BB*) regulation of gene expression. Dave et al. has described that myeloid leukemia factor 2 (MLF2) plays important an role in tumour initiation and metastasis in breast cancer [41]. Changes in expression of uncoupling protein 2 (UCP2) are tightly related to changes in cell proliferation, and that it plays a vital role in molecular events associated with carcinogenesis. Using either a genetic or pharmacological approach, induction of UCP2 sensitizes melanomas to programmed cell death protein-1 blockade treatment and elicits effective anti-tumour responses [42]. Based on the published data approximately 80% of melanoma cell lines show overexpressed DDAH-1 that represent a potential target for control of nitric oxide production in melanoma cell line [43]. On the other hand suppression of S-phase histone HIST1H2BB can improve treatment outcome in melanoma cells [44]. In contrast, genes that were commonly downregulated in sensitive and resistant melanoma spheroids, including *MMP16*, *IGF1R* and *FLOT1*, are associated with malignancy, and several studies have reported that these genes are related to the aggressive behaviour of melanoma [45, 46]. We assume that the commonly altered genes in the sensitive and resistant spheroids are essential in the formation of melanoma spheroids. In addition, beside the commonly expressed genes, our comparative study revealed differently altered genes as well. Alteration of the *SCN8A* gene (encoding type VIII alpha subunit of voltage gated sodium channel), which was down-regulated in the sensitive- and upregulated in the resistant spheroids, was described for several tumour types and it has been found that *SCN8A* gene expression level is significantly lower in tumour tissues compared to paired normal tissues [47]. Similarly, ring finger protein-1 (RING1) was also found as a differently expressed gene, which is involved in epigenetic regulation in cancer [48], where it acts as a transcriptional repressor and plays an important role in the development of aggressive phenotypes in melanoma [49]. Furthermore, upregulation of the ABHD4 gene was also common in resistant spheroids compared to sensitive ones. This gene was identified as a potential regulator of anoikis sensitivity that is one of primary events of tumour metastasis [50], highlighting that the resistant cell lines have an aggressive phenotype. Anoikis is a specific type of cell death (an endogenous death program), in which normal cells undergo apoptosis after they disconnect from the surrounding tissue cells and their extracellular matrix (ECM) [50]. Anoikis resistance is one of the hallmarks of cancer that enables tumour cells to survive in foreign environment to promote metastatic potential [51]. Interestingly, this mechanism is seems to be upregulated specifically in resistant spheroids, however, this observation requires further investigation.

On the other hand, a small cohort of genes (*HIST1H2BB, CENPF, LOXL2, BNIP3, DCUN1D1, CMSS1, SMC3, ZNF639, IKBIP* and *IFT57*) was upregulated in the sensitive spheroids, and downregulated in the resistant 3D cultures. Several of these genes are associated with tumour initiation and progression but not well documented at the field of melanoma, however, according to the results of this present study, they might have roles in BRAFi resistance. cBioPortal for Cancer Genomics database supports the idea that seventeen canonical histone H2B genes including HIST1H2B, HIST1H2BM has been found to be involved in cancer progression [44], however no direct role of the genes are described yet. Kim et al. reported that CENPF is functionally involved in the tumorigenesis of human cancers and cancer driver genes [52]. Lysyl oxidase-like 2 (LOXL2) plays a part in epithelial-mesenchymal transition by stabilizing the transcription factor SNAI1 and works as a tumour promoters in human melanoma cells by enhancing their invasive potential and several tumorigenic events including evasion of apoptosis, cell proliferation and has been found to be overexpressed in primary and metastatic melanoma and other human cancers, therefore it might have important role in vivo in the development of resistance [53]. Overexpression of hypoxia responsive protein BNIP3 in cancerous cells is highly controversial as it has been reported to be associated with promoting cell death, and it has tumour suppresser activity, however, it could also enhance aggressive behaviour of tumours such as cell migration [54, 55]. Ubiquitin-like ligase DCUN1D1 is involved in the malignant transformation of squamous cell lineage and has been identified as a potential cancer driver gene [56]. SMC3 gene expression has been reported in many cancers including acute myeloid leukaemia, bladder, and colorectal cancer [57]. The zinc-finger protein ZNF639 has been identified to be overexpressed in oesophageal squamous cell carcinomas [58]. Resisting cell death is one of the hallmarks of cancer that is associated with the IFT57 gene [59].

Development of acquired resistance after initial response leads to tumour regrowth [24]. Both genetic and epigenetic alterations are involved in the development of acquired resistance [2]. The identification of the molecular background of acquired resistance has been one of the major focuses of melanoma research in the past few years. Our study highlights important gene expression alterations that might help to understand the development of acquired resistance in melanoma cells.

### Summary

Taken together, our data provide the first insight on differently expressed genes that might be involved in 3D spheroid formation in BRAFi sensitive and resistant melanoma cells. Generally, these results underline the molecular background of spheroid formation and highlight important molecular pathways that are different between 2D and 3D cell culture. We provide large-scale gene expression analysis data between the traditional 2D and 3D melanoma cell culture, as well as detailed analysis to clearly show gene expression differences between BRAFi sensitive- and resistant melanoma spheroids. The data presented here clearly shows the major differences of gene expressions between the traditional and 3D cell culture and these data might be useful to better understand the resistance profile of melanoma cells in tumour tissue. Although remarkable achievements have been made during the last decades, there a lot of questions that remain to be answered. Further studies and consistent results are needed to identify the responsible key pathway(s) associated with drug resistance in melanoma.

## Electronic supplementary material

ESM 1 (DOCX 15 kb)

ESM 2 (DOCX 128 kb)

ESM 3 (DOCX 29 kb)

ESM 4 (DOCX 14 kb)

ESM 5 (DOCX 42 kb)

ESM 6 (DOCX 17 kb)

ESM 7 (DOCX 17 kb)

ESM 8 (DOCX 13 kb)
