# Supplementary material for: Gene Expression Signature of BRAF Inhibitor Resistant Melanoma Spheroids
Source: Pathol Oncol Res. 2020 Jul 1;26(4):2557–66. doi: 10.1007/s12253-020-00837-9 (PMC7471197; doi:10.1007/s12253-020-00837-9)
Supplement: Supplementary file 1 — (DOCX 15 kb) [file 12253_2020_837_MOESM1_ESM.docx]

| **Supplementary Table 1 - Primer sequences used in RT-qPCR experiments** | | | |  |
| --- | --- | --- | --- | --- |
| Gene | Primer sequence (5’-3’)^1^ | Amplicon size |  |  |
|  |  | (bp)^2^ |  |  |
| GAPDH* | F: AGCCACATCGCTCAGACAC | 66 |  |  |
|  | R: GCCCAATACGACCAAATCC |  |  |  |
| HIST1H2BB | F: ATGCCTGAACCCTCTAAGTCT | 98 |  |  |
|  | R: CTGCGCTTACGCTTCTTACCA |  |  |  |
| DCUN1D1 | F: AGGATCATTGGACAGGAAGAAGT | 102 |  |  |
|  | R: TGCCAGGTCATCACAGAACTG |  |  |  |
| CMSS1 | F: TAGCAGCAGACGCTTGGTG | 83 |  |  |
|  | R: TGTGAGTCAAATCATTGGCCTT |  |  |  |
| SMC3 | F: AACATAATGTGATTGTGGGCAGA | 244 |  |  |
|  | R: TCCTTTTTGGCACCAATAACTCT |  |  |  |
| ZNF639 | F: AAGACTCTACACCCTTCTCGTT | 163 |  |  |
|  | R: ACGTCTCGGTATCAGAATCATCA |  |  |  |
| IKBIP | F: GCTCATCTAAAGCGTCTACAGG | 102 |  |  |
|  | R: AAGCGTCGTCAGACTGTTGTT |  |  |  |
| SCN8A | F: CCTTTCACCCCTGAGTCACTG | 131 |  |  |
|  | R: AGGTCGCTGTTTGGCTTGG |  |  |  |
| ABHD4 | F: TCCCCTCCGACCAACTAACC | 109 |  |  |
|  | R: AGCTACTCGAAGAACAGCCAA |  |  |  |
| ^1^F: forward, R: reverse; ^2^bp: base pair and *House keeping gene. | | |  |  |
