# Supplementary material for: Gene Expression Signature of BRAF Inhibitor Resistant Melanoma Spheroids
Source: Pathol Oncol Res. 2020 Jul 1;26(4):2557–66. doi: 10.1007/s12253-020-00837-9 (PMC7471197; doi:10.1007/s12253-020-00837-9)
Supplement: Supplementary file 2 — (DOCX 128 kb) [file 12253_2020_837_MOESM2_ESM.docx]

**Supplementary Table 2**

List of genes which are differentially expressed in cell lines derived from sensitive melanoma spheroid comparing to sensitive monolayer cultures (N=1049). Genes are sorted by fold change (upregulated if fold change >1; downregulated if fold change < 1)

| Sl.  No. | Gene  symbol | Description | Fold  change | Alteration |
| --- | --- | --- | --- | --- |
| 1 | SPC25 | SPC25 component of NDC80 kinetochore complex | 20.346 | Upregulated |
| 2 | CCL2 | C-C motif chemokine ligand 2 | 19.816 | Upregulated |
| 3 | APOBEC3B | apolipoprotein B mRNA editing enzyme catalytic subunit 3B | 18.610 | Upregulated |
| 4 | CCNE2 | cyclin E2 | 17.263 | Upregulated |
| 5 | NCAPG | non-SMC condensin I complex subunit G | 16.254 | Upregulated |
| 6 | MKI67 | marker of proliferation Ki-67 | 15.862 | Upregulated |
| 7 | KIF20A | kinesin family member 20A | 15.115 | Upregulated |
| 8 | DLGAP5 | DLG associated protein 5 | 15.032 | Upregulated |
| 9 | PLK1 | polo like kinase 1 | 14.690 | Upregulated |
| 10 | CLSPN |  | 13.854 | Upregulated |
| 11 | HIST1H2BM |  | 12.910 | Upregulated |
| 12 | RRM2 | ribonucleotide reductase regulatory subunit M2 | 12.412 | Upregulated |
| 13 | ESCO2 | establishment of sister chromatid cohesion N-acetyltransferase 2 | 12.060 | Upregulated |
| 14 | CEP55 | centrosomal protein 55 | 11.867 | Upregulated |
| 15 | CCNA2 | cyclin A2 | 11.788 | Upregulated |
| 16 | CDC45 | cell division cycle 45 | 11.708 | Upregulated |
| 17 | MYBL2 | MYB proto-oncogene like 2 | 11.438 | Upregulated |
| 18 | TYMS | thymidylate synthetase | 11.240 | Upregulated |
| 19 | NUSAP1 | nucleolar and spindle associated protein 1 | 11.221 | Upregulated |
| 20 | SHCBP1 | SHC binding and spindle associated 1 | 11.010 | Upregulated |
| 21 | FAM111B | family with sequence similarity 111 member B | 10.708 | Upregulated |
| 22 | SGOL1 |  | 10.372 | Upregulated |
| 23 | IL7R | interleukin 7 receptor | 10.070 | Upregulated |
| 24 | NUF2 | NUF2 component of NDC80 kinetochore complex | 9.895 | Upregulated |
| 25 | TXNIP | thioredoxin interacting protein | 9.640 | Upregulated |
| 26 | DEPDC1 | DEP domain containing 1 | 9.607 | Upregulated |
| 27 | CDK1 | cyclin dependent kinase 1 | 9.377 | Upregulated |
| 28 | SKA1 | spindle and kinetochore associated complex subunit 1 | 8.960 | Upregulated |
| 29 | DTL | denticleless E3 ubiquitin protein ligase homolog | 8.693 | Upregulated |
| 30 | ASPM | abnormal spindle microtubule assembly | 8.665 | Upregulated |
| 31 | KIF11 | kinesin family member 11 | 8.665 | Upregulated |
| 32 | CXCL8 | C-X-C motif chemokine ligand 8 | 8.646 | Upregulated |
| 33 | NDC80 | NDC80 kinetochore complex component | 8.642 | Upregulated |
| 34 | CKAP2L | cytoskeleton associated protein 2 like | 8.594 | Upregulated |
| 35 | CCNB2 | cyclin B2 | 8.371 | Upregulated |
| 36 | TK1 | thymidine kinase 1 | 8.370 | Upregulated |
| 37 | C12orf75 | chromosome 12 open reading frame 75 | 8.330 | Upregulated |
| 38 | SKA3 | spindle and kinetochore associated complex subunit 3 | 8.160 | Upregulated |
| 39 | PLK4 | polo like kinase 4 | 7.899 | Upregulated |
| 40 | HJURP | Holliday junction recognition protein | 7.725 | Upregulated |
| 41 | CENPU | centromere protein U | 7.680 | Upregulated |
| 42 | GINS2 | GINS complex subunit 2 | 7.656 | Upregulated |
| 43 | CDKN3 | cyclin dependent kinase inhibitor 3 | 7.455 | Upregulated |
| 44 | ITGA4 | integrin subunit alpha 4 | 7.428 | Upregulated |
| 45 | TRIP13 | thyroid hormone receptor interactor 13 | 7.355 | Upregulated |
| 46 | CENPE | centromere protein E | 7.308 | Upregulated |
| 47 | CDCA2 | cell division cycle associated 2 | 7.225 | Upregulated |
| 48 | NEK2 | NIMA related kinase 2 | 7.219 | Upregulated |
| 49 | KIF14 | kinesin family member 14 | 7.214 | Upregulated |
| 50 | CDC20 | cell division cycle 20 | 7.146 | Upregulated |
| 51 | NEIL3 | nei like DNA glycosylase 3 | 7.066 | Upregulated |
| 52 | HIST1H1A |  | 7.061 | Upregulated |
| 53 | CCNB1 | cyclin B1 | 7.027 | Upregulated |
| 54 | ANLN | anillin actin binding protein | 6.969 | Upregulated |
| 55 | BRIP1 | BRCA1 interacting protein C-terminal helicase 1 | 6.886 | Upregulated |
| 56 | DSCC1 | DNA replication and sister chromatid cohesion 1 | 6.675 | Upregulated |
| 57 | DIAPH3 | diaphanous related formin 3 | 6.641 | Upregulated |
| 58 | POLE2 | DNA polymerase epsilon 2, accessory subunit | 6.549 | Upregulated |
| 59 | KIF15 | kinesin family member 15 | 6.514 | Upregulated |
| 60 | VCAN | versican | 6.361 | Upregulated |
| 61 | NFE2L3 | nuclear factor, erythroid 2 like 3 | 6.320 | Upregulated |
| 62 | HIST1H2BB |  | 6.309 | Upregulated |
| 63 | DEPDC1B | DEP domain containing 1B | 6.290 | Upregulated |
| 64 | KIF2C | kinesin family member 2C | 6.272 | Upregulated |
| 65 | EXO1 | exonuclease 1 | 6.246 | Upregulated |
| 66 | CENPI | centromere protein I | 6.127 | Upregulated |
| 67 | CDCA8 | cell division cycle associated 8 | 6.014 | Upregulated |
| 68 | RAD51AP1 | RAD51 associated protein 1 | 5.847 | Upregulated |
| 69 | KIF23 | kinesin family member 23 | 5.839 | Upregulated |
| 70 | TMEM171 | transmembrane protein 171 | 5.796 | Upregulated |
| 71 | KIFC1 | kinesin family member C1 | 5.654 | Upregulated |
| 72 | DDIAS | DNA damage induced apoptosis suppressor | 5.603 | Upregulated |
| 73 | RAD51 | RAD51 recombinase | 5.583 | Upregulated |
| 74 | ZNF738 | zinc finger protein 738 | 5.515 | Upregulated |
| 75 | KIAA1524 |  | 5.494 | Upregulated |
| 76 | PTX3 | pentraxin 3 | 5.494 | Upregulated |
| 77 | XRCC2 | X-ray repair cross complementing 2 | 5.372 | Upregulated |
| 78 | PARPBP | PARP1 binding protein | 5.364 | Upregulated |
| 79 | UBD |  | 5.361 | Upregulated |
| 80 | NCAPH | non-SMC condensin I complex subunit H | 5.346 | Upregulated |
| 81 | MCM10 | minichromosome maintenance 10 replication initiation factor | 5.296 | Upregulated |
| 82 | MELK | maternal embryonic leucine zipper kinase | 5.268 | Upregulated |
| 83 | IQGAP3 | IQ motif containing GTPase activating protein 3 | 5.241 | Upregulated |
| 84 | CENPA | centromere protein A | 5.107 | Upregulated |
| 85 | DLEU2 | deleted in lymphocytic leukemia 2 | 5.083 | Upregulated |
| 86 | UCN2 | urocortin 2 | 5.019 | Upregulated |
| 87 | CDCA5 | cell division cycle associated 5 | 5.014 | Upregulated |
| 88 | NCAPG2 | non-SMC condensin II complex subunit G2 | 4.957 | Upregulated |
| 89 | RRAS | RAS related | 4.951 | Upregulated |
| 90 | B3GALT2 | beta-1,3-galactosyltransferase 2 | 4.792 | Upregulated |
| 91 | CLSPN | claspin | 4.791 | Upregulated |
| 92 | FOXM1 | forkhead box M1 | 4.787 | Upregulated |
| 93 | TPX2 | TPX2 microtubule nucleation factor | 4.756 | Upregulated |
| 94 | KIF4A | kinesin family member 4A | 4.728 | Upregulated |
| 95 | ZWINT | ZW10 interacting kinetochore protein | 4.634 | Upregulated |
| 96 | LAPTM5 | lysosomal protein transmembrane 5 | 4.625 | Upregulated |
| 97 | CDCA7 | cell division cycle associated 7 | 4.620 | Upregulated |
| 98 | PTPN22 | protein tyrosine phosphatase non-receptor type 22 | 4.598 | Upregulated |
| 99 | FAM64A |  | 4.528 | Upregulated |
| 100 | FAM83D | family with sequence similarity 83 member D | 4.522 | Upregulated |
| 101 | GTSE1 | G2 and S-phase expressed 1 | 4.492 | Upregulated |
| 102 | MILR1 | mast cell immunoglobulin like receptor 1 | 4.443 | Upregulated |
| 103 | LINC00052 | long intergenic non-protein coding RNA 52 | 4.401 | Upregulated |
| 104 | E2F8 | E2F transcription factor 8 | 4.400 | Upregulated |
| 105 | FLNB | filamin B | 4.399 | Upregulated |
| 106 | SPAG5 | sperm associated antigen 5 | 4.387 | Upregulated |
| 107 | CENPF | centromere protein F | 4.374 | Upregulated |
| 108 | LOXL2 | lysyl oxidase like 2 | 4.369 | Upregulated |
| 109 | CHAF1B | chromatin assembly factor 1 subunit B | 4.354 | Upregulated |
| 110 | ECT2 | epithelial cell transforming 2 | 4.323 | Upregulated |
| 111 | TROAP | trophinin associated protein | 4.318 | Upregulated |
| 112 | RAD54L | RAD54 like | 4.312 | Upregulated |
| 113 | CDC6 | cell division cycle 6 | 4.260 | Upregulated |
| 114 | KIF18A | kinesin family member 18A | 4.245 | Upregulated |
| 115 | STIL | STIL centriolar assembly protein | 4.207 | Upregulated |
| 116 | CD302 | CD302 molecule | 4.198 | Upregulated |
| 117 | FAM198B |  | 4.180 | Upregulated |
| 118 | CKAP2 | cytoskeleton associated protein 2 | 4.093 | Upregulated |
| 119 | FANCD2 | FA complementation group D2 | 4.039 | Upregulated |
| 120 | PRIM1 | DNA primase subunit 1 | 4.034 | Upregulated |
| 121 | CCDC109B |  | 4.033 | Upregulated |
| 122 | AURKB | aurora kinase B | 4.002 | Upregulated |
| 123 | GBP4 | guanylate binding protein 4 | 3.965 | Upregulated |
| 124 | UHRF1 | ubiquitin like with PHD and ring finger domains 1 | 3.962 | Upregulated |
| 125 | SPC24 | SPC24 component of NDC80 kinetochore complex | 3.962 | Upregulated |
| 126 | AUNIP | aurora kinase A and ninein interacting protein | 3.944 | Upregulated |
| 127 | KIF18B | kinesin family member 18B | 3.918 | Upregulated |
| 128 | RTKN2 | rhotekin 2 | 3.899 | Upregulated |
| 129 | ERCC6L | ERCC excision repair 6 like, spindle assembly checkpoint helicase | 3.857 | Upregulated |
| 130 | DDAH1 | dimethylarginine dimethylaminohydrolase 1 | 3.824 | Upregulated |
| 131 | PFKFB4 | 6-phosphofructo-2-kinase/fructose-2,6-biphosphatase 4 | 3.799 | Upregulated |
| 132 | OGFRL1 | opioid growth factor receptor like 1 | 3.793 | Upregulated |
| 133 | GINS4 | GINS complex subunit 4 | 3.780 | Upregulated |
| 134 | ARHGAP11B | Rho GTPase activating protein 11B | 3.776 | Upregulated |
| 135 | CDC25C | cell division cycle 25C | 3.771 | Upregulated |
| 136 | PODXL | podocalyxin like | 3.741 | Upregulated |
| 137 | ENG | endoglin | 3.707 | Upregulated |
| 138 | NOV |  | 3.706 | Upregulated |
| 139 | RRM1 | ribonucleotide reductase catalytic subunit M1 | 3.665 | Upregulated |
| 140 | AURKA | aurora kinase A | 3.655 | Upregulated |
| 141 | MCM5 | minichromosome maintenance complex component 5 | 3.653 | Upregulated |
| 142 | PROCR | protein C receptor | 3.645 | Upregulated |
| 143 | F2R | coagulation factor II thrombin receptor | 3.604 | Upregulated |
| 144 | BICC1 | BicC family RNA binding protein 1 | 3.590 | Upregulated |
| 145 | BORA | BORA aurora kinase A activator | 3.588 | Upregulated |
| 146 | AXL | AXL receptor tyrosine kinase | 3.555 | Upregulated |
| 147 | TACC3 | transforming acidic coiled-coil containing protein 3 | 3.540 | Upregulated |
| 148 | PDK1 | pyruvate dehydrogenase kinase 1 | 3.513 | Upregulated |
| 149 | TAF9B | TATA-box binding protein associated factor 9b | 3.498 | Upregulated |
| 150 | ATAD5 | ATPase family AAA domain containing 5 | 3.470 | Upregulated |
| 151 | TCF19 | transcription factor 19 | 3.463 | Upregulated |
| 152 | UBE2T | ubiquitin conjugating enzyme E2 T | 3.420 | Upregulated |
| 153 | STC1 | stanniocalcin 1 | 3.413 | Upregulated |
| 154 | SPDL1 | spindle apparatus coiled-coil protein 1 | 3.405 | Upregulated |
| 155 | DEPDC4 | DEP domain containing 4 | 3.400 | Upregulated |
| 156 | HEG1 | heart development protein with EGF like domains 1 | 3.398 | Upregulated |
| 157 | LYN | LYN proto-oncogene, Src family tyrosine kinase | 3.391 | Upregulated |
| 158 | PDIA4 | protein disulfide isomerase family A member 4 | 3.390 | Upregulated |
| 159 | CCDC36 | coiled-coil domain containing 36 | 3.387 | Upregulated |
| 160 | ORC1 | origin recognition complex subunit 1 | 3.379 | Upregulated |
| 161 | FANCB | FA complementation group B | 3.347 | Upregulated |
| 162 | B3GNT5 | UDP-GlcNAc:betaGal beta-1,3-N-acetylglucosaminyltransferase 5 | 3.338 | Upregulated |
| 163 | SFXN2 | sideroflexin 2 | 3.332 | Upregulated |
| 164 | ARRDC4 | arrestin domain containing 4 | 3.315 | Upregulated |
| 165 | TENC1 |  | 3.312 | Upregulated |
| 166 | RAC2 | Rac family small GTPase 2 | 3.306 | Upregulated |
| 167 | SAMD9L | sterile alpha motif domain containing 9 like | 3.297 | Upregulated |
| 168 | OR4F15 | olfactory receptor family 4 subfamily F member 15 | 3.296 | Upregulated |
| 169 | ART3 | ADP-ribosyltransferase 3 | 3.295 | Upregulated |
| 170 | TRAM2 | translocation associated membrane protein 2 | 3.266 | Upregulated |
| 171 | CARHSP1 | calcium regulated heat stable protein 1 | 3.263 | Upregulated |
| 172 | ESPL1 | extra spindle pole bodies like 1, separase | 3.260 | Upregulated |
| 173 | ADAM19 | ADAM metallopeptidase domain 19 | 3.252 | Upregulated |
| 174 | TNFSF10 | TNF superfamily member 10 | 3.245 | Upregulated |
| 175 | RNASEH2A | ribonuclease H2 subunit A | 3.213 | Upregulated |
| 176 | LOC81691 |  | 3.193 | Upregulated |
| 177 | CDC25A | cell division cycle 25A | 3.180 | Upregulated |
| 178 | ADA | adenosine deaminase | 3.172 | Upregulated |
| 179 | TINAGL1 | tubulointerstitial nephritis antigen like 1 | 3.153 | Upregulated |
| 180 | MTFR2 | mitochondrial fission regulator 2 | 3.153 | Upregulated |
| 181 | FAM111A | family with sequence similarity 111 member A | 3.137 | Upregulated |
| 182 | C18orf54 | chromosome 18 open reading frame 54 | 3.117 | Upregulated |
| 183 | TNC | tenascin C | 3.104 | Upregulated |
| 184 | MCM4 | minichromosome maintenance complex component 4 | 3.099 | Upregulated |
| 185 | UBD | ubiquitin D | 3.066 | Upregulated |
| 186 | HMGB2 | high mobility group box 2 | 3.059 | Upregulated |
| 187 | PDIA5 | protein disulfide isomerase family A member 5 | 3.024 | Upregulated |
| 188 | NRM | nurim | 2.997 | Upregulated |
| 189 | CCNE1 | cyclin E1 | 2.993 | Upregulated |
| 190 | FAP | fibroblast activation protein alpha | 2.992 | Upregulated |
| 191 | SLC7A11 | solute carrier family 7 member 11 | 2.986 | Upregulated |
| 192 | GJA5 | gap junction protein alpha 5 | 2.985 | Upregulated |
| 193 | TRIM14 | tripartite motif containing 14 | 2.981 | Upregulated |
| 194 | RFTN1 | raftlin, lipid raft linker 1 | 2.964 | Upregulated |
| 195 | ARNTL2 | aryl hydrocarbon receptor nuclear translocator like 2 | 2.953 | Upregulated |
| 196 | RFC5 | replication factor C subunit 5 | 2.946 | Upregulated |
| 197 | FLJ36840 |  | 2.930 | Upregulated |
| 198 | SMC4 | structural maintenance of chromosomes 4 | 2.926 | Upregulated |
| 199 | ENPP1 | ectonucleotide pyrophosphatase/phosphodiesterase 1 | 2.889 | Upregulated |
| 200 | CDCA3 | cell division cycle associated 3 | 2.884 | Upregulated |
| 201 | LMO7 | LIM domain 7 | 2.875 | Upregulated |
| 202 | ECM1 | extracellular matrix protein 1 | 2.872 | Upregulated |
| 203 | UCP2 | uncoupling protein 2 | 2.864 | Upregulated |
| 204 | MTBP | MDM2 binding protein | 2.840 | Upregulated |
| 205 | PKMYT1 | protein kinase, membrane associated tyrosine/threonine 1 | 2.834 | Upregulated |
| 206 | RACGAP1 | Rac GTPase activating protein 1 | 2.824 | Upregulated |
| 207 | MOV10 | Mov10 RISC complex RNA helicase | 2.801 | Upregulated |
| 208 | ACBD7 | acyl-CoA binding domain containing 7 | 2.795 | Upregulated |
| 209 | REEP4 | receptor accessory protein 4 | 2.792 | Upregulated |
| 210 | FBXO5 | F-box protein 5 | 2.788 | Upregulated |
| 211 | RFX5 | regulatory factor X5 | 2.783 | Upregulated |
| 212 | LRRCC1 | leucine rich repeat and coiled-coil centrosomal protein 1 | 2.774 | Upregulated |
| 213 | VGLL3 | vestigial like family member 3 | 2.770 | Upregulated |
| 214 | PSMC3IP | PSMC3 interacting protein | 2.765 | Upregulated |
| 215 | MYO19 | myosin XIX | 2.748 | Upregulated |
| 216 | NCAPD3 | non-SMC condensin II complex subunit D3 | 2.741 | Upregulated |
| 217 | SNAPC1 | small nuclear RNA activating complex polypeptide 1 | 2.738 | Upregulated |
| 218 | C17orf53 |  | 2.734 | Upregulated |
| 219 | UBE2S | ubiquitin conjugating enzyme E2 S | 2.694 | Upregulated |
| 220 | KIF22 | kinesin family member 22 | 2.691 | Upregulated |
| 221 | TRIM59 | tripartite motif containing 59 | 2.687 | Upregulated |
| 222 | CTSS | cathepsin S | 2.669 | Upregulated |
| 223 | NCAPD2 | non-SMC condensin I complex subunit D2 | 2.668 | Upregulated |
| 224 | POLH | DNA polymerase eta | 2.650 | Upregulated |
| 225 | POLA2 | DNA polymerase alpha 2, accessory subunit | 2.632 | Upregulated |
| 226 | CEP135 | centrosomal protein 135 | 2.613 | Upregulated |
| 227 | KNTC1 | kinetochore associated 1 | 2.609 | Upregulated |
| 228 | PRPS2 | phosphoribosyl pyrophosphate synthetase 2 | 2.598 | Upregulated |
| 229 | TFDP1 | transcription factor Dp-1 | 2.576 | Upregulated |
| 230 | CHAF1A | chromatin assembly factor 1 subunit A | 2.543 | Upregulated |
| 231 | TIMELESS | timeless circadian regulator | 2.541 | Upregulated |
| 232 | FKBP11 | FKBP prolyl isomerase 11 | 2.535 | Upregulated |
| 233 | MCM2 | minichromosome maintenance complex component 2 | 2.529 | Upregulated |
| 234 | NLN | neurolysin | 2.520 | Upregulated |
| 235 | DGKA | diacylglycerol kinase alpha | 2.502 | Upregulated |
| 236 | CHST6 | carbohydrate sulfotransferase 6 | 2.490 | Upregulated |
| 237 | ADAM23 | ADAM metallopeptidase domain 23 | 2.489 | Upregulated |
| 238 | HMGB3 | high mobility group box 3 | 2.466 | Upregulated |
| 239 | MIS18BP1 | MIS18 binding protein 1 | 2.461 | Upregulated |
| 240 | CSF1 | colony stimulating factor 1 | 2.450 | Upregulated |
| 241 | ECE1 | endothelin converting enzyme 1 | 2.437 | Upregulated |
| 242 | UBAP2L | ubiquitin associated protein 2 like | 2.423 | Upregulated |
| 243 | LYZ | lysozyme | 2.422 | Upregulated |
| 244 | RBBP9 | RB binding protein 9, serine hydrolase | 2.393 | Upregulated |
| 245 | HIST2H2AB |  | 2.386 | Upregulated |
| 246 | RMI1 | RecQ mediated genome instability 1 | 2.385 | Upregulated |
| 247 | ZNF93 | zinc finger protein 93 | 2.376 | Upregulated |
| 248 | CCNF | cyclin F | 2.371 | Upregulated |
| 249 | ERAP1 | endoplasmic reticulum aminopeptidase 1 | 2.356 | Upregulated |
| 250 | HAUS1 | HAUS augmin like complex subunit 1 | 2.351 | Upregulated |
| 251 | ANO10 | anoctamin 10 | 2.350 | Upregulated |
| 252 | GRAMD1C | GRAM domain containing 1C | 2.350 | Upregulated |
| 253 | PARP8 | poly(ADP-ribose) polymerase family member 8 | 2.346 | Upregulated |
| 254 | MAGOHB | mago homolog B, exon junction complex subunit | 2.341 | Upregulated |
| 255 | TSPAN13 | tetraspanin 13 | 2.337 | Upregulated |
| 256 | CEP78 | centrosomal protein 78 | 2.332 | Upregulated |
| 257 | E2F7 | E2F transcription factor 7 | 2.326 | Upregulated |
| 258 | SGOL2 |  | 2.320 | Upregulated |
| 259 | GALE | UDP-galactose-4-epimerase | 2.319 | Upregulated |
| 260 | SCP2 | sterol carrier protein 2 | 2.314 | Upregulated |
| 261 | EZH2 | enhancer of zeste 2 polycomb repressive complex 2 subunit | 2.314 | Upregulated |
| 262 | FAS | Fas cell surface death receptor | 2.297 | Upregulated |
| 263 | GMNN | geminin DNA replication inhibitor | 2.294 | Upregulated |
| 264 | SUV39H1 | suppressor of variegation 3-9 homolog 1 | 2.294 | Upregulated |
| 265 | SRPX2 | sushi repeat containing protein X-linked 2 | 2.281 | Upregulated |
| 266 | CASP6 | caspase 6 | 2.267 | Upregulated |
| 267 | NDNF | neuron derived neurotrophic factor | 2.266 | Upregulated |
| 268 | GINS3 | GINS complex subunit 3 | 2.260 | Upregulated |
| 269 | DCBLD1 | discoidin, CUB and LCCL domain containing 1 | 2.259 | Upregulated |
| 270 | TRIP10 | thyroid hormone receptor interactor 10 | 2.248 | Upregulated |
| 271 | TRIM16L | tripartite motif containing 16 like | 2.247 | Upregulated |
| 272 | LOC100288637 |  | 2.227 | Upregulated |
| 273 | PCNA | proliferating cell nuclear antigen | 2.227 | Upregulated |
| 274 | MRPL28 | mitochondrial ribosomal protein L28 | 2.217 | Upregulated |
| 275 | LINC00268 | long intergenic non-protein coding RNA 268 | 2.211 | Upregulated |
| 276 | LAMB3 | laminin subunit beta 3 | 2.196 | Upregulated |
| 277 | MIR32 | microRNA 32 | 2.194 | Upregulated |
| 278 | TMEM194B |  | 2.192 | Upregulated |
| 279 | CCP110 | centriolar coiled-coil protein 110 | 2.190 | Upregulated |
| 280 | SYDE1 | synapse defective Rho GTPase homolog 1 | 2.185 | Upregulated |
| 281 | TSTA3 | tissue specific transplantation antigen P35B | 2.183 | Upregulated |
| 282 | DHTKD1 | dehydrogenase E1 and transketolase domain containing 1 | 2.175 | Upregulated |
| 283 | CERCAM | cerebral endothelial cell adhesion molecule | 2.171 | Upregulated |
| 284 | TMEM107 | transmembrane protein 107 | 2.168 | Upregulated |
| 285 | PHF11 | PHD finger protein 11 | 2.162 | Upregulated |
| 286 | NBN | nibrin | 2.160 | Upregulated |
| 287 | GPCPD1 | glycerophosphocholine phosphodiesterase 1 | 2.155 | Upregulated |
| 288 | RANBP1 | RAN binding protein 1 | 2.151 | Upregulated |
| 289 | ITGB5 | integrin subunit beta 5 | 2.139 | Upregulated |
| 290 | ERI2 | ERI1 exoribonuclease family member 2 | 2.138 | Upregulated |
| 291 | DONSON | downstream neighbor of SON | 2.137 | Upregulated |
| 292 | PXMP2 | peroxisomal membrane protein 2 | 2.125 | Upregulated |
| 293 | MAMDC2 | MAM domain containing 2 | 2.121 | Upregulated |
| 294 | TPM4 | tropomyosin 4 | 2.117 | Upregulated |
| 295 | MYLK | myosin light chain kinase | 2.114 | Upregulated |
| 296 | BAG2 | BAG cochaperone 2 | 2.111 | Upregulated |
| 297 | OR4F13P | olfactory receptor family 4 subfamily F member 13 pseudogene | 2.107 | Upregulated |
| 298 | AKAP6 | A-kinase anchoring protein 6 | 2.101 | Upregulated |
| 299 | ERLIN1 | ER lipid raft associated 1 | 2.101 | Upregulated |
| 300 | FANCE | FA complementation group E | 2.087 | Upregulated |
| 301 | LOC100132099 |  | 2.084 | Upregulated |
| 302 | SMC2 | structural maintenance of chromosomes 2 | 2.074 | Upregulated |
| 303 | PLOD1 | procollagen-lysine,2-oxoglutarate 5-dioxygenase 1 | 2.072 | Upregulated |
| 304 | ARHGDIB | Rho GDP dissociation inhibitor beta | 2.072 | Upregulated |
| 305 | EMP2 | epithelial membrane protein 2 | 2.069 | Upregulated |
| 306 | RPP40 | ribonuclease P/MRP subunit p40 | 2.062 | Upregulated |
| 307 | BRCA2 | BRCA2 DNA repair associated | 2.061 | Upregulated |
| 308 | KIF20B | kinesin family member 20B | 2.057 | Upregulated |
| 309 | SGCB | sarcoglycan beta | 2.054 | Upregulated |
| 310 | EXOC6 | exocyst complex component 6 | 2.052 | Upregulated |
| 311 | ZGRF1 | zinc finger GRF-type containing 1 | 2.051 | Upregulated |
| 312 | SNRPA | small nuclear ribonucleoprotein polypeptide A | 2.050 | Upregulated |
| 313 | RUVBL2 | RuvB like AAA ATPase 2 | 2.049 | Upregulated |
| 314 | OR4F21 | olfactory receptor family 4 subfamily F member 21 | 2.045 | Upregulated |
| 315 | ITGA6 | integrin subunit alpha 6 | 2.039 | Upregulated |
| 316 | GGH | gamma-glutamyl hydrolase | 2.035 | Upregulated |
| 317 | RPGRIP1L | RPGRIP1 like | 2.035 | Upregulated |
| 318 | SMAGP | small cell adhesion glycoprotein | 2.032 | Upregulated |
| 319 | KIF4B | kinesin family member 4B | 2.029 | Upregulated |
| 320 | DNA2 | DNA replication helicase/nuclease 2 | 2.028 | Upregulated |
| 321 | TWSG1 | twisted gastrulation BMP signaling modulator 1 | 2.024 | Upregulated |
| 322 | LDLRAP1 | low density lipoprotein receptor adaptor protein 1 | 2.018 | Upregulated |
| 323 | PDLIM7 | PDZ and LIM domain 7 | 2.012 | Upregulated |
| 324 | C1orf112 | chromosome 1 open reading frame 112 | 2.008 | Upregulated |
| 325 | DDB2 | damage specific DNA binding protein 2 | 2.008 | Upregulated |
| 326 | MIS18A | MIS18 kinetochore protein A | 2.008 | Upregulated |
| 327 | LOC100133130 |  | 2.001 | Upregulated |
| 328 | TCIRG1 |  | 1.996 | Upregulated |
| 329 | IFITM2 | T cell immune regulator 1, ATPase H+ transporting V0 subunit a3 | 1.987 | Upregulated |
| 330 | BARD1 | interferon induced transmembrane protein 2 | 1.986 | Upregulated |
| 331 | GPR126 | BRCA1 associated RING domain 1 | 1.974 | Upregulated |
| 332 | AMOT | angiomotin | 1.964 | Upregulated |
| 333 | DLD | dihydrolipoamide dehydrogenase | 1.962 | Upregulated |
| 334 | ZNF774 | zinc finger protein 774 | 1.960 | Upregulated |
| 335 | IRF1 | interferon regulatory factor 1 | 1.957 | Upregulated |
| 336 | CEP170P1 | centrosomal protein 170 pseudogene 1 | 1.954 | Upregulated |
| 337 | LINC00328 | long intergenic non-protein coding RNA 328 | 1.953 | Upregulated |
| 338 | STGC3 |  | 1.950 | Upregulated |
| 339 | GRAMD3 |  | 1.947 | Upregulated |
| 340 | DDX12P | DEAD/H-box helicase 12, pseudogene | 1.938 | Upregulated |
| 341 | CBR1 | carbonyl reductase 1 | 1.936 | Upregulated |
| 342 | DNMT1 | DNA methyltransferase 1 | 1.935 | Upregulated |
| 343 | MCM7 | minichromosome maintenance complex component 7 | 1.933 | Upregulated |
| 344 | FAM171B | family with sequence similarity 171 member B | 1.932 | Upregulated |
| 345 | CHAC2 | ChaC cation transport regulator homolog 2 | 1.932 | Upregulated |
| 346 | OSBPL10 | oxysterol binding protein like 10 | 1.924 | Upregulated |
| 347 | MDM2 | MDM2 proto-oncogene | 1.915 | Upregulated |
| 348 | BNIP3 | BCL2 interacting protein 3 | 1.913 | Upregulated |
| 349 | CEP41 | centrosomal protein 41 | 1.913 | Upregulated |
| 350 | GLMN | glomulin, FKBP associated protein | 1.909 | Upregulated |
| 351 | MPRIP | myosin phosphatase Rho interacting protein | 1.908 | Upregulated |
| 352 | MTF2 | metal response element binding transcription factor 2 | 1.907 | Upregulated |
| 353 | HNRNPA1 | heterogeneous nuclear ribonucleoprotein A1 | 1.901 | Upregulated |
| 354 | DCUN1D1 | defective in cullin neddylation 1 domain containing 1 | 1.898 | Upregulated |
| 355 | MBD3L5 | methyl-CpG binding domain protein 3 like 5 | 1.896 | Upregulated |
| 356 | ZNF100 | zinc finger protein 100 | 1.889 | Upregulated |
| 357 | WDR54 | WD repeat domain 54 | 1.887 | Upregulated |
| 358 | LPCAT4 | lysophosphatidylcholine acyltransferase 4 | 1.885 | Upregulated |
| 359 | LOC100133299 |  | 1.876 | Upregulated |
| 360 | PFDN1 | prefoldin subunit 1 | 1.871 | Upregulated |
| 361 | TMEM168 | transmembrane protein 168 | 1.871 | Upregulated |
| 362 | TOPBP1 | DNA topoisomerase II binding protein 1 | 1.864 | Upregulated |
| 363 | SMTN | smoothelin | 1.863 | Upregulated |
| 364 | STAG3L2 | stromal antigen 3-like 2 (pseudogene) | 1.860 | Upregulated |
| 365 | C1R | complement C1r | 1.859 | Upregulated |
| 366 | PSME2 | proteasome activator subunit 2 | 1.847 | Upregulated |
| 367 | TPI1 | triosephosphate isomerase 1 | 1.842 | Upregulated |
| 368 | LOC100131541 |  | 1.835 | Upregulated |
| 369 | EHF | ETS homologous factor | 1.832 | Upregulated |
| 370 | NEDD1 | NEDD1 gamma-tubulin ring complex targeting factor | 1.821 | Upregulated |
| 371 | QSER1 | glutamine and serine rich 1 | 1.820 | Upregulated |
| 372 | FAM57A |  | 1.819 | Upregulated |
| 373 | PEX11B | peroxisomal biogenesis factor 11 beta | 1.815 | Upregulated |
| 374 | KIAA0040 | KIAA0040 | 1.815 | Upregulated |
| 375 | SMAD3 | SMAD family member 3 | 1.807 | Upregulated |
| 376 | LRRN3 | leucine rich repeat neuronal 3 | 1.802 | Upregulated |
| 377 | RAB27B | RAB27B, member RAS oncogene family | 1.793 | Upregulated |
| 378 | TRIM68 | tripartite motif containing 68 | 1.781 | Upregulated |
| 379 | SLC25A15 | solute carrier family 25 member 15 | 1.773 | Upregulated |
| 380 | ADAM9 | ADAM metallopeptidase domain 9 | 1.773 | Upregulated |
| 381 | TBC1D1 | TBC1 domain family member 1 | 1.772 | Upregulated |
| 382 | GXYLT1 | glucoside xylosyltransferase 1 | 1.764 | Upregulated |
| 383 | ARHGEF26 | Rho guanine nucleotide exchange factor 26 | 1.764 | Upregulated |
| 384 | SVIL | supervillin | 1.761 | Upregulated |
| 385 | SHOX2 | short stature homeobox 2 | 1.761 | Upregulated |
| 386 | METTL7B | methyltransferase like 7B | 1.757 | Upregulated |
| 387 | NDC1 | NDC1 transmembrane nucleoporin | 1.749 | Upregulated |
| 388 | PGAM1 |  | 1.746 | Upregulated |
| 389 | GMPS | guanine monophosphate synthase | 1.740 | Upregulated |
| 390 | STRA6 | signaling receptor and transporter of retinol STRA6 | 1.740 | Upregulated |
| 391 | MANEA | mannosidase endo-alpha | 1.739 | Upregulated |
| 392 | HHEX | hematopoietically expressed homeobox | 1.735 | Upregulated |
| 393 | SRPK1 | SRSF protein kinase 1 | 1.735 | Upregulated |
| 394 | AGK | acylglycerol kinase | 1.733 | Upregulated |
| 395 | NOL12 | nucleolar protein 12 | 1.730 | Upregulated |
| 396 | PCBP4 | poly(rC) binding protein 4 | 1.720 | Upregulated |
| 397 | CCDC138 | coiled-coil domain containing 138 | 1.712 | Upregulated |
| 398 | B4GALNT3 | beta-1,4-N-acetyl-galactosaminyltransferase 3 | 1.711 | Upregulated |
| 399 | LIMA1 | LIM domain and actin binding 1 | 1.708 | Upregulated |
| 400 | HILPDA | hypoxia inducible lipid droplet associated | 1.704 | Upregulated |
| 401 | TMEM214 | transmembrane protein 214 | 1.704 | Upregulated |
| 402 | ZFP30 | ZFP30 zinc finger protein | 1.703 | Upregulated |
| 403 | RHNO1 | RAD9-HUS1-RAD1 interacting nuclear orphan 1 | 1.695 | Upregulated |
| 404 | NBEAL2 | neurobeachin like 2 | 1.694 | Upregulated |
| 405 | CMSS1 | cms1 ribosomal small subunit homolog | 1.688 | Upregulated |
| 406 | FN1 | fibronectin 1 | 1.682 | Upregulated |
| 407 | RPS6KA5 | ribosomal protein S6 kinase A5 | 1.679 | Upregulated |
| 408 | ENTHD1 | ENTH domain containing 1 | 1.678 | Upregulated |
| 409 | C4orf27 |  | 1.672 | Upregulated |
| 410 | LDHB | lactate dehydrogenase B | 1.664 | Upregulated |
| 411 | FNTB | farnesyltransferase, CAAX box, beta | 1.650 | Upregulated |
| 412 | SMC3 | structural maintenance of chromosomes 3 | 1.649 | Upregulated |
| 413 | ZNF544 | zinc finger protein 544 | 1.649 | Upregulated |
| 414 | MTMR2 | myotubularin related protein 2 | 1.645 | Upregulated |
| 415 | SLC17A9 | solute carrier family 17 member 9 | 1.645 | Upregulated |
| 416 | CENPJ | centromere protein J | 1.644 | Upregulated |
| 417 | CDC7 | cell division cycle 7 | 1.638 | Upregulated |
| 418 | RFFL | ring finger and FYVE like domain containing E3 ubiquitin protein ligase | 1.637 | Upregulated |
| 419 | ADSL | adenylosuccinate lyase | 1.636 | Upregulated |
| 420 | PXYLP1 | 2-phosphoxylose phosphatase 1 | 1.635 | Upregulated |
| 421 | FHDC1 | FH2 domain containing 1 | 1.625 | Upregulated |
| 422 | ZNF639 | zinc finger protein 639 | 1.623 | Upregulated |
| 423 | HIST1H2AK |  | 1.617 | Upregulated |
| 424 | CFL2 | cofilin 2 | 1.607 | Upregulated |
| 425 | CDCA4 | cell division cycle associated 4 | 1.606 | Upregulated |
| 426 | SERPINA5 | serpin family A member 5 | 1.602 | Upregulated |
| 427 | VOPP1 | VOPP1 WW domain binding protein | 1.599 | Upregulated |
| 428 | NUP107 | nucleoporin 107 | 1.598 | Upregulated |
| 429 | CD151 | CD151 molecule (Raph blood group) | 1.592 | Upregulated |
| 430 | ALG6 | ALG6 alpha-1,3-glucosyltransferase | 1.577 | Upregulated |
| 431 | ACER3 | alkaline ceramidase 3 | 1.577 | Upregulated |
| 432 | SH3RF1 | SH3 domain containing ring finger 1 | 1.576 | Upregulated |
| 433 | LGALS9 | galectin 9 | 1.575 | Upregulated |
| 434 | ZNF221 | zinc finger protein 221 | 1.574 | Upregulated |
| 435 | CEP170 | centrosomal protein 170 | 1.571 | Upregulated |
| 436 | RFWD3 | ring finger and WD repeat domain 3 | 1.569 | Upregulated |
| 437 | DEK | DEK proto-oncogene | 1.568 | Upregulated |
| 438 | PGAM1 | phosphoglycerate mutase 1 | 1.568 | Upregulated |
| 439 | TTLL4 | tubulin tyrosine ligase like 4 | 1.563 | Upregulated |
| 440 | BCKDHB | branched chain keto acid dehydrogenase E1 subunit beta | 1.562 | Upregulated |
| 441 | AGA | aspartylglucosaminidase | 1.559 | Upregulated |
| 442 | CCNC | cyclin C | 1.557 | Upregulated |
| 443 | BST1 | bone marrow stromal cell antigen 1 | 1.551 | Upregulated |
| 444 | AGPS | alkylglycerone phosphate synthase | 1.547 | Upregulated |
| 445 | CTSZ | cathepsin Z | 1.547 | Upregulated |
| 446 | CD97 |  | 1.541 | Upregulated |
| 447 | KATNAL1 | katanin catalytic subunit A1 like 1 | 1.539 | Upregulated |
| 448 | FGF7 | fibroblast growth factor 7 | 1.539 | Upregulated |
| 449 | FRMD6 | FERM domain containing 6 | 1.537 | Upregulated |
| 450 | ULBP2 | UL16 binding protein 2 | 1.536 | Upregulated |
| 451 | DUS2 | dihydrouridine synthase 2 | 1.535 | Upregulated |
| 452 | PAICS | phosphoribosylaminoimidazole carboxylase and phosphoribosylaminoimidazolesuccinocarboxamide synthase | 1.532 | Upregulated |
| 453 | ERO1L |  | 1.527 | Upregulated |
| 454 | RSL1D1 | ribosomal L1 domain containing 1 | 1.526 | Upregulated |
| 455 | KPNA3 | karyopherin subunit alpha 3 | 1.524 | Upregulated |
| 456 | GNPNAT1 | glucosamine-phosphate N-acetyltransferase 1 | 1.521 | Upregulated |
| 457 | TTC26 | tetratricopeptide repeat domain 26 | 1.521 | Upregulated |
| 458 | ITGB3BP | integrin subunit beta 3 binding protein | 1.519 | Upregulated |
| 459 | SSR3 | signal sequence receptor subunit 3 | 1.515 | Upregulated |
| 460 | IDE | insulin degrading enzyme | 1.515 | Upregulated |
| 461 | SLC25A5 | solute carrier family 25 member 5 | 1.507 | Upregulated |
| 462 | SLC37A1 | solute carrier family 37 member 1 | 1.501 | Upregulated |
| 463 | TP53 | tumor protein p53 | 1.501 | Upregulated |
| 464 | IPP | intracisternal A particle-promoted polypeptide | 1.500 | Upregulated |
| 465 | CDH2 | cadherin 2 | 1.499 | Upregulated |
| 466 | MIR15A | microRNA 15a | 1.495 | Upregulated |
| 467 | METAP1 | methionyl aminopeptidase 1 | 1.492 | Upregulated |
| 468 | XPO6 | exportin 6 | 1.492 | Upregulated |
| 469 | IKBIP | IKBKB interacting protein | 1.490 | Upregulated |
| 470 | PREP | prolyl endopeptidase | 1.483 | Upregulated |
| 471 | SMC1A | structural maintenance of chromosomes 1A | 1.472 | Upregulated |
| 472 | SH3D19 | SH3 domain containing 19 | 1.471 | Upregulated |
| 473 | GEMIN4 | gem nuclear organelle associated protein 4 | 1.471 | Upregulated |
| 474 | ZCCHC17 | zinc finger CCHC-type containing 17 | 1.458 | Upregulated |
| 475 | DEFB124 | defensin beta 124 | 1.453 | Upregulated |
| 476 | ADAMTS3 | ADAM metallopeptidase with thrombospondin type 1 motif 3 | 1.449 | Upregulated |
| 477 | CNIH2 | cornichon family AMPA receptor auxiliary protein 2 | 1.448 | Upregulated |
| 478 | IL13RA1 |  | 1.448 | Upregulated |
| 479 | SEPN1 | interleukin 13 receptor subunit alpha 1 | 1.447 | Upregulated |
| 480 | RAB11FIP2 | RAB11 family interacting protein 2 | 1.444 | Upregulated |
| 481 | HSPB11 | heat shock protein family B (small) member 11 | 1.435 | Upregulated |
| 482 | KCND1 | potassium voltage-gated channel subfamily D member 1 | 1.422 | Upregulated |
| 483 | HIVEP3 | HIVEP zinc finger 3 | 1.422 | Upregulated |
| 484 | GULP1 | GULP PTB domain containing engulfment adaptor 1 | 1.419 | Upregulated |
| 485 | HDAC7 | histone deacetylase 7 | 1.418 | Upregulated |
| 486 | CBX5 | chromobox 5 | 1.415 | Upregulated |
| 487 | MRE11A |  | 1.407 | Upregulated |
| 488 | ODF2L | outer dense fiber of sperm tails 2 like | 1.407 | Upregulated |
| 489 | IFT57 | intraflagellar transport 57 | 1.403 | Upregulated |
| 490 | MASTL | microtubule associated serine/threonine kinase like | 1.390 | Upregulated |
| 491 | TMEM185A | transmembrane protein 185A | 1.390 | Upregulated |
| 492 | ZNF844 | zinc finger protein 844 | 1.379 | Upregulated |
| 493 | SLC25A37 | solute carrier family 25 member 37 | 1.377 | Upregulated |
| 494 | PCOLCE2 | procollagen C-endopeptidase enhancer 2 | 1.370 | Upregulated |
| 495 | HLA-C | major histocompatibility complex, class I, C | 1.365 | Upregulated |
| 496 | AGO1 | argonaute RISC component 1 | 1.361 | Upregulated |
| 497 | ERAL1 | Era like 12S mitochondrial rRNA chaperone 1 | 1.359 | Upregulated |
| 498 | HDDC2 | HD domain containing 2 | 1.357 | Upregulated |
| 499 | NKIRAS2 | NFKB inhibitor interacting Ras like 2 | 1.349 | Upregulated |
| 500 | LMCD1 | LIM and cysteine rich domains 1 | 1.347 | Upregulated |
| 501 | PRDX3 | peroxiredoxin 3 | 1.345 | Upregulated |
| 502 | IL2RB | interleukin 2 receptor subunit beta | 1.343 | Upregulated |
| 503 | TGFA | transforming growth factor alpha | 1.341 | Upregulated |
| 504 | GCFC2 | GC-rich sequence DNA-binding factor 2 | 1.340 | Upregulated |
| 505 | SH3PXD2A | SH3 and PX domains 2A | 1.334 | Upregulated |
| 506 | UCA1 | urothelial cancer associated 1 | 1.330 | Upregulated |
| 507 | OXR1 | oxidation resistance 1 | 1.329 | Upregulated |
| 508 | IFI27L2 | interferon alpha inducible protein 27 like 2 | 1.325 | Upregulated |
| 509 | LOC100133106 |  | 1.325 | Upregulated |
| 510 | TBC1D10A | TBC1 domain family member 10A | 1.323 | Upregulated |
| 511 | MLF2 | myeloid leukemia factor 2 | 1.321 | Upregulated |
| 512 | XPO4 | exportin 4 | 1.318 | Upregulated |
| 513 | SMCO4 | single-pass membrane protein with coiled-coil domains 4 | 1.317 | Upregulated |
| 514 | CPD | carboxypeptidase D | 1.314 | Upregulated |
| 515 | SMAD7 | SMAD family member 7 | 1.312 | Upregulated |
| 516 | IPO5 | importin 5 | 1.309 | Upregulated |
| 517 | RAD18 | RAD18 E3 ubiquitin protein ligase | 1.308 | Upregulated |
| 518 | ZNF860 | zinc finger protein 860 | 1.307 | Upregulated |
| 519 | GTF3C6 | general transcription factor IIIC subunit 6 | 1.306 | Upregulated |
| 520 | MTFR1 | mitochondrial fission regulator 1 | 1.305 | Upregulated |
| 521 | MNAT1 | MNAT1 component of CDK activating kinase | 1.302 | Upregulated |
| 522 | KLRC2 | killer cell lectin like receptor C2 | 1.301 | Upregulated |
| 523 | KRTAP4-9 | keratin associated protein 4-9 | 1.294 | Upregulated |
| 524 | OTUD6B | OTU deubiquitinase 6B | 1.294 | Upregulated |
| 525 | RPN2 | ribophorin II | 1.287 | Upregulated |
| 526 | KIAA1217 | KIAA1217 | 1.281 | Upregulated |
| 527 | METTL10 |  | 1.280 | Upregulated |
| 528 | SPDYE8P |  | 1.278 | Upregulated |
| 529 | OSGEP | O-sialoglycoprotein endopeptidase | 1.274 | Upregulated |
| 530 | SIPA1L3 | signal induced proliferation associated 1 like 3 | 1.264 | Upregulated |
| 531 | ABCC3 | ATP binding cassette subfamily C member 3 | 1.248 | Upregulated |
| 532 | APOL3 | apolipoprotein L3 | 1.245 | Upregulated |
| 533 | CPPED1 | calcineurin like phosphoesterase domain containing 1 | 1.244 | Upregulated |
| 534 | DTD2 | D-aminoacyl-tRNA deacylase 2 | 1.238 | Upregulated |
| 535 | ASB8 | ankyrin repeat and SOCS box containing 8 | 1.233 | Upregulated |
| 536 | EFEMP2 | EGF containing fibulin extracellular matrix protein 2 | 1.233 | Upregulated |
| 537 | UBE2D4 | ubiquitin conjugating enzyme E2 D4 (putative) | 1.224 | Upregulated |
| 538 | CA12 | carbonic anhydrase 12 | 1.212 | Upregulated |
| 539 | GDNF | glial cell derived neurotrophic factor | 1.205 | Upregulated |
| 540 | SH3GLB1 | SH3 domain containing GRB2 like, endophilin B1 | 1.204 | Upregulated |
| 541 | VCAM1 | vascular cell adhesion molecule 1 | 1.202 | Upregulated |
| 542 | DKKL1 | dickkopf like acrosomal protein 1 | 1.192 | Upregulated |
| 543 | PLAGL1 | PLAG1 like zinc finger 1 | 1.185 | Upregulated |
| 544 | ESM1 | endothelial cell specific molecule 1 | 1.183 | Upregulated |
| 545 | LINC00189 | long intergenic non-protein coding RNA 189 | 1.172 | Upregulated |
| 546 | MIR181A2 | microRNA 181a-2 | 1.163 | Upregulated |
| 547 | FEZ1 | fasciculation and elongation protein zeta 1 | 1.157 | Upregulated |
| 548 | MT1M | metallothionein 1M | 1.152 | Upregulated |
| 549 | JDP2 | Jun dimerization protein 2 | 1.136 | Upregulated |
| 550 | ANO6 | anoctamin 6 | 1.121 | Upregulated |
| 551 | HSPB6 | heat shock protein family B (small) member 6 | 1.119 | Upregulated |
| 552 | WEE1 | WEE1 G2 checkpoint kinase | 1.115 | Upregulated |
| 553 | LRRC8B | leucine rich repeat containing 8 VRAC subunit B | 1.110 | Upregulated |
| 554 | RENBP | renin binding protein | 1.110 | Upregulated |
| 555 | URI1 | URI1 prefoldin like chaperone | 1.106 | Upregulated |
| 556 | SPAG4 | sperm associated antigen 4 | 1.095 | Upregulated |
| 557 | CAPN2 | calpain 2 | 1.094 | Upregulated |
| 558 | LRPPRC | leucine rich pentatricopeptide repeat containing | 1.092 | Upregulated |
| 559 | BMP2 | bone morphogenetic protein 2 | 1.092 | Upregulated |
| 560 | MGMT | O-6-methylguanine-DNA methyltransferase | 1.089 | Upregulated |
| 561 | ORC2 | origin recognition complex subunit 2 | 1.087 | Upregulated |
| 562 | CHN1 | chimerin 1 | 1.073 | Upregulated |

| 1 | PLXNC1 | plexin C1 | 0.042 | Downregulated |
| --- | --- | --- | --- | --- |
| 2 | ST8SIA6 | ST8 alpha-N-acetyl-neuraminide alpha-2,8-sialyltransferase 6 | 0.046 | Downregulated |
| 3 | SLC6A17 | solute carrier family 6 member 17 | 0.080 | Downregulated |
| 4 | RGS1 | regulator of G protein signaling 1 | 0.098 | Downregulated |
| 5 | CHN2 | chimerin 2 | 0.099 | Downregulated |
| 6 | APOC1 | apolipoprotein C1 | 0.142 | Downregulated |
| 7 | FOS | Fos proto-oncogene, AP-1 transcription factor subunit | 0.147 | Downregulated |
| 8 | SNORD82 | small nucleolar RNA, C/D box 82 | 0.150 | Downregulated |
| 9 | ITGA7 | integrin subunit alpha 7 | 0.159 | Downregulated |
| 10 | PDE3B | phosphodiesterase 3B | 0.162 | Downregulated |
| 11 | DNER | delta/notch like EGF repeat containing | 0.192 | Downregulated |
| 12 | KRTAP19-1 | keratin associated protein 19-1 | 0.200 | Downregulated |
| 13 | CHMP1B | charged multivesicular body protein 1B | 0.208 | Downregulated |
| 14 | SCD | stearoyl-CoA desaturase | 0.220 | Downregulated |
| 15 | PTGS2 | prostaglandin-endoperoxide synthase 2 | 0.223 | Downregulated |
| 16 | HMGCS1 | 3-hydroxy-3-methylglutaryl-CoA synthase 1 | 0.226 | Downregulated |
| 17 | KLHL24 | kelch like family member 24 | 0.228 | Downregulated |
| 18 | CFLAR | CASP8 and FADD like apoptosis regulator | 0.231 | Downregulated |
| 19 | WDR78 | WD repeat domain 78 | 0.236 | Downregulated |
| 20 | CLK1 | CDC like kinase 1 | 0.237 | Downregulated |
| 21 | ATP10A | ATPase phospholipid transporting 10A (putative) | 0.245 | Downregulated |
| 22 | NSG1 | neuronal vesicle trafficking associated 1 | 0.249 | Downregulated |
| 23 | RGS2 | regulator of G protein signaling 2 | 0.251 | Downregulated |
| 24 | ROBO2 | roundabout guidance receptor 2 | 0.252 | Downregulated |
| 25 | FOSB | FosB proto-oncogene, AP-1 transcription factor subunit | 0.261 | Downregulated |
| 26 | ZHX2 | zinc fingers and homeoboxes 2 | 0.263 | Downregulated |
| 27 | PDE4DIP | phosphodiesterase 4D interacting protein | 0.264 | Downregulated |
| 28 | BMP8B | bone morphogenetic protein 8b | 0.270 | Downregulated |
| 29 | RDH8 | retinol dehydrogenase 8 | 0.276 | Downregulated |
| 30 | ANO4 | anoctamin 4 | 0.280 | Downregulated |
| 31 | EPB41L4A-AS1 | EPB41L4A antisense RNA 1 | 0.280 | Downregulated |
| 32 | GPR158 | G protein-coupled receptor 158 | 0.282 | Downregulated |
| 33 | TMEM117 | transmembrane protein 117 | 0.284 | Downregulated |
| 34 | APOE | apolipoprotein E | 0.286 | Downregulated |
| 35 | TMA7 | translation machinery associated 7 homolog | 0.287 | Downregulated |
| 36 | ABCB5 | ATP binding cassette subfamily B member 5 | 0.288 | Downregulated |
| 37 | ILF3-AS1 |  | 0.288 | Downregulated |
| 38 | CTSH | cathepsin H | 0.296 | Downregulated |
| 39 | C6orf48 |  | 0.296 | Downregulated |
| 40 | EMP1 | epithelial membrane protein 1 | 0.297 | Downregulated |
| 41 | TMEM242 | transmembrane protein 242 | 0.299 | Downregulated |
| 42 | MAGI2 | membrane associated guanylate kinase, WW and PDZ domain containing 2 | 0.301 | Downregulated |
| 43 | MSMO1 | methylsterol monooxygenase 1 | 0.302 | Downregulated |
| 44 | NUPR1 | nuclear protein 1, transcriptional regulator | 0.302 | Downregulated |
| 45 | SNORD104 | small nucleolar RNA, C/D box 104 | 0.304 | Downregulated |
| 46 | C10orf11 |  | 0.305 | Downregulated |
| 47 | IGF1R | insulin like growth factor 1 receptor | 0.306 | Downregulated |
| 48 | OCA2 | OCA2 melanosomal transmembrane protein | 0.306 | Downregulated |
| 49 | SNORA20 | small nucleolar RNA, H/ACA box 20 | 0.306 | Downregulated |
| 50 | MLANA | melan-A | 0.310 | Downregulated |
| 51 | RAB17 | RAB17, member RAS oncogene family | 0.311 | Downregulated |
| 52 | SLCO4C1 | solute carrier organic anion transporter family member 4C1 | 0.311 | Downregulated |
| 53 | CENPBD1P1 | CENPB DNA-binding domains containing 1 pseudogene 1 | 0.312 | Downregulated |
| 54 | SEMA5A | semaphorin 5A | 0.316 | Downregulated |
| 55 | SLC2A3 | solute carrier family 2 member 3 | 0.317 | Downregulated |
| 56 | FBXO32 | F-box protein 32 | 0.317 | Downregulated |
| 57 | BMPR1B | bone morphogenetic protein receptor type 1B | 0.322 | Downregulated |
| 58 | EPHA5 | EPH receptor A5 | 0.322 | Downregulated |
| 59 | EPAS1 | endothelial PAS domain protein 1 | 0.322 | Downregulated |
| 60 | SEMA3D | semaphorin 3D | 0.326 | Downregulated |
| 61 | IRF4 | interferon regulatory factor 4 | 0.327 | Downregulated |
| 62 | FAM101B |  | 0.327 | Downregulated |
| 63 | RPS29 | ribosomal protein S29 | 0.330 | Downregulated |
| 64 | SNORD46 | small nucleolar RNA, C/D box 46 | 0.331 | Downregulated |
| 65 | HMOX1 | heme oxygenase 1 | 0.331 | Downregulated |
| 66 | SNORD15B | small nucleolar RNA, C/D box 15B | 0.332 | Downregulated |
| 67 | PIEZO2 | piezo type mechanosensitive ion channel component 2 | 0.333 | Downregulated |
| 68 | DUSP1 | dual specificity phosphatase 1 | 0.333 | Downregulated |
| 69 | PLA1A | phospholipase A1 member A | 0.334 | Downregulated |
| 70 | RGS20 | regulator of G protein signaling 20 | 0.334 | Downregulated |
| 71 | RPL22L1 | ribosomal protein L22 like 1 | 0.336 | Downregulated |
| 72 | SLC10A5 | solute carrier family 10 member 5 | 0.339 | Downregulated |
| 73 | CARD16 | caspase recruitment domain family member 16 | 0.342 | Downregulated |
| 74 | CREBRF | CREB3 regulatory factor | 0.344 | Downregulated |
| 75 | LSM14B | LSM family member 14B | 0.349 | Downregulated |
| 76 | PPP1R15A | protein phosphatase 1 regulatory subunit 15A | 0.352 | Downregulated |
| 77 | CHORDC1 | cysteine and histidine rich domain containing 1 | 0.353 | Downregulated |
| 78 | FAM214A | family with sequence similarity 214 member A | 0.355 | Downregulated |
| 79 | KLHL21 | kelch like family member 21 | 0.356 | Downregulated |
| 80 | BDH2 | 3-hydroxybutyrate dehydrogenase 2 | 0.358 | Downregulated |
| 81 | DNAJB4 | DnaJ heat shock protein family (Hsp40) member B4 | 0.360 | Downregulated |
| 82 | EXPH5 | exophilin 5 | 0.365 | Downregulated |
| 83 | MXD1 | MAX dimerization protein 1 | 0.366 | Downregulated |
| 84 | CAB39L | calcium binding protein 39 like | 0.369 | Downregulated |
| 85 | HSD17B7P2 | hydroxysteroid 17-beta dehydrogenase 7 pseudogene 2 | 0.376 | Downregulated |
| 86 | CYSTM1 | cysteine rich transmembrane module containing 1 | 0.378 | Downregulated |
| 87 | DISP1 | dispatched RND transporter family member 1 | 0.378 | Downregulated |
| 88 | KLKB1 | kallikrein B1 | 0.379 | Downregulated |
| 89 | SLC6A15 | solute carrier family 6 member 15 | 0.383 | Downregulated |
| 90 | NR1D1 | nuclear receptor subfamily 1 group D member 1 | 0.383 | Downregulated |
| 91 | RBM8A | RNA binding motif protein 8A | 0.386 | Downregulated |
| 92 | EIF1B | eukaryotic translation initiation factor 1B | 0.386 | Downregulated |
| 93 | IDI1 | isopentenyl-diphosphate delta isomerase 1 | 0.386 | Downregulated |
| 94 | KLHDC10 | kelch domain containing 10 | 0.387 | Downregulated |
| 95 | USMG5 |  | 0.387 | Downregulated |
| 96 | LRRC39 | leucine rich repeat containing 39 | 0.392 | Downregulated |
| 97 | TRIB1 | tribbles pseudokinase 1 | 0.392 | Downregulated |
| 98 | FLJ44896 |  | 0.392 | Downregulated |
| 99 | GMPR | guanosine monophosphate reductase | 0.393 | Downregulated |
| 100 | RAB7A | RAB7A, member RAS oncogene family | 0.396 | Downregulated |
| 101 | DPH3 | diphthamide biosynthesis 3 | 0.396 | Downregulated |
| 102 | PS1TP4 |  | 0.396 | Downregulated |
| 103 | ANAPC16 | anaphase promoting complex subunit 16 | 0.397 | Downregulated |
| 104 | C2orf76 | chromosome 2 open reading frame 76 | 0.399 | Downregulated |
| 105 | CTTNBP2 | cortactin binding protein 2 | 0.399 | Downregulated |
| 106 | SLCO5A1 | solute carrier organic anion transporter family member 5A1 | 0.399 | Downregulated |
| 107 | IL12RB2 | interleukin 12 receptor subunit beta 2 | 0.400 | Downregulated |
| 108 | OARD1 | O-acyl-ADP-ribose deacylase 1 | 0.403 | Downregulated |
| 109 | USMG5 |  | 0.404 | Downregulated |
| 110 | OTUD1 | OTU deubiquitinase 1 | 0.405 | Downregulated |
| 111 | HMGCR | 3-hydroxy-3-methylglutaryl-CoA reductase | 0.406 | Downregulated |
| 112 | HSPH1 | heat shock protein family H (Hsp110) member 1 | 0.406 | Downregulated |
| 113 | LYST | lysosomal trafficking regulator | 0.410 | Downregulated |
| 114 | PDGFA | platelet derived growth factor subunit A | 0.413 | Downregulated |
| 115 | KGFLP1 |  | 0.413 | Downregulated |
| 116 | C1orf21 | chromosome 1 open reading frame 21 | 0.418 | Downregulated |
| 117 | SNX9 | sorting nexin 9 | 0.418 | Downregulated |
| 118 | C12orf57 | chromosome 12 open reading frame 57 | 0.419 | Downregulated |
| 119 | PRKXP1 | PRKX pseudogene 1 | 0.419 | Downregulated |
| 120 | SNX30 | sorting nexin family member 30 | 0.420 | Downregulated |
| 121 | SNORD41 | small nucleolar RNA, C/D box 41 | 0.421 | Downregulated |
| 122 | C21orf91 | chromosome 21 open reading frame 91 | 0.424 | Downregulated |
| 123 | C9orf85 | chromosome 9 open reading frame 85 | 0.425 | Downregulated |
| 124 | HIPK3 | homeodomain interacting protein kinase 3 | 0.426 | Downregulated |
| 125 | MFSD6 | major facilitator superfamily domain containing 6 | 0.426 | Downregulated |
| 126 | LRIF1 | ligand dependent nuclear receptor interacting factor 1 | 0.426 | Downregulated |
| 127 | ZNF703 | zinc finger protein 703 | 0.427 | Downregulated |
| 128 | DSTYK | dual serine/threonine and tyrosine protein kinase | 0.427 | Downregulated |
| 129 | SCARNA17 | small Cajal body-specific RNA 17 | 0.427 | Downregulated |
| 130 | JOSD1 | Josephin domain containing 1 | 0.427 | Downregulated |
| 131 | ISCU | iron-sulfur cluster assembly enzyme | 0.428 | Downregulated |
| 132 | PFKFB2 | 6-phosphofructo-2-kinase/fructose-2,6-biphosphatase 2 | 0.428 | Downregulated |
| 133 | ETNK1 | ethanolamine kinase 1 | 0.429 | Downregulated |
| 134 | APOD | apolipoprotein D | 0.429 | Downregulated |
| 135 | PHF1 | PHD finger protein 1 | 0.430 | Downregulated |
| 136 | GPR137B | G protein-coupled receptor 137B | 0.430 | Downregulated |
| 137 | SV2B | synaptic vesicle glycoprotein 2B | 0.431 | Downregulated |
| 138 | EPG5 | ectopic P-granules autophagy protein 5 homolog | 0.431 | Downregulated |
| 139 | GLS | glutaminase | 0.431 | Downregulated |
| 140 | UBE2F | ubiquitin conjugating enzyme E2 F (putative) | 0.432 | Downregulated |
| 141 | NSMCE2 | NSE2 (MMS21) homolog, SMC5-SMC6 complex SUMO ligase | 0.433 | Downregulated |
| 142 | JMY | junction mediating and regulatory protein, p53 cofactor | 0.433 | Downregulated |
| 143 | EVA1A | eva-1 homolog A, regulator of programmed cell death | 0.433 | Downregulated |
| 144 | TRAPPC2 | trafficking protein particle complex 2 | 0.434 | Downregulated |
| 145 | RPSAP52 | ribosomal protein SA pseudogene 52 | 0.435 | Downregulated |
| 146 | ARID4A | AT-rich interaction domain 4A | 0.435 | Downregulated |
| 147 | FAM160A1 | family with sequence similarity 160 member A1 | 0.439 | Downregulated |
| 148 | RASSF2 | Ras association domain family member 2 | 0.441 | Downregulated |
| 149 | B3GNT2 | UDP-GlcNAc:betaGal beta-1,3-N-acetylglucosaminyltransferase 2 | 0.448 | Downregulated |
| 150 | CYTH3 | cytohesin 3 | 0.449 | Downregulated |
| 151 | PTPRK | protein tyrosine phosphatase receptor type K | 0.449 | Downregulated |
| 152 | CTSO | cathepsin O | 0.451 | Downregulated |
| 153 | KLHDC2 | kelch domain containing 2 | 0.451 | Downregulated |
| 154 | ABHD4 | abhydrolase domain containing 4 | 0.451 | Downregulated |
| 155 | ZNF622 | zinc finger protein 622 | 0.452 | Downregulated |
| 156 | RING1 | ring finger protein 1 | 0.452 | Downregulated |
| 157 | FAM213A |  | 0.455 | Downregulated |
| 158 | RAB38 | RAB38, member RAS oncogene family | 0.456 | Downregulated |
| 159 | ZFP36 | ZFP36 ring finger protein | 0.456 | Downregulated |
| 160 | ACAT2 | acetyl-CoA acetyltransferase 2 | 0.458 | Downregulated |
| 161 | DNAJB2 | DnaJ heat shock protein family (Hsp40) member B2 | 0.460 | Downregulated |
| 162 | OLFM2 | olfactomedin 2 | 0.462 | Downregulated |
| 163 | LINGO2 | leucine rich repeat and Ig domain containing 2 | 0.463 | Downregulated |
| 164 | HSPA1L | heat shock protein family A (Hsp70) member 1 like | 0.465 | Downregulated |
| 165 | TARSL2 |  | 0.465 | Downregulated |
| 166 | JMJD6 | jumonji domain containing 6, arginine demethylase and lysine hydroxylase | 0.465 | Downregulated |
| 167 | ZNF330 | zinc finger protein 330 | 0.466 | Downregulated |
| 168 | ZDHHC14 | zinc finger DHHC-type palmitoyltransferase 14 | 0.469 | Downregulated |
| 169 | FAM63B |  | 0.470 | Downregulated |
| 170 | ACSL1 | acyl-CoA synthetase long chain family member 1 | 0.470 | Downregulated |
| 171 | ATP6V0D1 | ATPase H+ transporting V0 subunit d1 | 0.473 | Downregulated |
| 172 | SLC30A1 | solute carrier family 30 member 1 | 0.473 | Downregulated |
| 173 | CHKA | choline kinase alpha | 0.474 | Downregulated |
| 174 | CIR1 | corepressor interacting with RBPJ, 1 | 0.474 | Downregulated |
| 175 | PEBP1 | phosphatidylethanolamine binding protein 1 | 0.476 | Downregulated |
| 176 | TTL | tubulin tyrosine ligase | 0.476 | Downregulated |
| 177 | CEBPB | CCAAT enhancer binding protein beta | 0.479 | Downregulated |
| 178 | SS18L1 | SS18L1 subunit of BAF chromatin remodeling complex | 0.479 | Downregulated |
| 179 | SMPD2 | sphingomyelin phosphodiesterase 2 | 0.481 | Downregulated |
| 180 | EGR1 | early growth response 1 | 0.482 | Downregulated |
| 181 | RMND5A | required for meiotic nuclear division 5 homolog A | 0.483 | Downregulated |
| 182 | TRAPPC13 | trafficking protein particle complex 13 | 0.484 | Downregulated |
| 183 | SARAF | store-operated calcium entry associated regulatory factor | 0.484 | Downregulated |
| 184 | DRAM1 | DNA damage regulated autophagy modulator 1 | 0.485 | Downregulated |
| 185 | GPRC5B | G protein-coupled receptor class C group 5 member B | 0.485 | Downregulated |
| 186 | TEX15 | testis expressed 15, meiosis and synapsis associated | 0.485 | Downregulated |
| 187 | NLK | nemo like kinase | 0.486 | Downregulated |
| 188 | BMPR2 | bone morphogenetic protein receptor type 2 | 0.486 | Downregulated |
| 189 | RXRB | retinoid X receptor beta | 0.486 | Downregulated |
| 190 | EFCAB13 | EF-hand calcium binding domain 13 | 0.488 | Downregulated |
| 191 | PFDN5 | prefoldin subunit 5 | 0.490 | Downregulated |
| 192 | TMA7 |  | 0.490 | Downregulated |
| 193 | SMOX | spermine oxidase | 0.491 | Downregulated |
| 194 | MFAP3L | microfibril associated protein 3 like | 0.492 | Downregulated |
| 195 | MGAT4B | alpha-1,3-mannosyl-glycoprotein 4-beta-N-acetylglucosaminyltransferase B | 0.493 | Downregulated |
| 196 | HSBP1 | heat shock factor binding protein 1 | 0.494 | Downregulated |
| 197 | LINC00518 | long intergenic non-protein coding RNA 518 | 0.494 | Downregulated |
| 198 | CTR9 | CTR9 homolog, Paf1/RNA polymerase II complex component | 0.494 | Downregulated |
| 199 | MAN2A2 | mannosidase alpha class 2A member 2 | 0.495 | Downregulated |
| 200 | PLA2G16 |  | 0.496 | Downregulated |
| 201 | C6orf62 | chromosome 6 open reading frame 62 | 0.496 | Downregulated |
| 202 | MUT |  | 0.497 | Downregulated |
| 203 | LARGE |  | 0.498 | Downregulated |
| 204 | EIF4B | eukaryotic translation initiation factor 4B | 0.498 | Downregulated |
| 205 | NT5C3A | 5'-nucleotidase, cytosolic IIIA | 0.499 | Downregulated |
| 206 | ZNF322 | zinc finger protein 322 | 0.499 | Downregulated |
| 207 | CFLAR |  | 0.501 | Downregulated |
| 208 | JUNB | JunB proto-oncogene, AP-1 transcription factor subunit | 0.502 | Downregulated |
| 209 | ZNF805 | zinc finger protein 805 | 0.503 | Downregulated |
| 210 | BHLHE40 | basic helix-loop-helix family member e40 | 0.504 | Downregulated |
| 211 | RNF114 | ring finger protein 114 | 0.505 | Downregulated |
| 212 | MLPH | melanophilin | 0.505 | Downregulated |
| 213 | SETDB2 | SET domain bifurcated histone lysine methyltransferase 2 | 0.505 | Downregulated |
| 214 | ZBTB33 | zinc finger and BTB domain containing 33 | 0.506 | Downregulated |
| 215 | UFSP2 | UFM1 specific peptidase 2 | 0.507 | Downregulated |
| 216 | FOXN3 | forkhead box N3 | 0.508 | Downregulated |
| 217 | DMXL1 | Dmx like 1 | 0.508 | Downregulated |
| 218 | PFDN6 | prefoldin subunit 6 | 0.509 | Downregulated |
| 219 | OSER1 | oxidative stress responsive serine rich 1 | 0.511 | Downregulated |
| 220 | FAM126B | family with sequence similarity 126 member B | 0.512 | Downregulated |
| 221 | PLEKHA3 | pleckstrin homology domain containing A3 | 0.512 | Downregulated |
| 222 | PSEN2 | presenilin 2 | 0.513 | Downregulated |
| 223 | ORC5 | origin recognition complex subunit 5 | 0.514 | Downregulated |
| 224 | TULP4 | TUB like protein 4 | 0.515 | Downregulated |
| 225 | KLF12 | Kruppel like factor 12 | 0.517 | Downregulated |
| 226 | PLEKHA4 | pleckstrin homology domain containing A4 | 0.517 | Downregulated |
| 227 | RWDD4 | RWD domain containing 4 | 0.520 | Downregulated |
| 228 | POR | cytochrome p450 oxidoreductase | 0.522 | Downregulated |
| 229 | GPR85 | G protein-coupled receptor 85 | 0.524 | Downregulated |
| 230 | ATP6V1D | ATPase H+ transporting V1 subunit D | 0.525 | Downregulated |
| 231 | NDRG1 | N-myc downstream regulated 1 | 0.525 | Downregulated |
| 232 | STOM | stomatin | 0.525 | Downregulated |
| 233 | NKRF | NFKB repressing factor | 0.525 | Downregulated |
| 234 | ZBTB21 | zinc finger and BTB domain containing 21 | 0.526 | Downregulated |
| 235 | RPPH1 | ribonuclease P RNA component H1 | 0.527 | Downregulated |
| 236 | FAM21C |  | 0.528 | Downregulated |
| 237 | MTO1 | mitochondrial tRNA translation optimization 1 | 0.528 | Downregulated |
| 238 | MBD5 | methyl-CpG binding domain protein 5 | 0.528 | Downregulated |
| 239 | IDS | iduronate 2-sulfatase | 0.528 | Downregulated |
| 240 | HEY2 | hes related family bHLH transcription factor with YRPW motif 2 | 0.529 | Downregulated |
| 241 | KIF21A | kinesin family member 21A | 0.529 | Downregulated |
| 242 | SMPDL3A | sphingomyelin phosphodiesterase acid like 3A | 0.529 | Downregulated |
| 243 | RPS27A | ribosomal protein S27a | 0.532 | Downregulated |
| 244 | FLOT1 | flotillin 1 | 0.532 | Downregulated |
| 245 | C14orf2 |  | 0.532 | Downregulated |
| 246 | C5orf28 |  | 0.534 | Downregulated |
| 247 | NBEA | neurobeachin | 0.534 | Downregulated |
| 248 | TM2D3 | TM2 domain containing 3 | 0.535 | Downregulated |
| 249 | BCL2L11 | BCL2 like 11 | 0.536 | Downregulated |
| 250 | RHEB | Ras homolog, mTORC1 binding | 0.536 | Downregulated |
| 251 | PPP3CC | protein phosphatase 3 catalytic subunit gamma | 0.536 | Downregulated |
| 252 | BVES | blood vessel epicardial substance | 0.539 | Downregulated |
| 253 | MRFAP1L1 | Morf4 family associated protein 1 like 1 | 0.539 | Downregulated |
| 254 | ZFAS1 | ZNFX1 antisense RNA 1 | 0.540 | Downregulated |
| 255 | GAS2 | growth arrest specific 2 | 0.541 | Downregulated |
| 256 | CXCR4 | C-X-C motif chemokine receptor 4 | 0.542 | Downregulated |
| 257 | GLIPR1L2 | GLIPR1 like 2 | 0.542 | Downregulated |
| 258 | ZNF420 | zinc finger protein 420 | 0.543 | Downregulated |
| 259 | SPIRE1 | spire type actin nucleation factor 1 | 0.544 | Downregulated |
| 260 | TP53INP2 | tumor protein p53 inducible nuclear protein 2 | 0.545 | Downregulated |
| 261 | PPM1K | protein phosphatase, Mg2+/Mn2+ dependent 1K | 0.545 | Downregulated |
| 262 | TOM1 | target of myb1 membrane trafficking protein | 0.547 | Downregulated |
| 263 | ATM | ATM serine/threonine kinase | 0.547 | Downregulated |
| 264 | HSPE1 | heat shock protein family E (Hsp10) member 1 | 0.547 | Downregulated |
| 265 | RIPK1 | receptor interacting serine/threonine kinase 1 | 0.548 | Downregulated |
| 266 | ARMCX6 | armadillo repeat containing X-linked 6 | 0.549 | Downregulated |
| 267 | LGALS3 | galectin 3 | 0.549 | Downregulated |
| 268 | ZNF383 | zinc finger protein 383 | 0.551 | Downregulated |
| 269 | TSHZ3 | teashirt zinc finger homeobox 3 | 0.551 | Downregulated |
| 270 | SPG20OS |  | 0.553 | Downregulated |
| 271 | MRPL44 | mitochondrial ribosomal protein L44 | 0.553 | Downregulated |
| 272 | MUC19 | mucin 19, oligomeric | 0.555 | Downregulated |
| 273 | ATRX | ATRX chromatin remodeler | 0.555 | Downregulated |
| 274 | AHSA1 | activator of HSP90 ATPase activity 1 | 0.555 | Downregulated |
| 275 | STMN3 | stathmin 3 | 0.556 | Downregulated |
| 276 | CARF | calcium responsive transcription factor | 0.557 | Downregulated |
| 277 | FAM53B | family with sequence similarity 53 member B | 0.557 | Downregulated |
| 278 | TMEM140 | transmembrane protein 140 | 0.559 | Downregulated |
| 279 | CD63 | CD63 molecule | 0.562 | Downregulated |
| 280 | SLC38A2 | solute carrier family 38 member 2 | 0.564 | Downregulated |
| 281 | RYBP | RING1 and YY1 binding protein | 0.565 | Downregulated |
| 282 | RHEB |  | 0.565 | Downregulated |
| 283 | UPF3A | UPF3A regulator of nonsense mediated mRNA decay | 0.566 | Downregulated |
| 284 | VAT1 | vesicle amine transport 1 | 0.567 | Downregulated |
| 285 | SNORA54 | small nucleolar RNA, H/ACA box 54 | 0.569 | Downregulated |
| 286 | LIN7A | lin-7 homolog A, crumbs cell polarity complex component | 0.571 | Downregulated |
| 287 | TRIP11 | thyroid hormone receptor interactor 11 | 0.572 | Downregulated |
| 288 | TBC1D20 | TBC1 domain family member 20 | 0.573 | Downregulated |
| 289 | DUSP3 | dual specificity phosphatase 3 | 0.573 | Downregulated |
| 290 | MXRA7 | matrix remodeling associated 7 | 0.576 | Downregulated |
| 291 | RPL24 | ribosomal protein L24 | 0.578 | Downregulated |
| 292 | SLC12A7 | solute carrier family 12 member 7 | 0.578 | Downregulated |
| 293 | MAP3K7CL | MAP3K7 C-terminal like | 0.580 | Downregulated |
| 294 | RSRP1 | arginine and serine rich protein 1 | 0.583 | Downregulated |
| 295 | TAF7 | TATA-box binding protein associated factor 7 | 0.588 | Downregulated |
| 296 | RDH11 | retinol dehydrogenase 11 | 0.590 | Downregulated |
| 297 | HIAT1 |  | 0.590 | Downregulated |
| 298 | ZNF398 | zinc finger protein 398 | 0.591 | Downregulated |
| 299 | TOR1AIP1 | torsin 1A interacting protein 1 | 0.591 | Downregulated |
| 300 | SCARNA10 | small Cajal body-specific RNA 10 | 0.591 | Downregulated |
| 301 | LOC100505549 |  | 0.592 | Downregulated |
| 302 | ST6GALNAC3 | ST6 N-acetylgalactosaminide alpha-2,6-sialyltransferase 3 | 0.593 | Downregulated |
| 303 | SLC3A2 | solute carrier family 3 member 2 | 0.593 | Downregulated |
| 304 | ZC3H8 | zinc finger CCCH-type containing 8 | 0.594 | Downregulated |
| 305 | PRKAR1A | protein kinase cAMP-dependent type I regulatory subunit alpha | 0.594 | Downregulated |
| 306 | RNF24 | ring finger protein 24 | 0.594 | Downregulated |
| 307 | CLCN7 | chloride voltage-gated channel 7 | 0.595 | Downregulated |
| 308 | RAB5B | RAB5B, member RAS oncogene family | 0.595 | Downregulated |
| 309 | SPTBN2 | spectrin beta, non-erythrocytic 2 | 0.597 | Downregulated |
| 310 | SPATS2 | spermatogenesis associated serine rich 2 | 0.598 | Downregulated |
| 311 | SOBP | sine oculis binding protein homolog | 0.599 | Downregulated |
| 312 | ZNF543 | zinc finger protein 543 | 0.599 | Downregulated |
| 313 | STAM | signal transducing adaptor molecule | 0.600 | Downregulated |
| 314 | MRPL18 | mitochondrial ribosomal protein L18 | 0.601 | Downregulated |
| 315 | ZNF322 |  | 0.602 | Downregulated |
| 316 | UBE2D3 | ubiquitin conjugating enzyme E2 D3 | 0.603 | Downregulated |
| 317 | BCL7A | BAF chromatin remodeling complex subunit BCL7A | 0.604 | Downregulated |
| 318 | RXRG | retinoid X receptor gamma | 0.606 | Downregulated |
| 319 | METTL23 | methyltransferase like 23 | 0.607 | Downregulated |
| 320 | ISM1 | isthmin 1 | 0.610 | Downregulated |
| 321 | ESCO1 | establishment of sister chromatid cohesion N-acetyltransferase 1 | 0.611 | Downregulated |
| 322 | LRRC75A-AS1 |  | 0.612 | Downregulated |
| 323 | RNF13 | ring finger protein 13 | 0.612 | Downregulated |
| 324 | KIDINS220 | kinase D interacting substrate 220 | 0.613 | Downregulated |
| 325 | TPP1 | tripeptidyl peptidase 1 | 0.614 | Downregulated |
| 326 | RUNX3 | RUNX family transcription factor 3 | 0.614 | Downregulated |
| 327 | SCARNA12 | small Cajal body-specific RNA 12 | 0.614 | Downregulated |
| 328 | CTNNA1 | catenin alpha 1 | 0.614 | Downregulated |
| 329 | ACOX1 | acyl-CoA oxidase 1 | 0.615 | Downregulated |
| 330 | PPP1R13B | protein phosphatase 1 regulatory subunit 13B | 0.617 | Downregulated |
| 331 | PHLPP1 | PH domain and leucine rich repeat protein phosphatase 1 | 0.617 | Downregulated |
| 332 | TGFBR3 | transforming growth factor beta receptor 3 | 0.617 | Downregulated |
| 333 | MAMLD1 | mastermind like domain containing 1 | 0.619 | Downregulated |
| 334 | TP53INP1 | tumor protein p53 inducible nuclear protein 1 | 0.620 | Downregulated |
| 335 | RPS6KA2 | ribosomal protein S6 kinase A2 | 0.621 | Downregulated |
| 336 | GSPT2 | G1 to S phase transition 2 | 0.621 | Downregulated |
| 337 | NFIX | nuclear factor I X | 0.621 | Downregulated |
| 338 | SMAD2 | SMAD family member 2 | 0.624 | Downregulated |
| 339 | DIP2C | disco interacting protein 2 homolog C | 0.625 | Downregulated |
| 340 | MED13L | mediator complex subunit 13L | 0.625 | Downregulated |
| 341 | HELQ | helicase, POLQ like | 0.625 | Downregulated |
| 342 | SLC39A6 | solute carrier family 39 member 6 | 0.625 | Downregulated |
| 343 | MRPS22 | mitochondrial ribosomal protein S22 | 0.626 | Downregulated |
| 344 | FAM35A |  | 0.629 | Downregulated |
| 345 | SNRPF | small nuclear ribonucleoprotein polypeptide F | 0.629 | Downregulated |
| 346 | SLC35A1 | solute carrier family 35 member A1 | 0.633 | Downregulated |
| 347 | PTP4A1 | protein tyrosine phosphatase 4A1 | 0.634 | Downregulated |
| 348 | MICA | MHC class I polypeptide-related sequence A | 0.634 | Downregulated |
| 349 | SCN8A | sodium voltage-gated channel alpha subunit 8 | 0.634 | Downregulated |
| 350 | NEK9 | NIMA related kinase 9 | 0.634 | Downregulated |
| 351 | OFD1 | OFD1 centriole and centriolar satellite protein | 0.639 | Downregulated |
| 352 | RPS19BP1 | ribosomal protein S19 binding protein 1 | 0.639 | Downregulated |
| 353 | MRPL57 | mitochondrial ribosomal protein L57 | 0.640 | Downregulated |
| 354 | TSN | translin | 0.641 | Downregulated |
| 355 | GDPD5 | glycerophosphodiester phosphodiesterase domain containing 5 | 0.641 | Downregulated |
| 356 | IGF1R |  | 0.643 | Downregulated |
| 357 | IRF2 | interferon regulatory factor 2 | 0.643 | Downregulated |
| 358 | SNORD105 | small nucleolar RNA, C/D box 105 | 0.643 | Downregulated |
| 359 | ZFY | zinc finger protein Y-linked | 0.644 | Downregulated |
| 360 | RPL21P28 | ribosomal protein L21 pseudogene 28 | 0.645 | Downregulated |
| 361 | R3HCC1L | R3H domain and coiled-coil containing 1 like | 0.645 | Downregulated |
| 362 | ZBTB24 | zinc finger and BTB domain containing 24 | 0.646 | Downregulated |
| 363 | LOC646358 |  | 0.649 | Downregulated |
| 364 | MYCBP2 | MYC binding protein 2 | 0.652 | Downregulated |
| 365 | DUSP6 | dual specificity phosphatase 6 | 0.652 | Downregulated |
| 366 | PCDH9 | protocadherin 9 | 0.652 | Downregulated |
| 367 | DCTN6 | dynactin subunit 6 | 0.653 | Downregulated |
| 368 | ARFGEF2 | ADP ribosylation factor guanine nucleotide exchange factor 2 | 0.655 | Downregulated |
| 369 | PDCD10 | programmed cell death 10 | 0.655 | Downregulated |
| 370 | PIGT | phosphatidylinositol glycan anchor biosynthesis class T | 0.656 | Downregulated |
| 371 | BCL2 | BCL2 apoptosis regulator | 0.656 | Downregulated |
| 372 | DNAAF2 | dynein axonemal assembly factor 2 | 0.656 | Downregulated |
| 373 | SORT1 | sortilin 1 | 0.658 | Downregulated |
| 374 | SNORA49 | small nucleolar RNA, H/ACA box 49 | 0.660 | Downregulated |
| 375 | BBS12 | Bardet-Biedl syndrome 12 | 0.661 | Downregulated |
| 376 | ZNF571 | zinc finger protein 571 | 0.662 | Downregulated |
| 377 | GTF3C3 | general transcription factor IIIC subunit 3 | 0.662 | Downregulated |
| 378 | AP5M1 | adaptor related protein complex 5 subunit mu 1 | 0.664 | Downregulated |
| 379 | KIAA0825 | KIAA0825 | 0.666 | Downregulated |
| 380 | SETD9 | SET domain containing 9 | 0.666 | Downregulated |
| 381 | DYNLT3 | dynein light chain Tctex-type 3 | 0.669 | Downregulated |
| 382 | WDR48 | WD repeat domain 48 | 0.669 | Downregulated |
| 383 | PHC3 | polyhomeotic homolog 3 | 0.670 | Downregulated |
| 384 | PLD3 | phospholipase D family member 3 | 0.673 | Downregulated |
| 385 | HS2ST1 | heparan sulfate 2-O-sulfotransferase 1 | 0.673 | Downregulated |
| 386 | C5orf51 | chromosome 5 open reading frame 51 | 0.674 | Downregulated |
| 387 | LRRC37A3 | leucine rich repeat containing 37 member A3 | 0.675 | Downregulated |
| 388 | SLC2A14 | solute carrier family 2 member 14 | 0.677 | Downregulated |
| 389 | CNNM3 | cyclin and CBS domain divalent metal cation transport mediator 3 | 0.677 | Downregulated |
| 390 | GSTO1 | glutathione S-transferase omega 1 | 0.678 | Downregulated |
| 391 | USF2 | upstream transcription factor 2, c-fos interacting | 0.679 | Downregulated |
| 392 | RPL21P28 |  | 0.682 | Downregulated |
| 393 | PSTPIP2 | proline-serine-threonine phosphatase interacting protein 2 | 0.686 | Downregulated |
| 394 | YBX3 | Y-box binding protein 3 | 0.688 | Downregulated |
| 395 | TSHZ1 | teashirt zinc finger homeobox 1 | 0.694 | Downregulated |
| 396 | SPATA13 | spermatogenesis associated 13 | 0.694 | Downregulated |
| 397 | NCOA4 | nuclear receptor coactivator 4 | 0.694 | Downregulated |
| 398 | FNDC4 | fibronectin type III domain containing 4 | 0.697 | Downregulated |
| 399 | LOC339803 |  | 0.698 | Downregulated |
| 400 | RICTOR | RPTOR independent companion of MTOR complex 2 | 0.701 | Downregulated |
| 401 | CCDC47 | coiled-coil domain containing 47 | 0.701 | Downregulated |
| 402 | PSMF1 | proteasome inhibitor subunit 1 | 0.704 | Downregulated |
| 403 | PAN2 | poly(A) specific ribonuclease subunit PAN2 | 0.704 | Downregulated |
| 404 | FAM43A | family with sequence similarity 43 member A | 0.710 | Downregulated |
| 405 | ZFHX4 | zinc finger homeobox 4 | 0.711 | Downregulated |
| 406 | DHCR24 | 24-dehydrocholesterol reductase | 0.711 | Downregulated |
| 407 | TRMT12 | tRNA methyltransferase 12 homolog | 0.714 | Downregulated |
| 408 | CPNE8 | copine 8 | 0.717 | Downregulated |
| 409 | ARHGAP35 | Rho GTPase activating protein 35 | 0.721 | Downregulated |
| 410 | EVI2A | ecotropic viral integration site 2A | 0.721 | Downregulated |
| 411 | ATP1A1 | ATPase Na+/K+ transporting subunit alpha 1 | 0.724 | Downregulated |
| 412 | TSKU | tsukushi, small leucine rich proteoglycan | 0.724 | Downregulated |
| 413 | SCARNA17 |  | 0.724 | Downregulated |
| 414 | MED7 | mediator complex subunit 7 | 0.725 | Downregulated |
| 415 | LYRM2 | LYR motif containing 2 | 0.727 | Downregulated |
| 416 | PINK1 | PTEN induced kinase 1 | 0.728 | Downregulated |
| 417 | CEP19 | centrosomal protein 19 | 0.729 | Downregulated |
| 418 | BMPR1A | bone morphogenetic protein receptor type 1A | 0.731 | Downregulated |
| 419 | ZNF568 |  | 0.734 | Downregulated |
| 420 | DYNC1H1 |  | 0.737 | Downregulated |
| 421 | SAT2 | spermidine/spermine N1-acetyltransferase family member 2 | 0.737 | Downregulated |
| 422 | F8 | coagulation factor VIII | 0.738 | Downregulated |
| 423 | C18orf21 | chromosome 18 open reading frame 21 | 0.739 | Downregulated |
| 424 | RSRC2 | arginine and serine rich coiled-coil 2 | 0.739 | Downregulated |
| 425 | COLEC12 | collectin subfamily member 12 | 0.739 | Downregulated |
| 426 | EMD | emerin | 0.741 | Downregulated |
| 427 | ZNF146 | zinc finger protein 146 | 0.742 | Downregulated |
| 428 | COQ10A | coenzyme Q10A | 0.744 | Downregulated |
| 429 | GPNMB | glycoprotein nmb | 0.745 | Downregulated |
| 430 | ATP6V1B2 |  | 0.747 | Downregulated |
| 431 | CREBL2 | cAMP responsive element binding protein like 2 | 0.748 | Downregulated |
| 432 | DIP2B | disco interacting protein 2 homolog B | 0.752 | Downregulated |
| 433 | GRK5 | G protein-coupled receptor kinase 5 | 0.753 | Downregulated |
| 434 | ZNF429 | zinc finger protein 429 | 0.755 | Downregulated |
| 435 | FOXN2 | forkhead box N2 | 0.755 | Downregulated |
| 436 | AFG3L2 | AFG3 like matrix AAA peptidase subunit 2 | 0.756 | Downregulated |
| 437 | PTBP2 | polypyrimidine tract binding protein 2 | 0.760 | Downregulated |
| 438 | PRMT6 | protein arginine methyltransferase 6 | 0.763 | Downregulated |
| 439 | PIM1 | Pim-1 proto-oncogene, serine/threonine kinase | 0.765 | Downregulated |
| 440 | FHL1 | four and a half LIM domains 1 | 0.767 | Downregulated |
| 441 | ZNF570 | zinc finger protein 570 | 0.769 | Downregulated |
| 442 | MRPS10 | mitochondrial ribosomal protein S10 | 0.773 | Downregulated |
| 443 | RAI1 | retinoic acid induced 1 | 0.776 | Downregulated |
| 444 | GATA6 | GATA binding protein 6 | 0.777 | Downregulated |
| 445 | PROX1 | prospero homeobox 1 | 0.777 | Downregulated |
| 446 | KIAA1468 |  | 0.780 | Downregulated |
| 447 | PAQR5 | progestin and adipoQ receptor family member 5 | 0.781 | Downregulated |
| 448 | PDE8A | phosphodiesterase 8A | 0.783 | Downregulated |
| 449 | NUDT14 | nudix hydrolase 14 | 0.783 | Downregulated |
| 450 | CSPG4 | chondroitin sulfate proteoglycan 4 | 0.784 | Downregulated |
| 451 | UPF3A |  | 0.785 | Downregulated |
| 452 | SARS |  | 0.790 | Downregulated |
| 453 | MMP16 | matrix metallopeptidase 16 | 0.790 | Downregulated |
| 454 | CLN8 | CLN8 transmembrane ER and ERGIC protein | 0.793 | Downregulated |
| 455 | MSRB3 | methionine sulfoxide reductase B3 | 0.794 | Downregulated |
| 456 | PICK1 | protein interacting with PRKCA 1 | 0.798 | Downregulated |
| 457 | MAEA | macrophage erythroblast attacher | 0.800 | Downregulated |
| 458 | AVPI1 | arginine vasopressin induced 1 | 0.801 | Downregulated |
| 459 | SFT2D1 | SFT2 domain containing 1 | 0.804 | Downregulated |
| 460 | S100A6 | S100 calcium binding protein A6 | 0.806 | Downregulated |
| 461 | REPS1 | RALBP1 associated Eps domain containing 1 | 0.809 | Downregulated |
| 462 | HOXC9 | homeobox C9 | 0.811 | Downregulated |
| 463 | VIT | vitrin | 0.813 | Downregulated |
| 464 | PNLDC1 | PARN like, ribonuclease domain containing 1 | 0.813 | Downregulated |
| 465 | KCNJ2 | potassium inwardly rectifying channel subfamily J member 2 | 0.820 | Downregulated |
| 466 | SEMA4C | semaphorin 4C | 0.821 | Downregulated |
| 467 | RSU1 | Ras suppressor protein 1 | 0.821 | Downregulated |
| 468 | ARPC1A | actin related protein 2/3 complex subunit 1A | 0.826 | Downregulated |
| 469 | HOXB2 | homeobox B2 | 0.832 | Downregulated |
| 470 | MGST1 | microsomal glutathione S-transferase 1 | 0.837 | Downregulated |
| 471 | ZEB2 | zinc finger E-box binding homeobox 2 | 0.840 | Downregulated |
| 472 | ICE1 | interactor of little elongation complex ELL subunit 1 | 0.841 | Downregulated |
| 473 | POSTN | periostin | 0.844 | Downregulated |
| 474 | IL6ST | interleukin 6 signal transducer | 0.847 | Downregulated |
| 475 | CD24 | CD24 molecule | 0.853 | Downregulated |
| 476 | ZNF563 | zinc finger protein 563 | 0.856 | Downregulated |
| 477 | ARHGAP5 | Rho GTPase activating protein 5 | 0.869 | Downregulated |
| 478 | KIAA1644 |  | 0.872 | Downregulated |
| 479 | DOK5 | docking protein 5 | 0.880 | Downregulated |
| 480 | MEGF9 | multiple EGF like domains 9 | 0.880 | Downregulated |
| 481 | SIPA1L2 | signal induced proliferation associated 1 like 2 | 0.887 | Downregulated |
| 482 | MAGEC2 | MAGE family member C2 | 0.889 | Downregulated |
| 483 | SGK1 | serum/glucocorticoid regulated kinase 1 | 0.899 | Downregulated |
| 484 | FOXP1 | forkhead box P1 | 0.909 | Downregulated |
| 485 | P4HA1 | prolyl 4-hydroxylase subunit alpha 1 | 0.909 | Downregulated |
| 486 | GOT1 | glutamic-oxaloacetic transaminase 1 | 0.937 | Downregulated |
| 487 | EIF1AY | eukaryotic translation initiation factor 1A Y-linked | 0.977 | Downregulated |
