# Supplementary material for: Gene Expression Signature of BRAF Inhibitor Resistant Melanoma Spheroids
Source: Pathol Oncol Res. 2020 Jul 1;26(4):2557–66. doi: 10.1007/s12253-020-00837-9 (PMC7471197; doi:10.1007/s12253-020-00837-9)
Supplement: Supplementary file 3 — (DOCX 29 kb) [file 12253_2020_837_MOESM3_ESM.docx]

**Supplementary Table 3**

Significantly upregulated genes in sensitive melanoma spheroid compare to sensitive monolayer grouped by molecular pathways

| Pathway identifier | Pathway name | P-Value | FDR Value | Genes included (at least 5) |
| --- | --- | --- | --- | --- |
| R-HSA-68877 | Mitotic Prometaphase | 1.11E-16 | 2.03E-14 | ERCC6L, NUP107, CDCA5, CDCA8, NCAPG, CCP110, SMC3, CENPA, SMC4, AURKB, SKA1, NCAPH, SMC2, CDC20, CCNB2, CCNB1, SGOL2, SGOL1, NUF2, KNTC1, NEK2, SPDL1, CEP78, PLK4, CENPU, CEP135, PLK1, SMC1A, NDC80, ZWINT, HAUS1, CENPE, NEDD1, CENPF, KIF18A, CENPI, CENPJ, CDK1, NCAPD2, KIF2C, CEP41, SPC24, ITGB3BP, SPC25 |
| R-HSA-69206 | G1/S Transition | 1.11E-16 | 2.03E-14 | PCNA, MCM7, RPN2, PRIM1, GMNN, MCM10, TYMS, CCNB1, CDC45, ORC1, ORC2, TK1, FBXO5, RRM2, CDC7, CDC6, CDC25A, CCNA2, WEE1, POLA2, TFDP1, CCNE2, CCNE1, POLE2, CDK1, MCM4, PSME2, MCM5, MNAT1, MCM2 |
| R-HSA-69278 | Cell Cycle, Mitotic | 1.11E-16 | 2.03E-14 | ERCC6L, NUP107, GMNN, CCP110, SMC3, SMC4, SMC2, CDC20, KNTC1, NEK2, FBXO5, GTSE1, BORA, CEP135, HIST1H2AK, ESCO2, CDC25C, CDC25A, CCNE2, CCNE1, PSME2, KIF20A, DNA2, HIST1H2BB, TP53, RPN2, CDCA5, NCAPG, CDCA8, PKMYT1, NCAPH, SKA1, CCNB2, CCNB1, ORC1, ORC2, CEP78, LYN, PLK4, PLK1, CDC7, CDC6, NDC80, ZWINT, TPX2, POLA2, KIF18A, CDK1, MNAT1, MCM7, NCAPG2, MCM10, FOXM1, SGOL2, SGOL1, NUF2, MYBL2, TK1, SPDL1, RFC5, NDC1, KIF23, MASTL, SMC1A, HAUS1, CCNA2, TFDP1, ESPL1, MCM4, KIF2C, MCM5, ITGB3BP, MCM2, HIST1H2BM, PCNA, PRIM1, TYMS, CENPA, AURKB, AURKA, CDC45, CENPU, GINS2, RRM2, GINS3, GINS4, NEDD1, CENPE, WEE1, CENPF, CENPI, POLE2, CENPJ, NCAPD2, NCAPD3, CEP41, SPC24, SPC25 |
| R-HSA-69620 | Cell Cycle Checkpoints | 1.11E-16 | 2.03E-14 | ERCC6L, NUP107, MCM7, MCM10, CDC20, SGOL2, SGOL1, EXO1, NUF2, KNTC1, NBN, TOPBP1, SPDL1, GTSE1, RFC5, RMI1, CDC25C, CDC25A, CCNA2, CCNE2, CCNE1, MCM4, PSME2, KIF2C, MCM5, DNA2, HIST1H2BB, TP53, ITGB3BP, RHNO1, MCM2, HIST1H2BM, RPN2, CDCA8, PKMYT1, CENPA, SKA1, AURKB, CCNB2, BRIP1, CCNB1, CDC45, ORC1, PCBP4, ORC2, CLSPN, BARD1, CENPU, PLK1, MRE11A, CDC7, CDC6, NDC80, ZWINT, CENPE, KIF18A, WEE1, CENPF, CENPI, MDM2, CDK1, SPC24, SPC25 |
| R-HSA-1640170 | Cell Cycle | 1.11E-16 | 2.03E-14 | ERCC6L, NUP107, GMNN, HJURP, CCP110, SMC3, SMC4, SMC2, CDC20, SCP2, RUVBL2, KNTC1, NEK2, FBXO5, NBN, GTSE1, BORA, CEP135, HIST1H2AK, ESCO2, CDC25C, CDC25A, CCNE2, CCNE1, PSME2, KIF20A, DNA2, HIST1H2BB, TP53, RHNO1, RPN2, CDCA5, NCAPG, CDCA8, PKMYT1, NCAPH, SKA1, CCNB2, CCNB1, BRIP1, ORC1, PCBP4, ORC2, CLSPN, CEP78, LYN, BARD1, PLK4, PLK1, CDC7, CDC6, NDC80, ZWINT, TPX2, POLA2, KIF18A, MDM2, CDK1, MNAT1, MCM7, NCAPG2, MCM10, BRCA2, FOXM1, SGOL2, SGOL1, EXO1, MIS18BP1, NUF2, MYBL2, TK1, TOPBP1, SPDL1, RFC5, NDC1, RMI1, KIF23, MASTL, SMC1A, HAUS1, CCNA2, TFDP1, ESPL1, MCM4, KIF2C, MCM5, ITGB3BP, MCM2, HIST1H2BM, PCNA, PRIM1, TYMS, CENPA, AURKB, AURKA, PSMC3IP, CDC45, CENPU, GINS2, RRM2, GINS3, GINS4, MRE11A, NEDD1, CENPE, WEE1, CENPF, RAD51, MIS18A, CENPI, POLE2, CENPJ, NCAPD2, NCAPD3, CEP41, SPC24, SPC25 |
| R-HSA-453279 | Mitotic G1-G1/S phases | 1.11E-16 | 2.03E-14 | PCNA, MCM7, RPN2, PRIM1, GMNN, MCM10, TYMS, CCNB1, CDC45, ORC1, ORC2, MYBL2, TK1, FBXO5, LYN, RRM2, CDC7, CDC6, CDC25A, CCNA2, WEE1, POLA2, TFDP1, CCNE2, CCNE1, POLE2, CDK1, MCM4, PSME2, MCM5, MNAT1, MCM2 |
| R-HSA-69205 | G1/S-Specific Transcription | 4.44E-16 | 6.93E-14 | RRM2, PCNA, TFDP1, CDC45, ORC1, CCNE1, CDK1, TK1, CDC6, FBXO5, TYMS, CDC25A |
| R-HSA-68886 | M Phase | 1.78E-15 | 2.43E-13 | ERCC6L, NUP107, NCAPG2, CCP110, SMC3, SMC4, SMC2, CDC20, SGOL2, SGOL1, NUF2, KNTC1, NEK2, SPDL1, FBXO5, NDC1, CEP135, HIST1H2AK, KIF23, MASTL, SMC1A, HAUS1, ESPL1, PSME2, KIF2C, KIF20A, HIST1H2BB, ITGB3BP, HIST1H2BM, RPN2, CDCA5, NCAPG, CDCA8, CENPA, SKA1, NCAPH, AURKB, CCNB2, CCNB1, CEP78, PLK4, CENPU, PLK1, NDC80, ZWINT, NEDD1, CENPE, KIF18A, CENPF, CENPI, CENPJ, CDK1, NCAPD2, NCAPD3, CEP41, SPC24, SPC25 |
| R-HSA-69481 | G2/M Checkpoints | 8.10E-15 | 9.89E-13 | HIST1H2BM, MCM7, RPN2, MCM10, PKMYT1, CCNB2, BRIP1, CCNB1, CDC45, ORC1, EXO1, ORC2, NBN, TOPBP1, CLSPN, GTSE1, BARD1, RFC5, RMI1, MRE11A, CDC7, CDC6, CDC25C, CDC25A, WEE1, CDK1, MCM4, PSME2, MCM5, DNA2, HIST1H2BB, TP53, RHNO1, MCM2 |
| R-HSA-156711 | Polo-like kinase mediated events | 6.64E-13 | 7.23E-11 | CCNB2, WEE1, CCNB1, CENPF, PLK1, MYBL2, FOXM1, CDC25C, PKMYT1, CDC25A |
| R-HSA-2500257 | Resolution of Sister Chromatid Cohesion | 7.86E-13 | 7.78E-11 | ERCC6L, NUP107, CDCA5, CDCA8, SMC3, CENPA, AURKB, SKA1, CDC20, CCNB2, CCNB1, SGOL2, SGOL1, NUF2, KNTC1, SPDL1, CENPU, PLK1, SMC1A, NDC80, ZWINT, CENPE, CENPF, KIF18A, CENPI, CDK1, KIF2C, SPC24, ITGB3BP, SPC25 |
| R-HSA-141424 | Amplification of signal from the kinetochores | 7.26E-12 | 6.10E-10 | CENPU, ERCC6L, NUP107, PLK1, CDCA8, CENPA, AURKB, NDC80, SKA1, ZWINT, CDC20, CENPE, SGOL2, CENPF, KIF18A, SGOL1, CENPI, NUF2, KNTC1, KIF2C, SPDL1, ITGB3BP, SPC24, SPC25 |
| R-HSA-141444 | Amplification of signal from unattached kinetochores via a MAD2 inhibitory signal | 7.26E-12 | 6.10E-10 | CENPU, ERCC6L, NUP107, PLK1, CDCA8, CENPA, AURKB, NDC80, SKA1, ZWINT, CDC20, CENPE, SGOL2, CENPF, KIF18A, SGOL1, CENPI, NUF2, KNTC1, KIF2C, SPDL1, ITGB3BP, SPC24, SPC25 |
| R-HSA-69618 | Mitotic Spindle Checkpoint | 1.86E-10 | 1.45E-08 | CENPU, ERCC6L, NUP107, PLK1, CDCA8, CENPA, AURKB, NDC80, SKA1, ZWINT, CDC20, CENPE, SGOL2, CENPF, KIF18A, SGOL1, CENPI, NUF2, KNTC1, KIF2C, SPDL1, ITGB3BP, SPC24, SPC25 |
| R-HSA-6791312 | TP53 Regulates Transcription of Cell Cycle Genes | 5.17E-10 | 3.78E-08 | PCNA, CDC25C, AURKA, CCNA2, CCNB1, TFDP1, CCNE2, PCBP4, CCNE1, PLAGL1, CENPJ, CDK1, TP53, E2F7, E2F8 |
| R-HSA-1538133 | G0 and Early G1 | 6.55E-10 | 4.46E-08 | CCNA2, PCNA, TFDP1, CCNE2, CCNE1, CDK1, MYBL2, CDC6, CDC25A |
| R-HSA-69242 | S Phase | 8.98E-10 | 5.75E-08 | PCNA, MCM7, RPN2, CDCA5, PRIM1, SMC3, CDC45, ORC1, ORC2, RFC5, GINS2, GINS3, GINS4, CDC6, ESCO2, SMC1A, CDC25A, CCNA2, WEE1, POLA2, TFDP1, CCNE2, CCNE1, POLE2, MCM4, PSME2, MCM5, DNA2, MNAT1, MCM2 |
| R-HSA-69275 | G2/M Transition | 9.54E-10 | 5.82E-08 | RPN2, CCP110, FOXM1, PKMYT1, AURKA, CCNB2, CCNB1, MYBL2, NEK2, GTSE1, CEP78, PLK4, BORA, CEP135, PLK1, CDC25C, CDC25A, HAUS1, CCNA2, TPX2, NEDD1, CENPF, WEE1, CENPJ, CDK1, PSME2, CEP41, MNAT1, TP53 |
| R-HSA-453274 | Mitotic G2-G2/M phases | 1.20E-09 | 6.86E-08 | RPN2, CCP110, FOXM1, PKMYT1, AURKA, CCNB2, CCNB1, MYBL2, NEK2, GTSE1, CEP78, PLK4, BORA, CEP135, PLK1, CDC25C, CDC25A, HAUS1, CCNA2, TPX2, NEDD1, CENPF, WEE1, CENPJ, CDK1, PSME2, CEP41, MNAT1, TP53 |
| R-HSA-68962 | Activation of the pre-replicative complex | 2.92E-09 | 1.52E-07 | MCM7, PRIM1, GMNN, MCM10, CDC7, CDC6, POLA2, CDC45, ORC1, POLE2, ORC2, MCM4, MCM5, MCM2 |
| R-HSA-5693538 | Homology Directed Repair | 5.13E-09 | 2.51E-07 | BARD1, RFC5, HIST1H2BM, PCNA, RMI1, XRCC2, MRE11A, BRCA2, RAD51AP1, CCNA2, BRIP1, RAD51, EXO1, POLE2, TIMELESS, TOPBP1, NBN, DNA2, CLSPN, HIST1H2BB, POLH, RHNO1 |
| R-HSA-2467813 | Separation of Sister Chromatids | 5.71E-09 | 2.68E-07 | ERCC6L, NUP107, RPN2, CDCA5, CDCA8, SMC3, CENPA, AURKB, SKA1, CDC20, SGOL2, SGOL1, NUF2, KNTC1, SPDL1, CENPU, PLK1, SMC1A, NDC80, ZWINT, CENPE, CENPF, KIF18A, ESPL1, CENPI, PSME2, KIF2C, SPC24, ITGB3BP, SPC25 |
| R-HSA-176187 | Activation of ATR in response to replication stress | 7.88E-09 | 3.55E-07 | RFC5, MCM7, MCM10, CDC7, CDC6, CDC25C, CDC25A, CDC45, ORC1, ORC2, MCM4, MCM5, CLSPN, MCM2 |
| R-HSA-5693567 | HDR through Homologous Recombination (HRR) or Single Strand Annealing (SSA) | 9.19E-09 | 3.95E-07 | BARD1, RFC5, HIST1H2BM, PCNA, RMI1, XRCC2, MRE11A, BRCA2, RAD51AP1, CCNA2, BRIP1, RAD51, EXO1, POLE2, TIMELESS, TOPBP1, NBN, DNA2, CLSPN, HIST1H2BB, POLH, RHNO1 |
| R-HSA-2555396 | Mitotic Metaphase and Anaphase | 1.09E-08 | 4.57E-07 | ERCC6L, NUP107, RPN2, CDCA5, CDCA8, SMC3, CENPA, AURKB, SKA1, CDC20, SGOL2, SGOL1, NUF2, KNTC1, SPDL1, FBXO5, CENPU, PLK1, SMC1A, NDC80, ZWINT, CENPE, CENPF, KIF18A, ESPL1, CENPI, PSME2, KIF2C, SPC24, ITGB3BP, SPC25 |
| R-HSA-3700989 | Transcriptional Regulation by TP53 | 1.68E-08 | 6.50E-07 | PCNA, AURKB, AURKA, BRIP1, CCNB1, EXO1, CASP6, PCBP4, PLAGL1, NBN, TOPBP1, TAF9B, RFFL, E2F7, PDK1, E2F8, BARD1, RFC5, ADSL, RMI1, MRE11A, CDC25C, LRPPRC, DDB2, CCNA2, MOV10, TPX2, TFDP1, CCNE2, CCNE1, FANCD2, AGO1, CENPJ, MDM2, CDK1, FAS, DNA2, MNAT1, TP53, RHNO1 |
| R-HSA-69473 | G2/M DNA damage checkpoint | 1.71E-08 | 6.50E-07 | BARD1, RFC5, HIST1H2BM, RMI1, MRE11A, CDC25C, BRIP1, WEE1, CCNB1, EXO1, CDK1, TOPBP1, NBN, DNA2, HIST1H2BB, TP53, RHNO1 |
| R-HSA-5663220 | RHO GTPases Activate Formins | 1.76E-08 | 6.50E-07 | ERCC6L, NUP107, CDCA8, CENPA, AURKB, SKA1, CDC20, SGOL2, SGOL1, NUF2, KNTC1, SPDL1, CENPU, PLK1, NDC80, ZWINT, CENPE, CENPF, KIF18A, DIAPH3, CENPI, KIF2C, SPC24, ITGB3BP, SPC25 |
| R-HSA-69306 | DNA Replication | 2.44E-08 | 8.78E-07 | PCNA, MCM7, RPN2, PRIM1, GMNN, MCM10, CDC45, ORC1, ORC2, RFC5, GINS2, GINS3, GINS4, CDC7, CDC6, CCNA2, POLA2, CCNE2, CCNE1, POLE2, MCM4, PSME2, MCM5, DNA2, MCM2 |
| R-HSA-68882 | Mitotic Anaphase | 2.71E-08 | 9.49E-07 | ERCC6L, NUP107, RPN2, CDCA5, CDCA8, SMC3, CENPA, AURKB, SKA1, CDC20, SGOL2, SGOL1, NUF2, KNTC1, SPDL1, CENPU, PLK1, SMC1A, NDC80, ZWINT, CENPE, CENPF, KIF18A, ESPL1, CENPI, PSME2, KIF2C, SPC24, ITGB3BP, SPC25 |
| R-HSA-5693579 | Homologous DNA Pairing and Strand Exchange | 3.46E-08 | 1.18E-06 | BARD1, RFC5, RMI1, XRCC2, MRE11A, BRCA2, RAD51AP1, BRIP1, RAD51, EXO1, TOPBP1, NBN, DNA2, RHNO1 |
| R-HSA-69190 | DNA strand elongation | 4.66E-08 | 1.49E-06 | RFC5, GINS2, PCNA, MCM7, PRIM1, GINS3, GINS4, POLA2, CDC45, MCM4, MCM5, DNA2, MCM2 |
| R-HSA-5685942 | HDR through Homologous Recombination (HRR) | 8.74E-08 | 2.71E-06 | BARD1, RFC5, PCNA, RMI1, XRCC2, MRE11A, BRCA2, RAD51AP1, BRIP1, RAD51, EXO1, POLE2, TOPBP1, NBN, DNA2, POLH, RHNO1 |
| R-HSA-194315 | Signaling by Rho GTPases | 9.82E-08 | 2.95E-06 | ARHGAP11B, HIST1H2BM, ERCC6L, NUP107, ARHGEF26, KIF14, CDCA8, CENPA, IQGAP3, AURKB, SKA1, MYLK, CDC20, SYDE1, SGOL2, SGOL1, RACGAP1, CHN1, ARHGDIB, NUF2, RAC2, KNTC1, SPDL1, ECT2, PDK1, CENPU, HIST1H2AK, PLK1, CDC25C, NDC80, ZWINT, CENPE, CENPF, KIF18A, DEPDC1B, DIAPH3, CENPI, TRIP10, KIF2C, HIST1H2BB, ZNF774, SPC24, ITGB3BP, RAD18, SPC25 |
| R-HSA-5693616 | Presynaptic phase of homologous DNA pairing and strand exchange | 1.10E-07 | 3.20E-06 | BARD1, RFC5, RMI1, XRCC2, MRE11A, BRCA2, BRIP1, RAD51, EXO1, TOPBP1, NBN, DNA2, RHNO1 |
| R-HSA-176974 | Unwinding of DNA | 1.36E-07 | 3.81E-06 | GINS2, CDC45, MCM7, GINS3, GINS4, MCM4, MCM5, MCM2 |
| R-HSA-5693532 | DNA Double-Strand Break Repair | 1.57E-07 | 4.25E-06 | BARD1, RFC5, HIST1H2BM, PCNA, RMI1, XRCC2, MRE11A, BRCA2, RAD51AP1, CCNA2, BRIP1, RAD51, EXO1, POLE2, TIMELESS, TOPBP1, NBN, DNA2, CLSPN, HIST1H2BB, TP53, POLH, RHNO1 |
| R-HSA-5693554 | Resolution of D-loop Structures through Synthesis-Dependent Strand Annealing (SDSA) | 2.54E-07 | 6.61E-06 | RAD51AP1, BARD1, BRIP1, RAD51, EXO1, RMI1, XRCC2, MRE11A, NBN, DNA2, BRCA2 |
| R-HSA-195258 | RHO GTPase Effectors | 3.90E-07 | 1.01E-05 | HIST1H2BM, ERCC6L, NUP107, KIF14, CDCA8, CENPA, IQGAP3, AURKB, SKA1, MYLK, CDC20, SGOL2, SGOL1, NUF2, RAC2, KNTC1, SPDL1, PDK1, CENPU, HIST1H2AK, PLK1, CDC25C, NDC80, ZWINT, CENPE, CENPF, KIF18A, DIAPH3, CENPI, KIF2C, HIST1H2BB, ZNF774, SPC24, ITGB3BP, SPC25 |
| R-HSA-69239 | Synthesis of DNA | 4.64E-07 | 1.11E-05 | RFC5, GINS2, PCNA, MCM7, RPN2, PRIM1, GINS3, GINS4, CDC6, CCNA2, POLA2, CDC45, CCNE2, ORC1, CCNE1, POLE2, ORC2, MCM4, PSME2, MCM5, DNA2, MCM2 |
| R-HSA-69273 | Cyclin A/B1/B2 associated events during G2/M transition | 4.76E-07 | 1.14E-05 | CCNA2, CCNB2, WEE1, CCNB1, PLK1, CDK1, FOXM1, CDC25C, PKMYT1, MNAT1, CDC25A |
| R-HSA-6804116 | TP53 Regulates Transcription of Genes Involved in G1 Cell Cycle Arrest | 5.93E-07 | 1.36E-05 | CCNA2, CCNE2, PCBP4, CCNE1, TP53, E2F7, E2F8 |
| R-HSA-2514853 | Condensation of Prometaphase Chromosomes | 7.19E-07 | 1.65E-05 | CCNB2, CCNB1, CDK1, NCAPG, NCAPD2, SMC4, NCAPH, SMC2 |
| R-HSA-73886 | Chromosome Maintenance | 8.51E-07 | 1.87E-05 | RFC5, CENPU, HIST1H2BM, PCNA, PRIM1, HIST1H2AK, HJURP, CENPA, POLA2, MIS18A, CENPI, MIS18BP1, RUVBL2, POLE2, DNA2, HIST1H2BB, ITGB3BP |
| R-HSA-6804756 | Regulation of TP53 Activity through Phosphorylation | 8.68E-07 | 1.91E-05 | BARD1, RFC5, RMI1, MRE11A, AURKB, AURKA, CCNA2, TPX2, BRIP1, EXO1, MDM2, TOPBP1, NBN, DNA2, TAF9B, TP53, RHNO1 |
| R-HSA-983189 | Kinesins | 1.14E-06 | 2.39E-05 | KIF23, KIF11, KIF22, KIF15, CENPE, KIF18A, KIF18B, RACGAP1, KIFC1, KIF4B, KIF4A, KIF2C, KIF20A, KIF20B |
| R-HSA-5693568 | Resolution of D-loop Structures through Holliday Junction Intermediates | 1.47E-06 | 3.09E-05 | RAD51AP1, BARD1, BRIP1, RAD51, EXO1, RMI1, XRCC2, MRE11A, NBN, DNA2, BRCA2 |
| R-HSA-5693607 | Processing of DNA double-strand break ends | 1.81E-06 | 3.80E-05 | BARD1, RFC5, HIST1H2BM, RMI1, MRE11A, CCNA2, BRIP1, EXO1, TIMELESS, TOPBP1, NBN, DNA2, CLSPN, HIST1H2BB, RHNO1 |
| R-HSA-5693537 | Resolution of D-Loop Structures | 1.91E-06 | 3.82E-05 | RAD51AP1, BARD1, BRIP1, RAD51, EXO1, RMI1, XRCC2, MRE11A, NBN, DNA2, BRCA2 |
| R-HSA-5685938 | HDR through Single Strand Annealing (SSA) | 3.14E-06 | 6.27E-05 | RFC5, BARD1, BRIP1, RAD51, EXO1, RMI1, MRE11A, TOPBP1, NBN, DNA2, RHNO1 |
| R-HSA-69002 | DNA Replication Pre-Initiation | 5.78E-06 | 1.10E-04 | MCM7, RPN2, PRIM1, GMNN, MCM10, CDC7, CDC6, POLA2, CDC45, ORC1, POLE2, ORC2, MCM4, PSME2, MCM5, MCM2 |
| R-HSA-6804114 | TP53 Regulates Transcription of Genes Involved in G2 Cell Cycle Arrest | 8.32E-06 | 1.58E-04 | CCNB1, PCNA, TFDP1, CDK1, CDC25C, TP53, AURKA |
| R-HSA-774815 | Nucleosome assembly | 1.23E-05 | 2.21E-04 | CENPU, HIST1H2BM, MIS18A, CENPI, MIS18BP1, HIST1H2AK, HJURP, HIST1H2BB, CENPA, ITGB3BP |
| R-HSA-606279 | Deposition of new CENPA-containing nucleosomes at the centromere | 1.23E-05 | 2.21E-04 | CENPU, HIST1H2BM, MIS18A, CENPI, MIS18BP1, HIST1H2AK, HJURP, HIST1H2BB, CENPA, ITGB3BP |
| R-HSA-73894 | DNA Repair | 1.41E-05 | 2.54E-04 | HIST1H2BM, PCNA, BRCA2, RAD51AP1, BRIP1, EXO1, NBN, TOPBP1, CLSPN, POLH, BARD1, RFC5, RMI1, MGMT, HIST1H2AK, XRCC2, MRE11A, FANCB, FANCE, DDB2, CCNA2, NEIL3, RAD51, FANCD2, POLE2, UBE2T, TIMELESS, DNA2, HIST1H2BB, MNAT1, TP53, DTL, RAD18, RHNO1 |
| R-HSA-2299718 | Condensation of Prophase Chromosomes | 1.46E-05 | 2.64E-04 | HIST1H2BM, CCNB1, HIST1H2AK, PLK1, NCAPG2, CDK1, NCAPD3, HIST1H2BB, SMC4, SMC2 |
| R-HSA-5633007 | Regulation of TP53 Activity | 1.61E-05 | 2.74E-04 | BARD1, RFC5, RMI1, MRE11A, AURKB, AURKA, CCNA2, TPX2, BRIP1, EXO1, MDM2, CDK1, TOPBP1, NBN, DNA2, TAF9B, RFFL, TP53, PDK1, RHNO1 |
| R-HSA-69478 | G2/M DNA replication checkpoint | 2.28E-05 | 3.88E-04 | CCNB2, WEE1, CCNB1, CDK1, PKMYT1 |
| R-HSA-2565942 | Regulation of PLK1 Activity at G2/M Transition | 3.86E-05 | 6.56E-04 | PLK4, BORA, CEP135, PLK1, CCP110, AURKA, HAUS1, NEDD1, CCNB2, CCNB1, CENPJ, CDK1, NEK2, CEP41, CEP78 |
| R-HSA-176417 | Phosphorylation of Emi1 | 4.29E-05 | 6.86E-04 | CDC20, CCNB1, PLK1, CDK1, FBXO5 |
| R-HSA-6811434 | COPI-dependent Golgi-to-ER retrograde traffic | 5.92E-05 | 9.48E-04 | FKBP11, KIF23, KIF11, KIF22, KIF15, CENPE, KIF18A, KIF18B, RACGAP1, KIFC1, KIF4B, KIF4A, KIF2C, KIF20A, KIF20B |
| R-HSA-8854518 | AURKA Activation by TPX2 | 5.98E-05 | 9.56E-04 | PLK4, CEP135, PLK1, CCP110, AURKA, HAUS1, TPX2, NEDD1, CENPJ, CDK1, NEK2, CEP41, CEP78 |
| R-HSA-8953750 | Transcriptional Regulation by E2F6 | 7.66E-05 | 0.001148877 | RRM2, CBX5, TFDP1, RAD51, CDC7, EZH2 |
| R-HSA-113510 | E2F mediated regulation of DNA replication | 9.30E-05 | 0.001395668 | POLA2, CCNB1, TFDP1, ORC1, PRIM1, ORC2, CDK1 |
| R-HSA-2559586 | DNA Damage/Telomere Stress Induced Senescence | 1.62E-04 | 0.00242273 | CCNA2, HIST1H2BM, CCNE2, CCNE1, HIST1H2AK, MRE11A, NBN, HIST1H2BB, TP53, HIST1H1A |
| R-HSA-2559583 | Cellular Senescence | 2.08E-04 | 0.002915758 | HIST1H2BM, ADSL, CXCL8, HIST1H2AK, MRE11A, CCNA2, MOV10, TFDP1, CCNE2, CCNE1, AGO1, MDM2, NBN, HIST1H2BB, TP53, HIST1H1A, EZH2 |
| R-HSA-157579 | Telomere Maintenance | 3.74E-04 | 0.005232475 | RFC5, HIST1H2BM, POLA2, PCNA, PRIM1, HIST1H2AK, POLE2, RUVBL2, DNA2, HIST1H2BB |
| R-HSA-877300 | Interferon gamma signaling | 3.80E-04 | 0.005324074 | VCAM1, IRF1, TRIM68, TRIM14, HLA-C, GBP4 |
| R-HSA-912446 | Meiotic recombination | 4.76E-04 | 0.006233303 | PSMC3IP, HIST1H2BM, RAD51, HIST1H2AK, MRE11A, NBN, BRCA2, HIST1H2BB |
| R-HSA-1500620 | Meiosis | 4.79E-04 | 0.006233303 | PSMC3IP, HIST1H2BM, RAD51, SCP2, HIST1H2AK, MRE11A, NBN, BRCA2, SMC3, HIST1H2BB, SMC1A |
| R-HSA-380284 | Loss of proteins required for interphase microtubule organization from the centrosome | 6.10E-04 | 0.007935656 | PLK4, NEDD1, CEP135, CENPJ, PLK1, CDK1, CCP110, NEK2, CEP41, HAUS1, CEP78 |
| R-HSA-380259 | Loss of Nlp from mitotic centrosomes | 6.10E-04 | 0.007935656 | PLK4, NEDD1, CEP135, CENPJ, PLK1, CDK1, CCP110, NEK2, CEP41, HAUS1, CEP78 |
| R-HSA-68875 | Mitotic Prophase | 7.54E-04 | 0.009143413 | NDC1, HIST1H2BM, NUP107, HIST1H2AK, PLK1, NCAPG2, MASTL, SMC4, SMC2, CCNB2, CCNB1, CDK1, NCAPD3, HIST1H2BB |
| R-HSA-68884 | Mitotic Telophase/Cytokinesis | 7.62E-04 | 0.009143413 | PLK1, KIF23, KIF20A, SMC3, SMC1A |
| R-HSA-1474165 | Reproduction | 8.22E-04 | 0.009862123 | PSMC3IP, HIST1H2BM, RAD51, SCP2, HIST1H2AK, MRE11A, ADAM9, NBN, BRCA2, SMC3, HIST1H2BB, SMC1A |
| R-HSA-5689880 | Ub-specific processing proteases | 8.54E-04 | 0.010247106 | HIST1H2BM, SMAD3, RPN2, HIST1H2AK, HIST2H2AB, CCP110, IDE, CDC25A, DDB2, SMAD7, CDC20, CCNA2, MDM2, PSME2, CLSPN, TAF9B, HIST1H2BB, TP53 |
| R-HSA-2559580 | Oxidative Stress Induced Senescence | 0.001132461 | 0.013589535 | MOV10, HIST1H2BM, ADSL, TFDP1, HIST1H2AK, AGO1, MDM2, HIST1H2BB, TP53, EZH2 |
| R-HSA-212300 | PRC2 methylates histones and DNA | 0.0012287 | 0.014744395 | HIST1H2BM, DNMT1, HIST1H2AK, MTF2, HIST1H2BB, EZH2 |
| R-HSA-69052 | Switching of origins to a post-replicative state | 0.001522128 | 0.016743408 | CCNA2, CCNE2, ORC1, MCM7, RPN2, CCNE1, ORC2, MCM4, PSME2, MCM5, CDC6, MCM2 |
| R-HSA-68867 | Assembly of the pre-replicative complex | 0.001556724 | 0.017123961 | ORC1, MCM7, RPN2, GMNN, ORC2, MCM4, PSME2, MCM5, CDC6, MCM2 |
| R-HSA-6806003 | Regulation of TP53 Expression and Degradation | 0.001619199 | 0.017811187 | CCNA2, MDM2, CDK1, RFFL, TP53, PDK1 |
| R-HSA-5334118 | DNA methylation | 0.001682228 | 0.018504507 | HIST1H2BM, DNMT1, UHRF1, HIST1H2AK, HIST1H2BB |
| R-HSA-380270 | Recruitment of mitotic centrosome proteins and complexes | 0.001727667 | 0.019004334 | PLK4, NEDD1, CEP135, CENPJ, PLK1, CDK1, CCP110, NEK2, CEP41, HAUS1, CEP78 |
| R-HSA-913531 | Interferon Signaling | 0.001864541 | 0.020509953 | NDC1, VCAM1, IFITM2, NUP107, IRF1, TRIM68, HLA-C, TRIM14, FLNB, KPNA3, GBP4 |
| R-HSA-8856688 | Golgi-to-ER retrograde transport | 0.00190637 | 0.020822717 | FKBP11, KIF23, KIF11, KIF22, KIF15, CENPE, KIF18A, KIF18B, RACGAP1, KIFC1, KIF4B, KIF4A, KIF2C, KIF20A, KIF20B |
| R-HSA-380287 | Centrosome maturation | 0.002082272 | 0.020822717 | PLK4, NEDD1, CEP135, CENPJ, PLK1, CDK1, CCP110, NEK2, CEP41, HAUS1, CEP78 |
| R-HSA-983231 | Factors involved in megakaryocyte development and platelet production | 0.002290389 | 0.022903895 | CBX5, KIF23, KIF11, KIF22, KIF15, CENPE, WEE1, KIF18A, KIF18B, RACGAP1, KIFC1, KIF4B, KIF4A, IRF1, KIF2C, KIF20A, KIF20B, TP53 |
| R-HSA-68949 | Orc1 removal from chromatin | 0.002584517 | 0.025845166 | CCNA2, ORC1, MCM7, RPN2, ORC2, MCM4, PSME2, MCM5, CDC6, MCM2 |
| R-HSA-5620912 | Anchoring of the basal body to the plasma membrane | 0.002753424 | 0.027534243 | PLK4, NEDD1, CEP135, CENPJ, PLK1, CDK1, CCP110, NEK2, RPGRIP1L, CEP41, HAUS1, CEP78 |
| R-HSA-1912408 | Pre-NOTCH Transcription and Translation | 0.003514094 | 0.035140938 | MOV10, HIST1H2BM, ADSL, TFDP1, HIST1H2AK, AGO1, HIST1H2BB, TP53 |
| R-HSA-1236975 | Antigen processing-Cross presentation | 0.003516977 | 0.035169772 | RPN2, ITGB5, PSME2, HLA-C, CTSS |
| R-HSA-453276 | Regulation of mitotic cell cycle | 0.003815275 | 0.035212713 | CDC20, CCNA2, CCNB1, RPN2, PLK1, CDK1, PSME2, NEK2, FBXO5, AURKB, AURKA |
| R-HSA-174143 | APC/C-mediated degradation of cell cycle proteins | 0.003815275 | 0.035212713 | CDC20, CCNA2, CCNB1, RPN2, PLK1, CDK1, PSME2, NEK2, FBXO5, AURKB, AURKA |
| R-HSA-2559585 | Oncogene Induced Senescence | 0.003912524 | 0.035212713 | MOV10, ADSL, TFDP1, AGO1, MDM2, TP53 |
| R-HSA-212436 | Generic Transcription Pathway | 0.004287166 | 0.038584491 | CCNC, ZFP30, EXO1, CASP6, MYBL2, NBN, TOPBP1, PDK1, RFC5, ADSL, ITGA4, RMI1, HIST1H2AK, CDC25C, DDB2, CCNA2, TFDP1, ZNF93, CCNE2, CCNE1, FANCD2, TXNIP, PSME2, DNA2, HIST1H2BB, TP53, RHNO1, HIST1H2BM, PCNA, RPN2, TGFA, AURKB, HDAC7, AURKA, BRIP1, CCNB1, PCBP4, PLAGL1, HIVEP3, ZNF544, TAF9B, RFFL, E2F7, ZNF860, E2F8, ZNF100, ZNF221, BARD1, CBX5, RRM2, SMAD3, MRE11A, CDC7, DEK, LRPPRC, SMAD7, MOV10, TPX2, BMP2, RAD51, ZNF738, AGO1, CENPJ, MDM2, CDK1, FAS, MNAT1, ZNF774, EZH2 |
| R-HSA-6804757 | Regulation of TP53 Degradation | 0.004436396 | 0.039927567 | CCNA2, MDM2, CDK1, RFFL, TP53, PDK1 |
| R-HSA-180786 | Extension of Telomeres | 0.005641046 | 0.050769417 | RFC5, POLA2, PCNA, PRIM1, POLE2, RUVBL2, DNA2 |
| R-HSA-380320 | Recruitment of NuMA to mitotic centrosomes | 0.006545132 | 0.056271787 | PLK4, NEDD1, CEP135, CENPJ, PLK1, CDK1, CCP110, NEK2, CEP41, HAUS1, CEP78 |
| R-HSA-69615 | G1/S DNA Damage Checkpoints | 0.007353103 | 0.058824821 | CCNA2, CCNE2, RPN2, CCNE1, PCBP4, MDM2, PSME2, TP53, CDC25A |
| R-HSA-69186 | Lagging Strand Synthesis | 0.007889216 | 0.063113726 | RFC5, POLA2, PCNA, PRIM1, DNA2 |
| R-HSA-69202 | Cyclin E associated events during G1/S transition | 0.00914971 | 0.073197681 | CCNA2, WEE1, TFDP1, CCNE2, RPN2, CCNE1, PSME2, MNAT1, CDC25A |
| R-HSA-1221632 | Meiotic synapsis | 0.009361856 | 0.074003985 | HIST1H2BM, SCP2, HIST1H2AK, SMC3, HIST1H2BB, SMC1A |
| R-HSA-174417 | Telomere C-strand (Lagging Strand) Synthesis | 0.009403901 | 0.074003985 | RFC5, POLA2, PCNA, PRIM1, POLE2, DNA2 |
| R-HSA-110330 | Recognition and association of DNA glycosylase with site containing an affected purine | 0.009403901 | 0.074003985 | HIST1H2BM, NEIL3, HIST1H2AK, HIST1H2BB |
| R-HSA-5688426 | Deubiquitination | 0.009713624 | 0.074003985 | BARD1, HIST1H2BM, SMAD3, RPN2, HIST1H2AK, HIST2H2AB, CCP110, IDE, CDC25A, DDB2, SMAD7, CDC20, CCNA2, MDM2, CDK1, PSME2, CLSPN, TAF9B, HIST1H2BB, TP53 |
| R-HSA-3214815 | HDACs deacetylate histones | 0.010229846 | 0.074003985 | HIST1H2BM, HIST1H2AK, HIST2H2AB, HIST1H2BB |
| R-HSA-69656 | Cyclin A:Cdk2-associated events at S phase entry | 0.010571998 | 0.074003985 | CCNA2, WEE1, TFDP1, CCNE2, RPN2, CCNE1, PSME2, MNAT1, CDC25A |
| R-HSA-8936459 | RUNX1 regulates genes involved in megakaryocyte differentiation and platelet function | 0.011903446 | 0.083324124 | MOV10, HIST1H2BM, ADSL, HIST1H2AK, AGO1, HIST1H2BB |
| R-HSA-5693565 | Recruitment and ATM-mediated phosphorylation of repair and signaling proteins at DNA double strand breaks | 0.013192354 | 0.092346481 | BARD1, HIST1H2BM, MRE11A, NBN, HIST1H2BB, TP53 |
| R-HSA-110314 | Recognition of DNA damage by PCNA-containing replication complex | 0.013906052 | 0.097342364 | RFC5, PCNA, POLE2, DTL, RAD18 |
| R-HSA-69563 | p53-Dependent G1 DNA Damage Response | 0.018059574 | 0.116124238 | CCNA2, CCNE2, RPN2, CCNE1, PCBP4, MDM2, PSME2, TP53 |
| R-HSA-69580 | p53-Dependent G1/S DNA damage checkpoint | 0.018059574 | 0.116124238 | CCNA2, CCNE2, RPN2, CCNE1, PCBP4, MDM2, PSME2, TP53 |
| R-HSA-1912422 | Pre-NOTCH Expression and Processing | 0.018397006 | 0.116124238 | MOV10, HIST1H2BM, ADSL, TFDP1, HIST1H2AK, AGO1, HIST1H2BB, TP53 |
| R-HSA-5693571 | Nonhomologous End-Joining (NHEJ) | 0.018533529 | 0.116124238 | BARD1, HIST1H2BM, MRE11A, NBN, HIST1H2BB |
| R-HSA-157118 | Signaling by NOTCH | 0.01935404 | 0.116124238 | HIST1H2BM, ADSL, SMAD3, RPN2, HIST1H2AK, CCNC, HDAC7, MOV10, TFDP1, AGO1, TACC3, PSME2, HIST1H2BB, TP53, DLGAP5 |
| R-HSA-73857 | RNA Polymerase II Transcription | 0.021000983 | 0.126005898 | CCNC, SNAPC1, ZFP30, EXO1, CASP6, MYBL2, NBN, TOPBP1, PDK1, RFC5, ADSL, ITGA4, RMI1, HIST1H2AK, CDC25C, DDB2, CCNA2, TFDP1, ZNF93, CCNE2, CCNE1, FANCD2, TXNIP, PSME2, DNA2, HIST1H2BB, TP53, RHNO1, HIST1H2BM, PCNA, RPN2, TGFA, AURKB, HDAC7, AURKA, BRIP1, CCNB1, PCBP4, PLAGL1, HIVEP3, ZNF544, TAF9B, RFFL, E2F7, ZNF860, E2F8, ZNF100, ZNF221, BARD1, CBX5, RRM2, SMAD3, MRE11A, CDC7, DEK, MAGOHB, LRPPRC, SMAD7, MOV10, TPX2, BMP2, RAD51, ZNF738, AGO1, CENPJ, MDM2, CDK1, FAS, MNAT1, ZNF774, EZH2 |
| R-HSA-1566948 | Elastic fibre formation | 0.021840536 | 0.131043218 | EFEMP2, BMP2, ITGB5, FN1, LOXL2 |
| R-HSA-2132295 | MHC class II antigen presentation | 0.023246113 | 0.139476681 | CENPE, KIF18A, RACGAP1, KIF4B, KIF4A, KIF2C, KIF23, KIF20A, KIF11, KIF22, CTSS, KIF15 |
| R-HSA-5693606 | DNA Double Strand Break Response | 0.024096033 | 0.1445762 | BARD1, HIST1H2BM, MRE11A, NBN, HIST1H2BB, TP53 |
| R-HSA-74160 | Gene expression (Transcription) | 0.027226467 | 0.1633588 | NUP107, SUV39H1, CCNC, SNAPC1, ZFP30, EXO1, CASP6, MYBL2, NBN, TOPBP1, PDK1, RFC5, NDC1, ADSL, ITGA4, RMI1, HIST1H2AK, CDC25C, DDB2, CCNA2, TFDP1, ZNF93, CCNE2, CCNE1, FANCD2, MTF2, TXNIP, PSME2, DNA2, HIST1H2BB, TP53, RHNO1, HIST1H2BM, DNMT1, PCNA, RPN2, GTF3C6, UHRF1, TGFA, AURKB, HDAC7, AURKA, BRIP1, CCNB1, PCBP4, PLAGL1, HIVEP3, ZNF544, TAF9B, RFFL, E2F7, ZNF860, E2F8, ZNF100, ZNF221, BARD1, CBX5, RRM2, SMAD3, MRE11A, CDC7, DEK, MAGOHB, LRPPRC, SMAD7, MOV10, TPX2, BMP2, RAD51, ZNF738, AGO1, CENPJ, MDM2, CDK1, FAS, MNAT1, ZNF774, EZH2 |
| R-HSA-176814 | Activation of APC/C and APC/C:Cdc20 mediated degradation of mitotic proteins | 0.027591032 | 0.165546195 | CDC20, CCNA2, CCNB1, RPN2, PLK1, CDK1, PSME2, NEK2 |
| R-HSA-9018519 | Estrogen-dependent gene expression | 0.030552801 | 0.177179824 | MOV10, HIST1H2BM, ADSL, HIST1H2AK, AGO1, TGFA, SMC3, HIST1H2BB, SMC1A |
| R-HSA-4615885 | SUMOylation of DNA replication proteins | 0.03097696 | 0.177179824 | NDC1, PCNA, NUP107, CDCA8, AURKB, AURKA |
| R-HSA-212165 | Epigenetic regulation of gene expression | 0.031815511 | 0.177179824 | HIST1H2BM, DNMT1, UHRF1, HIST1H2AK, SUV39H1, MTF2, DEK, HIST1H2BB, MNAT1, EZH2 |
| R-HSA-3214847 | HATs acetylate histones | 0.035435965 | 0.177179824 | HIST1H2BM, HIST1H2AK, RUVBL2, HIST2H2AB, TAF9B, HIST1H2BB |
| R-HSA-5617833 | Cilium Assembly | 0.036903558 | 0.184517792 | PLK4, TTC26, CEP135, PLK1, CCP110, RPGRIP1L, HSPB11, HAUS1, NEDD1, CENPJ, CDK1, EXOC6, NEK2, CEP41, IFT57, CEP78 |
| R-HSA-5578749 | Transcriptional regulation by small RNAs | 0.037827434 | 0.189137171 | NDC1, HIST1H2BM, NUP107, HIST1H2AK, AGO1, HIST1H2BB |
| R-HSA-194840 | Rho GTPase cycle | 0.039768396 | 0.198841979 | ARHGAP11B, SYDE1, DEPDC1B, ARHGEF26, RACGAP1, CHN1, ARHGDIB, RAC2, TRIP10, ECT2, RAD18 |
| R-HSA-176408 | Regulation of APC/C activators between G1/S and early anaphase | 0.040143439 | 0.200717194 | CDC20, CCNA2, CCNB1, RPN2, PLK1, CDK1, PSME2, FBXO5 |
| R-HSA-73884 | Base Excision Repair | 0.042206992 | 0.211034959 | RFC5, HIST1H2BM, NEIL3, PCNA, HIST1H2AK, POLE2, HIST1H2BB |
| R-HSA-8852276 | The role of GTSE1 in G2/M progression after G2 checkpoint | 0.042552028 | 0.21276014 | CCNB2, CCNB1, RPN2, PLK1, CDK1, PSME2, GTSE1, TP53 |
| R-HSA-8852135 | Protein ubiquitination | 0.04505432 | 0.225271601 | HIST1H2BM, PCNA, UBE2S, UBE2T, HIST1H2BB, RAD18 |
| R-HSA-3301854 | Nuclear Pore Complex (NPC) Disassembly | 0.048117864 | 0.240589322 | NDC1, CCNB2, CCNB1, NUP107, CDK1 |
| R-HSA-211000 | Gene Silencing by RNA | 0.049801899 | 0.249009495 | NDC1, HIST1H2BM, ADSL, NUP107, HIST1H2AK, AGO1, HIST1H2BB |
