# Supplementary material for: Gene Expression Signature of BRAF Inhibitor Resistant Melanoma Spheroids
Source: Pathol Oncol Res. 2020 Jul 1;26(4):2557–66. doi: 10.1007/s12253-020-00837-9 (PMC7471197; doi:10.1007/s12253-020-00837-9)
Supplement: Supplementary file 4 — (DOCX 14 kb) [file 12253_2020_837_MOESM4_ESM.docx]

**Supplementary Table 4**

Significantly Downregulated genes in sensitive melanoma spheroid compare to sensitive monolayer grouped by molecular pathways

| **Pathway identifier** | **Pathway name** | **P-Value** | **FDR Value** | **Genes included (at least 5)** |
| --- | --- | --- | --- | --- |
| R-HSA-3371453 | Regulation of HSF1-mediated heat shock response | 0.006094953 | 0.617858 | HSPH1, RPS19BP1, HSPA1L, HSBP1, MRPL18, ATM |
| R-HSA-9024446 | NR1H2 and NR1H3-mediated signaling | 0.007506727 | 0.617858 | RXRB, SCD, APOC1, APOD, APOE |
| R-HSA-3371556 | Cellular response to heat stress | 0.008494393 | 0.617858 | HSPH1, RPS19BP1, HSPA1L, HSBP1, MRPL18, ATM |
| R-HSA-1989781 | PPARA activates gene expression | 0.023110549 | 0.617858 | RXRB, HMGCS1, ACOX1, ACSL1, HMGCR, NR1D1, MED7, MED13L |
| R-HSA-400206 | Regulation of lipid metabolism by Peroxisome proliferator-activated receptor alpha (PPARalpha) | 0.025026582 | 0.617858 | RXRB, HMGCS1, ACOX1, ACSL1, HMGCR, NR1D1, MED7, MED13L |
| R-HSA-191273 | Cholesterol biosynthesis | 0.025949409 | 0.617858 | IDI1, HMGCS1, DHCR24, MSMO1, HMGCR, ACAT2 |
| R-HSA-2262752 | Cellular responses to stress | 0.030486666 | 0.617858 | DCTN6, DYNC1H1, CEBPB, HSPA1L, RING1, RPS19BP1, ANAPC16, HSBP1, EPAS1, MRPL18, UBE2D3, FOS, PHC3, HSPH1, RHEB, RPS6KA2, ATP6V1B2, ATM, TPP1, PSMF1, RPS27A, ATP6V0D1, ATP6V1D |
| R-HSA-9006936 | Signaling by TGF-beta family members | 0.039820988 | 0.617858 | PPP1R15A, SMAD2, BMPR2, UBE2D3, RPS27A, BMPR1B, JUNB, BMPR1A |
| R-HSA-8953897 | Cellular responses to external stimuli | 0.043376647 | 0.617858 | DCTN6, DYNC1H1, CEBPB, HSPA1L, RING1, RPS19BP1, ANAPC16, HSBP1, EPAS1, MRPL18, UBE2D3, FOS, PHC3, HSPH1, RHEB, RPS6KA2, ATP6V1B2, ATM, TPP1, PSMF1, RPS27A, ATP6V0D1, ATP6V1D |
| R-HSA-6785807 | Interleukin-4 and Interleukin-13 signaling | 0.044013241 | 0.617858 | IRF4, PIM1, BCL2, HMOX1, FOS, PTGS2, JUNB |
| R-HSA-5633008 | TP53 Regulates Transcription of Cell Death Genes | 0.049116436 | 0.617858 | PPP1R13B, TP53INP1, ATM, NDRG1, ZNF420 |
