# Supplementary material for: Gene Expression Signature of BRAF Inhibitor Resistant Melanoma Spheroids
Source: Pathol Oncol Res. 2020 Jul 1;26(4):2557–66. doi: 10.1007/s12253-020-00837-9 (PMC7471197; doi:10.1007/s12253-020-00837-9)
Supplement: Supplementary file 5 — (DOCX 42 kb) [file 12253_2020_837_MOESM5_ESM.docx]

**Supplementary Table 5**

List of genes which are differentially expressed in cell lines derived from resistant melanoma spheroid comparing to resistant monolayer cultures (N=297). Genes are sorted by fold change (upregulated if fold change >1; downregulated if fold change < 1)

| Sl.  No. | Gene  symbol | Description | Fold  change | Alteration |
| --- | --- | --- | --- | --- |
| 1 | ACTG2 | actin gamma 2, smooth muscle | 8.799 | Upregulated |
| 2 | PKP2 | plakophilin 2 | 3.961 | Upregulated |
| 3 | RPS9 | ribosomal protein S9 | 3.367 | Upregulated |
| 4 | LCP1 | lymphocyte cytosolic protein 1 | 3.041 | Upregulated |
| 5 | REXO1L1P | REXO1 like 1, pseudogene | 2.798 | Upregulated |
| 6 | NLGN4Y | neuroligin 4 Y-linked | 2.794 | Upregulated |
| 7 | RASGRP3 | RAS guanyl releasing protein 3 | 2.764 | Upregulated |
| 8 | TSHZ2 | teashirt zinc finger homeobox 2 | 2.753 | Upregulated |
| 9 | NAP1L3 | nucleosome assembly protein 1 like 3 | 2.741 | Upregulated |
| 10 | APBB3 | amyloid beta precursor protein binding family B member 3 | 2.666 | Upregulated |
| 11 | SCN8A | sodium voltage-gated channel alpha subunit 8 | 2.664 | Upregulated |
| 12 | DEFB124 | defensin beta 124 | 2.625 | Upregulated |
| 13 | AGAP9 | ArfGAP with GTPase domain, ankyrin repeat and PH domain 9 | 2.574 | Upregulated |
| 14 | TGIF1 | TGFB induced factor homeobox 1 | 2.499 | Upregulated |
| 15 | HLA-F | major histocompatibility complex, class I, F | 2.469 | Upregulated |
| 16 | LENG8 | leukocyte receptor cluster member 8 | 2.459 | Upregulated |
| 17 | LOC388022 |  | 2.448 | Upregulated |
| 18 | ZNF257 | zinc finger protein 257 | 2.442 | Upregulated |
| 19 | MOG | myelin oligodendrocyte glycoprotein | 2.428 | Upregulated |
| 20 | PIGF | phosphatidylinositol glycan anchor biosynthesis class F | 2.423 | Upregulated |
| 21 | ELP5 | elongator acetyltransferase complex subunit 5 | 2.302 | Upregulated |
| 22 | ADHFE1 | alcohol dehydrogenase iron containing 1 | 2.276 | Upregulated |
| 23 | SLAMF8 | SLAM family member 8 | 2.254 | Upregulated |
| 24 | PCDHB2 | protocadherin beta 2 | 2.250 | Upregulated |
| 25 | TBX18 | T-box transcription factor 18 | 2.219 | Upregulated |
| 26 | PSMB6 | proteasome 20S subunit beta 6 | 2.191 | Upregulated |
| 27 | SRGN | serglycin | 2.190 | Upregulated |
| 28 | RING1 | ring finger protein 1 | 2.170 | Upregulated |
| 29 | CCNL2 |  | 2.121 | Upregulated |
| 30 | SLCO2B1 | solute carrier organic anion transporter family member 2B1 | 2.120 | Upregulated |
| 31 | GSTM4 | glutathione S-transferase mu 4 | 2.101 | Upregulated |
| 32 | CCNL2 | cyclin L2 | 2.090 | Upregulated |
| 33 | THY1 | Thy-1 cell surface antigen | 2.084 | Upregulated |
| 34 | DCUN1D3 | defective in cullin neddylation 1 domain containing 3 | 2.084 | Upregulated |
| 35 | PLCB4 | phospholipase C beta 4 | 2.068 | Upregulated |
| 36 | MBD3L5 | methyl-CpG binding domain protein 3 like 5 | 2.067 | Upregulated |
| 37 | TMEM47 | transmembrane protein 47 | 2.048 | Upregulated |
| 38 | PDCD1LG2 | programmed cell death 1 ligand 2 | 2.041 | Upregulated |
| 39 | DDAH1 | dimethylarginine dimethylaminohydrolase 1 | 2.040 | Upregulated |
| 40 | UCP2 | uncoupling protein 2 | 2.036 | Upregulated |
| 41 | LINC00173 | long intergenic non-protein coding RNA 173 | 2.020 | Upregulated |
| 42 | NOX4 | NADPH oxidase 4 | 2.001 | Upregulated |
| 43 | TMEM255B | transmembrane protein 255B | 1.999 | Upregulated |
| 44 | SLC25A27 | solute carrier family 25 member 27 | 1.993 | Upregulated |
| 45 | LHFP |  | 1.974 | Upregulated |
| 46 | MLF2 | myeloid leukemia factor 2 | 1.968 | Upregulated |
| 47 | MANBAL | mannosidase beta like | 1.948 | Upregulated |
| 48 | HIST1H2BM |  | 1.942 | Upregulated |
| 49 | ABHD4 | abhydrolase domain containing 4 | 1.899 | Upregulated |
| 50 | STRADB | STE20 related adaptor beta | 1.892 | Upregulated |
| 51 | ARRDC1-AS1 | ARRDC1 antisense RNA 1 | 1.888 | Upregulated |
| 52 | LCE2C | late cornified envelope 2C | 1.882 | Upregulated |
| 53 | GINS1 | GINS complex subunit 1 | 1.870 | Upregulated |
| 54 | SPIN4 | spindlin family member 4 | 1.847 | Upregulated |
| 55 | LRRFIP1 | LRR binding FLII interacting protein 1 | 1.846 | Upregulated |
| 56 | NDUFS3 | NADH:ubiquinone oxidoreductase core subunit S3 | 1.845 | Upregulated |
| 57 | SPIN1 | spindlin 1 | 1.843 | Upregulated |
| 58 | VAMP7 | vesicle associated membrane protein 7 | 1.821 | Upregulated |
| 59 | DPY19L2P1 | DPY19L2 pseudogene 1 | 1.814 | Upregulated |
| 60 | ATP6V0A1 | ATPase H+ transporting V0 subunit a1 | 1.801 | Upregulated |
| 61 | WSB1 | WD repeat and SOCS box containing 1 | 1.768 | Upregulated |
| 62 | AHSA2 |  | 1.745 | Upregulated |
| 63 | XCL1 | X-C motif chemokine ligand 1 | 1.727 | Upregulated |
| 64 | PRKAB2 | protein kinase AMP-activated non-catalytic subunit beta 2 | 1.724 | Upregulated |
| 65 | LOC642533 |  | 1.697 | Upregulated |
| 66 | CD22 | CD22 molecule | 1.684 | Upregulated |
| 67 | CYB5R2 | cytochrome b5 reductase 2 | 1.656 | Upregulated |
| 68 | MRAP2 | melanocortin 2 receptor accessory protein 2 | 1.654 | Upregulated |
| 69 | CALCOCO1 | calcium binding and coiled-coil domain 1 | 1.653 | Upregulated |
| 70 | POTEF | POTE ankyrin domain family member F | 1.649 | Upregulated |
| 71 | ELOVL1 | ELOVL fatty acid elongase 1 | 1.582 | Upregulated |
| 72 | TXLNGY | taxilin gamma pseudogene, Y-linked | 1.580 | Upregulated |

| 1 | VTRNA1-1 | vault RNA 1-1 | 0.059 | Downregulated |
| --- | --- | --- | --- | --- |
| 2 | RNU4-1 | RNA, U4 small nuclear 1 | 0.068 | Downregulated |
| 3 | RNU4-2 | RNA, U4 small nuclear 2 | 0.080 | Downregulated |
| 4 | SNORA38B | small nucleolar RNA, H/ACA box 38B | 0.146 | Downregulated |
| 5 | SCARNA6 | small Cajal body-specific RNA 6 | 0.147 | Downregulated |
| 6 | RAC1 | Rac family small GTPase 1 | 0.150 | Downregulated |
| 7 | SNORA20 | small nucleolar RNA, H/ACA box 20 | 0.151 | Downregulated |
| 8 | GLUD1 | glutamate dehydrogenase 1 | 0.189 | Downregulated |
| 9 | BGN | biglycan | 0.202 | Downregulated |
| 10 | BCHE | butyrylcholinesterase | 0.204 | Downregulated |
| 11 | SNORA7B | small nucleolar RNA, H/ACA box 7B | 0.206 | Downregulated |
| 12 | C7orf69 | chromosome 7 open reading frame 69 | 0.229 | Downregulated |
| 13 | FAM21C |  | 0.245 | Downregulated |
| 14 | RPPH1 | ribonuclease P RNA component H1 | 0.252 | Downregulated |
| 15 | SCARNA5 | small Cajal body-specific RNA 5 | 0.256 | Downregulated |
| 16 | RNU5B-1 | RNA, U5B small nuclear 1 | 0.259 | Downregulated |
| 17 | SNORD15B | small nucleolar RNA, C/D box 15B | 0.273 | Downregulated |
| 18 | RNU5E-1 | RNA, U5E small nuclear 1 | 0.299 | Downregulated |
| 19 | USMG5 |  | 0.300 | Downregulated |
| 20 | RPL13 | ribosomal protein L13 | 0.300 | Downregulated |
| 21 | SNORA49 | small nucleolar RNA, H/ACA box 49 | 0.305 | Downregulated |
| 22 | RNU4ATAC | RNA, U4atac small nuclear (U12-dependent splicing) | 0.306 | Downregulated |
| 23 | SNORA23 | small nucleolar RNA, H/ACA box 23 | 0.308 | Downregulated |
| 24 | RPS12 | ribosomal protein S12 | 0.308 | Downregulated |
| 25 | USMG5 |  | 0.309 | Downregulated |
| 26 | TMEM203 | transmembrane protein 203 | 0.309 | Downregulated |
| 27 | SCARNA10 | small Cajal body-specific RNA 10 | 0.318 | Downregulated |
| 28 | BNIP3 | BCL2 interacting protein 3 | 0.322 | Downregulated |
| 29 | PTGS2 | prostaglandin-endoperoxide synthase 2 | 0.326 | Downregulated |
| 30 | RPL24 | ribosomal protein L24 | 0.331 | Downregulated |
| 31 | SNORA80E | small nucleolar RNA, H/ACA box 80E | 0.331 | Downregulated |
| 32 | RAP1B | RAP1B, member of RAS oncogene family | 0.333 | Downregulated |
| 33 | SNORA60 | small nucleolar RNA, H/ACA box 60 | 0.337 | Downregulated |
| 34 | KLHDC10 | kelch domain containing 10 | 0.345 | Downregulated |
| 35 | ATL3 | atlastin GTPase 3 | 0.347 | Downregulated |
| 36 | RPL23P8 | ribosomal protein L23 pseudogene 8 | 0.347 | Downregulated |
| 37 | SFTA2 | surfactant associated 2 | 0.349 | Downregulated |
| 38 | SCG2 | secretogranin II | 0.349 | Downregulated |
| 39 | RPS27 | ribosomal protein S27 | 0.357 | Downregulated |
| 40 | AEBP1 | AE binding protein 1 | 0.357 | Downregulated |
| 41 | SNORA22 | small nucleolar RNA, H/ACA box 22 | 0.366 | Downregulated |
| 42 | RPL39L | ribosomal protein L39 like | 0.366 | Downregulated |
| 43 | RNU11 | RNA, U11 small nuclear | 0.368 | Downregulated |
| 44 | ACAA1 | acetyl-CoA acyltransferase 1 | 0.369 | Downregulated |
| 45 | SNORA2A | small nucleolar RNA, H/ACA box 2A | 0.370 | Downregulated |
| 46 | SNORD105 | small nucleolar RNA, C/D box 105 | 0.375 | Downregulated |
| 47 | RPS7 | ribosomal protein S7 | 0.382 | Downregulated |
| 48 | EVI2A | ecotropic viral integration site 2A | 0.387 | Downregulated |
| 49 | TBCA | tubulin folding cofactor A | 0.387 | Downregulated |
| 50 | SNORD46 | small nucleolar RNA, C/D box 46 | 0.389 | Downregulated |
| 51 | RAB23 | RAB23, member RAS oncogene family | 0.390 | Downregulated |
| 52 | ZBTB38 | zinc finger and BTB domain containing 38 | 0.395 | Downregulated |
| 53 | FAM175A |  | 0.396 | Downregulated |
| 54 | COX7B | cytochrome c oxidase subunit 7B | 0.400 | Downregulated |
| 55 | ARHGAP24 | Rho GTPase activating protein 24 | 0.403 | Downregulated |
| 56 | TXN | thioredoxin | 0.406 | Downregulated |
| 57 | KRCC1 | lysine rich coiled-coil 1 | 0.406 | Downregulated |
| 58 | HERC4 | HECT and RLD domain containing E3 ubiquitin protein ligase 4 | 0.410 | Downregulated |
| 59 | ZNF518A | zinc finger protein 518A | 0.412 | Downregulated |
| 60 | IGF1R | insulin like growth factor 1 receptor | 0.416 | Downregulated |
| 61 | SNORD94 | small nucleolar RNA, C/D box 94 | 0.417 | Downregulated |
| 62 | BBS12 | Bardet-Biedl syndrome 12 | 0.419 | Downregulated |
| 63 | STAG1 | stromal antigen 1 | 0.424 | Downregulated |
| 64 | FANCF | FA complementation group F | 0.429 | Downregulated |
| 65 | RPS27A | ribosomal protein S27a | 0.430 | Downregulated |
| 66 | RPS24 | ribosomal protein S24 | 0.430 | Downregulated |
| 67 | SNRPE | small nuclear ribonucleoprotein polypeptide E | 0.430 | Downregulated |
| 68 | SKIL | SKI like proto-oncogene | 0.432 | Downregulated |
| 69 | DNAH14 | dynein axonemal heavy chain 14 | 0.436 | Downregulated |
| 70 | DDIT4 | DNA damage inducible transcript 4 | 0.439 | Downregulated |
| 71 | LSM14B | LSM family member 14B | 0.445 | Downregulated |
| 72 | RPL10A | ribosomal protein L10a | 0.445 | Downregulated |
| 73 | TNS3 | tensin 3 | 0.445 | Downregulated |
| 74 | RPS6 | ribosomal protein S6 | 0.447 | Downregulated |
| 75 | ASCC1 | activating signal cointegrator 1 complex subunit 1 | 0.447 | Downregulated |
| 76 | SCARNA7 | small Cajal body-specific RNA 7 | 0.448 | Downregulated |
| 77 | MMP16 | matrix metallopeptidase 16 | 0.448 | Downregulated |
| 78 | NME7 | NME/NM23 family member 7 | 0.449 | Downregulated |
| 79 | PUS7 | pseudouridine synthase 7 | 0.450 | Downregulated |
| 80 | CHCHD1 | coiled-coil-helix-coiled-coil-helix domain containing 1 | 0.450 | Downregulated |
| 81 | POP5 | POP5 homolog, ribonuclease P/MRP subunit | 0.451 | Downregulated |
| 82 | IFIT2 | interferon induced protein with tetratricopeptide repeats 2 | 0.451 | Downregulated |
| 83 | NPM1 | nucleophosmin 1 | 0.451 | Downregulated |
| 84 | SLC29A4 | solute carrier family 29 member 4 | 0.453 | Downregulated |
| 85 | RPL23AP32 | ribosomal protein L23a pseudogene 32 | 0.456 | Downregulated |
| 86 | ADSS |  | 0.457 | Downregulated |
| 87 | C5 | complement C5 | 0.457 | Downregulated |
| 88 | MRTO4 | MRT4 homolog, ribosome maturation factor | 0.458 | Downregulated |
| 89 | LBH | LBH regulator of WNT signaling pathway | 0.469 | Downregulated |
| 90 | SLIT3 | slit guidance ligand 3 | 0.469 | Downregulated |
| 91 | THAP2 | THAP domain containing 2 | 0.471 | Downregulated |
| 92 | STK19 | serine/threonine kinase 19 | 0.473 | Downregulated |
| 93 | OCR1 |  | 0.474 | Downregulated |
| 94 | DCUN1D1 | defective in cullin neddylation 1 domain containing 1 | 0.475 | Downregulated |
| 95 | GIMAP2 | GTPase, IMAP family member 2 | 0.475 | Downregulated |
| 96 | DHRS7 | dehydrogenase/reductase 7 | 0.476 | Downregulated |
| 97 | WDR78 | WD repeat domain 78 | 0.477 | Downregulated |
| 98 | PPP3CB | protein phosphatase 3 catalytic subunit beta | 0.480 | Downregulated |
| 99 | BLVRA | biliverdin reductase A | 0.482 | Downregulated |
| 100 | ADCK2 | aarF domain containing kinase 2 | 0.482 | Downregulated |
| 101 | RRP15 | ribosomal RNA processing 15 homolog | 0.484 | Downregulated |
| 102 | RPS4Y1 | ribosomal protein S4 Y-linked 1 | 0.485 | Downregulated |
| 103 | EHHADH | enoyl-CoA hydratase and 3-hydroxyacyl CoA dehydrogenase | 0.485 | Downregulated |
| 104 | ZNF770 | zinc finger protein 770 | 0.488 | Downregulated |
| 105 | MRPS33 | mitochondrial ribosomal protein S33 | 0.488 | Downregulated |
| 106 | MRPL36 | mitochondrial ribosomal protein L36 | 0.489 | Downregulated |
| 107 | THNSL1 | threonine synthase like 1 | 0.490 | Downregulated |
| 108 | GDF15 | growth differentiation factor 15 | 0.490 | Downregulated |
| 109 | PFDN6 | prefoldin subunit 6 | 0.491 | Downregulated |
| 110 | CEP19 | centrosomal protein 19 | 0.492 | Downregulated |
| 111 | IARS2 | isoleucyl-tRNA synthetase 2, mitochondrial | 0.493 | Downregulated |
| 112 | MERTK | MER proto-oncogene, tyrosine kinase | 0.494 | Downregulated |
| 113 | SLMO2 |  | 0.495 | Downregulated |
| 114 | CENPBD1P1 | CENPB DNA-binding domains containing 1 pseudogene 1 | 0.496 | Downregulated |
| 115 | OARD1 | O-acyl-ADP-ribose deacylase 1 | 0.496 | Downregulated |
| 116 | PINK1-AS | PINK1 antisense RNA | 0.496 | Downregulated |
| 117 | ZNF271 |  | 0.497 | Downregulated |
| 118 | PSMB7 | proteasome 20S subunit beta 7 | 0.497 | Downregulated |
| 119 | FAM35A |  | 0.499 | Downregulated |
| 120 | IFT81 | intraflagellar transport 81 | 0.505 | Downregulated |
| 121 | JPX | JPX transcript, XIST activator | 0.505 | Downregulated |
| 122 | VTRNA1-3 | vault RNA 1-3 | 0.507 | Downregulated |
| 123 | LOC100132167 |  | 0.509 | Downregulated |
| 124 | MRPL24 | mitochondrial ribosomal protein L24 | 0.509 | Downregulated |
| 125 | MRPL1 | mitochondrial ribosomal protein L1 | 0.511 | Downregulated |
| 126 | HIST1H2BB |  | 0.516 | Downregulated |
| 127 | IKBIP | IKBKB interacting protein | 0.521 | Downregulated |
| 128 | ZNF654 | zinc finger protein 654 | 0.524 | Downregulated |
| 129 | CCDC58 | coiled-coil domain containing 58 | 0.525 | Downregulated |
| 130 | MRPS23 | mitochondrial ribosomal protein S23 | 0.525 | Downregulated |
| 131 | P4HA1 | prolyl 4-hydroxylase subunit alpha 1 | 0.526 | Downregulated |
| 132 | CSPG4 | chondroitin sulfate proteoglycan 4 | 0.526 | Downregulated |
| 133 | TOR1AIP2 | torsin 1A interacting protein 2 | 0.526 | Downregulated |
| 134 | BEND6 | BEN domain containing 6 | 0.530 | Downregulated |
| 135 | MRPS36 | mitochondrial ribosomal protein S36 | 0.534 | Downregulated |
| 136 | MOCOS | molybdenum cofactor sulfurase | 0.535 | Downregulated |
| 137 | LTBP1 | latent transforming growth factor beta binding protein 1 | 0.535 | Downregulated |
| 138 | CD82 | CD82 molecule | 0.536 | Downregulated |
| 139 | KDM4C | lysine demethylase 4C | 0.538 | Downregulated |
| 140 | THAP5 | THAP domain containing 5 | 0.538 | Downregulated |
| 141 | MRPS22 | mitochondrial ribosomal protein S22 | 0.539 | Downregulated |
| 142 | VEGFA | vascular endothelial growth factor A | 0.539 | Downregulated |
| 143 | IL1RAP | interleukin 1 receptor accessory protein | 0.542 | Downregulated |
| 144 | HOXB7 | homeobox B7 | 0.542 | Downregulated |
| 145 | ST6GALNAC3 | ST6 N-acetylgalactosaminide alpha-2,6-sialyltransferase 3 | 0.543 | Downregulated |
| 146 | GRB10 | growth factor receptor bound protein 10 | 0.543 | Downregulated |
| 147 | EIF3E | eukaryotic translation initiation factor 3 subunit E | 0.546 | Downregulated |
| 148 | CLSTN1 | calsyntenin 1 | 0.546 | Downregulated |
| 149 | HRSP12 |  | 0.548 | Downregulated |
| 150 | EVI5 | ecotropic viral integration site 5 | 0.549 | Downregulated |
| 151 | EVI2B | ecotropic viral integration site 2B | 0.549 | Downregulated |
| 152 | CENPF | centromere protein F | 0.549 | Downregulated |
| 153 | YEATS2 | YEATS domain containing 2 | 0.549 | Downregulated |
| 154 | PFDN5 | prefoldin subunit 5 | 0.551 | Downregulated |
| 155 | S100A6 | S100 calcium binding protein A6 | 0.551 | Downregulated |
| 156 | NHP2 | NHP2 ribonucleoprotein | 0.551 | Downregulated |
| 157 | CHMP1B | charged multivesicular body protein 1B | 0.553 | Downregulated |
| 158 | RNF130 | ring finger protein 130 | 0.555 | Downregulated |
| 159 | HLTF | helicase like transcription factor | 0.555 | Downregulated |
| 160 | PPAPDC2 |  | 0.556 | Downregulated |
| 161 | PREX1 | phosphatidylinositol-3,4,5-trisphosphate dependent Rac exchange factor 1 | 0.556 | Downregulated |
| 162 | SERTAD4 | SERTA domain containing 4 | 0.557 | Downregulated |
| 163 | GCLM | glutamate-cysteine ligase modifier subunit | 0.557 | Downregulated |
| 164 | HIST1H1C |  | 0.559 | Downregulated |
| 165 | TMED9 | transmembrane p24 trafficking protein 9 | 0.561 | Downregulated |
| 166 | FLOT1 | flotillin 1 | 0.562 | Downregulated |
| 167 | FAM129A |  | 0.564 | Downregulated |
| 168 | LPCAT1 | lysophosphatidylcholine acyltransferase 1 | 0.566 | Downregulated |
| 169 | ZDHHC21 | zinc finger DHHC-type palmitoyltransferase 21 | 0.566 | Downregulated |
| 170 | PRSS23 | serine protease 23 | 0.567 | Downregulated |
| 171 | KIAA1586 | KIAA1586 | 0.569 | Downregulated |
| 172 | EDF1 | endothelial differentiation related factor 1 | 0.569 | Downregulated |
| 173 | IGSF3 | immunoglobulin superfamily member 3 | 0.569 | Downregulated |
| 174 | GPD2 | glycerol-3-phosphate dehydrogenase 2 | 0.569 | Downregulated |
| 175 | YAE1D1 |  | 0.571 | Downregulated |
| 176 | PIGN | phosphatidylinositol glycan anchor biosynthesis class N | 0.571 | Downregulated |
| 177 | REEP3 | receptor accessory protein 3 | 0.572 | Downregulated |
| 178 | MIPEP | mitochondrial intermediate peptidase | 0.574 | Downregulated |
| 179 | DNAJC11 | DnaJ heat shock protein family (Hsp40) member C11 | 0.574 | Downregulated |
| 180 | RRP36 | ribosomal RNA processing 36 | 0.574 | Downregulated |
| 181 | ERMP1 | endoplasmic reticulum metallopeptidase 1 | 0.574 | Downregulated |
| 182 | GFM1 | G elongation factor mitochondrial 1 | 0.575 | Downregulated |
| 183 | C8orf48 | chromosome 8 open reading frame 48 | 0.576 | Downregulated |
| 184 | DPH5 | diphthamide biosynthesis 5 | 0.577 | Downregulated |
| 185 | NIP7 | nucleolar pre-rRNA processing protein NIP7 | 0.580 | Downregulated |
| 186 | SNORD104 | small nucleolar RNA, C/D box 104 | 0.581 | Downregulated |
| 187 | RPL39 | ribosomal protein L39 | 0.587 | Downregulated |
| 188 | MRPL15 | mitochondrial ribosomal protein L15 | 0.588 | Downregulated |
| 189 | MRPS18A | mitochondrial ribosomal protein S18A | 0.588 | Downregulated |
| 190 | ZNF639 | zinc finger protein 639 | 0.589 | Downregulated |
| 191 | ROR1 | receptor tyrosine kinase like orphan receptor 1 | 0.589 | Downregulated |
| 192 | HNRNPF | heterogeneous nuclear ribonucleoprotein F | 0.592 | Downregulated |
| 193 | DMXL1 | Dmx like 1 | 0.592 | Downregulated |
| 194 | TMEM67 | transmembrane protein 67 | 0.594 | Downregulated |
| 195 | C18orf32 | chromosome 18 open reading frame 32 | 0.596 | Downregulated |
| 196 | TUBD1 | tubulin delta 1 | 0.596 | Downregulated |
| 197 | NDUFS4 | NADH:ubiquinone oxidoreductase subunit S4 | 0.596 | Downregulated |
| 198 | GPAM | glycerol-3-phosphate acyltransferase, mitochondrial | 0.596 | Downregulated |
| 199 | ATP5A1 |  | 0.597 | Downregulated |
| 200 | CMSS1 | cms1 ribosomal small subunit homolog | 0.597 | Downregulated |
| 201 | NSUN3 | NOP2/Sun RNA methyltransferase 3 | 0.599 | Downregulated |
| 202 | MRPS10 | mitochondrial ribosomal protein S10 | 0.600 | Downregulated |
| 203 | MDC1 | mediator of DNA damage checkpoint 1 | 0.600 | Downregulated |
| 204 | PCDHA5 | protocadherin alpha 5 | 0.601 | Downregulated |
| 205 | EIF2A | eukaryotic translation initiation factor 2A | 0.601 | Downregulated |
| 206 | PRKXP1 | PRKX pseudogene 1 | 0.601 | Downregulated |
| 207 | ARPC1A | actin related protein 2/3 complex subunit 1A | 0.602 | Downregulated |
| 208 | ZNF322 | zinc finger protein 322 | 0.607 | Downregulated |
| 209 | IGFBP3 | insulin like growth factor binding protein 3 | 0.609 | Downregulated |
| 210 | POLR1E | RNA polymerase I subunit E | 0.611 | Downregulated |
| 211 | ALCAM | activated leukocyte cell adhesion molecule | 0.611 | Downregulated |
| 212 | CARF | calcium responsive transcription factor | 0.612 | Downregulated |
| 213 | PPA1 | inorganic pyrophosphatase 1 | 0.613 | Downregulated |
| 214 | HEATR1 | HEAT repeat containing 1 | 0.614 | Downregulated |
| 215 | IFT57 | intraflagellar transport 57 | 0.615 | Downregulated |
| 216 | CTSL | cathepsin L | 0.617 | Downregulated |
| 217 | AHNAK | AHNAK nucleoprotein | 0.618 | Downregulated |
| 218 | LOXL2 | lysyl oxidase like 2 | 0.620 | Downregulated |
| 219 | SMC3 | structural maintenance of chromosomes 3 | 0.621 | Downregulated |
| 220 | FAM208B |  | 0.622 | Downregulated |
| 221 | ZNF605 | zinc finger protein 605 | 0.623 | Downregulated |
| 222 | ATR | ATR serine/threonine kinase | 0.624 | Downregulated |
| 223 | TRMT10C | tRNA methyltransferase 10C, mitochondrial RNase P subunit | 0.629 | Downregulated |
| 224 | DNAAF2 | dynein axonemal assembly factor 2 | 0.629 | Downregulated |
| 225 | UTP14A | UTP14A small subunit processome component | 0.630 | Downregulated |
