# Supplementary material for: Gene Expression Signature of BRAF Inhibitor Resistant Melanoma Spheroids
Source: Pathol Oncol Res. 2020 Jul 1;26(4):2557–66. doi: 10.1007/s12253-020-00837-9 (PMC7471197; doi:10.1007/s12253-020-00837-9)
Supplement: Supplementary file 6 — (DOCX 17 kb) [file 12253_2020_837_MOESM6_ESM.docx]

**Supplementary Table 6**

Significantly downregulated genes in resistant melanoma spheroid compare to resistant monolayer grouped by molecular pathways

| **Pathway identifier** | **Pathway name** | **P-Value** | **FDR Value** | **Genes included (at least 5)** |
| --- | --- | --- | --- | --- |
| R-HSA-72766 | Translation | 2.30E-13 | 2.00E-10 | GFM1, MRPS36, MRPS33, MRPS10, MRPL36, MRPL15, RPS4Y1, RPL10A, MRPL1, RPL13, IARS2, RPS27A, CHCHD1, RPL39, RPS12, EIF2A, RPL39L, RPS7, MRPS22, MRPS23, RPS6, MRPS18A, MRPL24, RPS27, PPA1, RPL24, EIF3E, RPS24 |
| R-HSA-72706 | GTP hydrolysis and joining of the 60S ribosomal subunit | 1.02E-09 | 2.96E-07 | RPL39L, RPS7, RPS6, RPS4Y1, RPL10A, RPS27, RPL24, RPL13, EIF3E, RPS27A, RPL39, RPS24, EIF2A, RPS12 |
| R-HSA-156827 | L13a-mediated translational silencing of Ceruloplasmin expression | 1.02E-09 | 2.96E-07 | RPL39L, RPS7, RPS6, RPS4Y1, RPL10A, RPS27, RPL24, RPL13, EIF3E, RPS27A, RPL39, RPS24, EIF2A, RPS12 |
| R-HSA-72689 | Formation of a pool of free 40S subunits | 1.56E-09 | 3.38E-07 | RPL39L, RPS7, RPS6, RPS4Y1, RPL10A, RPS27, RPL24, RPL13, EIF3E, RPS27A, RPL39, RPS24, RPS12 |
| R-HSA-72613 | Eukaryotic Translation Initiation | 3.13E-09 | 4.53E-07 | RPL39L, RPS7, RPS6, RPS4Y1, RPL10A, RPS27, RPL24, RPL13, EIF3E, RPS27A, RPL39, RPS24, EIF2A, RPS12 |
| R-HSA-72737 | Cap-dependent Translation Initiation | 3.13E-09 | 4.53E-07 | RPL39L, RPS7, RPS6, RPS4Y1, RPL10A, RPS27, RPL24, RPL13, EIF3E, RPS27A, RPL39, RPS24, EIF2A, RPS12 |
| R-HSA-156902 | Peptide chain elongation | 4.31E-09 | 5.34E-07 | RPL39L, RPS27, RPS7, RPS6, RPL24, RPL13, RPS4Y1, RPL10A, RPS27A, RPL39, RPS24, RPS12 |
| R-HSA-975956 | Nonsense Mediated Decay (NMD) independent of the Exon Junction Complex (EJC) | 7.12E-09 | 7.68E-07 | RPL39L, RPS27, RPS7, RPS6, RPL24, RPL13, RPS4Y1, RPL10A, RPS27A, RPL39, RPS24, RPS12 |
| R-HSA-156842 | Eukaryotic Translation Elongation | 8.04E-09 | 7.72E-07 | RPL39L, RPS27, RPS7, RPS6, RPL24, RPL13, RPS4Y1, RPL10A, RPS27A, RPL39, RPS24, RPS12 |
| R-HSA-8868773 | rRNA processing in the nucleus and cytosol | 1.15E-08 | 9.98E-07 | RPL39L, RPS7, NIP7, RPS6, HEATR1, RPS4Y1, RPL10A, RPS27, NHP2, RRP36, RPL24, RPL13, RPS27A, UTP14A, RPL39, RPS24, RPS12 |
| R-HSA-72764 | Eukaryotic Translation Termination | 1.29E-08 | 1.02E-06 | RPL39L, RPS27, RPS7, RPS6, RPL24, RPL13, RPS4Y1, RPL10A, RPS27A, RPL39, RPS24, RPS12 |
| R-HSA-6791226 | Major pathway of rRNA processing in the nucleolus and cytosol | 1.62E-08 | 1.17E-06 | RPL39L, RPS7, NIP7, RPS6, HEATR1, RPS4Y1, RPL10A, RPS27, RRP36, RPL24, RPL13, RPS27A, UTP14A, RPL39, RPS24, RPS12 |
| R-HSA-5389840 | Mitochondrial translation elongation | 2.54E-08 | 1.67E-06 | GFM1, MRPL1, MRPS36, MRPS22, MRPS33, MRPS23, MRPS10, MRPS18A, MRPL36, MRPL15, CHCHD1, MRPL24 |
| R-HSA-2408557 | Selenocysteine synthesis | 2.83E-08 | 1.71E-06 | RPL39L, RPS27, RPS7, RPS6, RPL24, RPL13, RPS4Y1, RPL10A, RPS27A, RPL39, RPS24, RPS12 |
| R-HSA-192823 | Viral mRNA Translation | 3.15E-08 | 1.71E-06 | RPL39L, RPS27, RPS7, RPS6, RPL24, RPL13, RPS4Y1, RPL10A, RPS27A, RPL39, RPS24, RPS12 |
| R-HSA-72312 | rRNA processing | 3.16E-08 | 1.71E-06 | RPL39L, TRMT10C, RPS7, NIP7, RPS6, HEATR1, RPS4Y1, RPL10A, RPS27, NHP2, RRP36, RPL24, RPL13, RPS27A, UTP14A, RPL39, RPS24, RPS12 |
| R-HSA-1799339 | SRP-dependent cotranslational protein targeting to membrane | 5.31E-08 | 2.71E-06 | RPL39L, RPS27, RPS7, RPS6, RPL24, RPL13, RPS4Y1, RPL10A, RPS27A, RPL39, RPS24, RPS12 |
| R-HSA-5368287 | Mitochondrial translation | 6.46E-08 | 3.10E-06 | GFM1, MRPL1, MRPS36, MRPS22, MRPS33, MRPS23, MRPS10, MRPS18A, MRPL36, MRPL15, CHCHD1, MRPL24 |
| R-HSA-72695 | Formation of the ternary complex, and subsequently, the 43S complex | 7.59E-08 | 3.41E-06 | RPS27, RPS7, RPS6, EIF3E, RPS4Y1, RPS27A, RPS24, EIF2A, RPS12 |
| R-HSA-927802 | Nonsense-Mediated Decay (NMD) | 8.73E-08 | 3.58E-06 | RPL39L, RPS27, RPS7, RPS6, RPL24, RPL13, RPS4Y1, RPL10A, RPS27A, RPL39, RPS24, RPS12 |
| R-HSA-975957 | Nonsense Mediated Decay (NMD) enhanced by the Exon Junction Complex (EJC) | 8.73E-08 | 3.58E-06 | RPL39L, RPS27, RPS7, RPS6, RPL24, RPL13, RPS4Y1, RPL10A, RPS27A, RPL39, RPS24, RPS12 |
| R-HSA-5419276 | Mitochondrial translation termination | 2.05E-07 | 7.99E-06 | MRPL1, MRPS36, MRPS22, MRPS33, MRPS23, MRPS10, MRPS18A, MRPL36, MRPL15, CHCHD1, MRPL24 |
| R-HSA-5368286 | Mitochondrial translation initiation | 2.55E-07 | 9.45E-06 | MRPL1, MRPS36, MRPS22, MRPS33, MRPS23, MRPS10, MRPS18A, MRPL36, MRPL15, CHCHD1, MRPL24 |
| R-HSA-72649 | Translation initiation complex formation | 2.66E-07 | 9.59E-06 | RPS27, RPS7, RPS6, EIF3E, RPS4Y1, RPS27A, RPS24, EIF2A, RPS12 |
| R-HSA-72702 | Ribosomal scanning and start codon recognition | 3.55E-07 | 1.21E-05 | RPS27, RPS7, RPS6, EIF3E, RPS4Y1, RPS27A, RPS24, EIF2A, RPS12 |
| R-HSA-376176 | Signaling by ROBO receptors | 3.93E-07 | 1.30E-05 | RPL39L, RPS7, RPS6, RPS4Y1, RPL10A, PSMB7, PPP3CB, RPS27, RPL24, RPL13, SLIT3, RAC1, RPS27A, RPL39, RPS24, RPS12 |
| R-HSA-72662 | Activation of the mRNA upon binding of the cap-binding complex and eIFs, and subsequent binding to 43S | 4.68E-07 | 1.50E-05 | RPS27, RPS7, RPS6, EIF3E, RPS4Y1, RPS27A, RPS24, EIF2A, RPS12 |
| R-HSA-9010553 | Regulation of expression of SLITs and ROBOs | 1.65E-06 | 5.11E-05 | RPL39L, RPS7, RPS6, RPS4Y1, RPL10A, PSMB7, RPS27, RPL24, RPL13, RPS27A, RPL39, RPS24, RPS12 |
| R-HSA-168273 | Influenza Viral RNA Transcription and Replication | 4.83E-06 | 1.45E-04 | RPL39L, RPS27, RPS7, RPS6, RPL24, RPL13, RPS4Y1, RPL10A, RPS27A, RPL39, RPS24, RPS12 |
| R-HSA-2408522 | Selenoamino acid metabolism | 7.04E-06 | 2.04E-04 | RPL39L, RPS27, RPS7, RPS6, RPL24, RPL13, RPS4Y1, RPL10A, RPS27A, RPL39, RPS24, RPS12 |
| R-HSA-168255 | Influenza Life Cycle | 1.07E-05 | 3.00E-04 | RPL39L, RPS27, RPS7, RPS6, RPL24, RPL13, RPS4Y1, RPL10A, RPS27A, RPL39, RPS24, RPS12 |
| R-HSA-168254 | Influenza Infection | 2.00E-05 | 5.39E-04 | RPL39L, RPS27, RPS7, RPS6, RPL24, RPL13, RPS4Y1, RPL10A, RPS27A, RPL39, RPS24, RPS12 |
| R-HSA-8953854 | Metabolism of RNA | 4.77E-04 | 0.012393 | POP5, NIP7, HEATR1, RPS4Y1, RPL10A, RNU11, PSMB7, RPPH1, RRP36, RPL13, RPS27A, RPL39, RNU4ATAC, UTP14A, RPS12, RPL39L, TRMT10C, RPS7, RPS6, PUS7, RPS27, HNRNPF, NHP2, RPL24, SNRPE, RPS24 |
| R-HSA-6790901 | rRNA modification in the nucleus and cytosol | 0.001998693 | 0.047969 | RPS7, RPS6, HEATR1, NHP2, RRP36, UTP14A |
| R-HSA-422475 | Axon guidance | 0.002322366 | 0.055737 | RPL39L, RPS7, RPS6, ARPC1A, RPS4Y1, RPL10A, PSMB7, PPP3CB, RPS27, ALCAM, GRB10, RPL24, RPL13, SLIT3, RAC1, RPS27A, RPL39, RPS24, RPS12 |
| R-HSA-5663205 | Infectious disease | 0.004661982 | 0.107226 | RPL39L, NPM1, RPS7, RPS6, RPS4Y1, RPL10A, PSMB7, RPS27, RPL24, RPL13, RAC1, RPS27A, RPL39, RPS24, ATR, RPS12 |
| R-HSA-5693607 | Processing of DNA double-strand break ends | 0.006266444 | 0.131595 | MDC1, FAM175A, RPS27A, HIST1H2BB, ATR |
| R-HSA-392499 | Metabolism of proteins | 0.007341968 | 0.146839 | MDC1, GFM1, PIGN, MRPS10, MRPL36, RPL10A, SMC3, PRSS23, MRPL1, CHCHD1, RPL39, RPS12, EIF2A, TMED9, RPS7, MRPS22, IGFBP3, MRPS23, RPS6, MRPS18A, PPA1, RPL24, ST6GALNAC3, HIST1H2BB, SCG2, PFDN5, PFDN6, MRPS36, MRPS33, DCUN1D1, MRPL15, TXN, RPS4Y1, LTBP1, PSMB7, RAB23, HLTF, RPL13, DPH5, IARS2, RPS27A, RPL39L, BCHE, NPM1, TBCA, MRPL24, STAG1, RPS27, FAM175A, EIF3E, RPS24 |
| R-HSA-69481 | G2/M Checkpoints | 0.022827267 | 0.328794 | MDC1, PSMB7, FAM175A, RPS27A, HIST1H2BB, ATR |
| R-HSA-5693567 | HDR through Homologous Recombination (HRR) or Single Strand Annealing (SSA) | 0.026095622 | 0.34649 | MDC1, FAM175A, RPS27A, HIST1H2BB, ATR |
| R-HSA-5693538 | Homology Directed Repair | 0.031766266 | 0.381195 | MDC1, FAM175A, RPS27A, HIST1H2BB, ATR |
|  |  |  |  |  |
