# Supplementary material for: Gene Expression Signature of BRAF Inhibitor Resistant Melanoma Spheroids
Source: Pathol Oncol Res. 2020 Jul 1;26(4):2557–66. doi: 10.1007/s12253-020-00837-9 (PMC7471197; doi:10.1007/s12253-020-00837-9)
Supplement: Supplementary file 7 — (DOCX 17 kb) [file 12253_2020_837_MOESM7_ESM.docx]

**Supplementary Table 7**

**List of common altered gene (N­=46) between BRAFi sensitive spheroid and resistant spheroid.**

| Sl.  No. | Gene  symbol | Description | Fold-change  (in sensitive spheroids^1^) | Fold-change  (in resistant spheroid^2^) |
| --- | --- | --- | --- | --- |
| 1 | DEFB124 | defensin beta 124 | 1.45 | 2.63 |
| 2 | MBD3L5 | methyl-CpG binding domain protein 3 like 5 | 1.90 | 2.07 |
| 3 | DDAH1 | dimethylarginine dimethylaminohydrolase 1 | 3.82 | 2.04 |
| 4 | UCP2 | uncoupling protein 2 | 2.86 | 2.04 |
| 5 | MLF2 | myeloid leukemia factor 2 | 1.32 | 1.97 |
| 6 | HIST1H2BM |  | 12.91 | 1.94 |
| 8 | SNORA20 | small nucleolar RNA, H/ACA box 20 | 0.31 | 0.15 |
| 9 | FAM21C |  | 0.53 | 0.25 |
| 10 | RPPH1 | ribonuclease P RNA component H1 | 0.53 | 0.25 |
| 11 | SNORD15B | small nucleolar RNA, C/D box 15B | 0.33 | 0.27 |
| 12 | USMG5 |  | 0.39 | 0.30 |
| 13 | SNORA49 | small nucleolar RNA, H/ACA box 49 | 0.66 | 0.31 |
| 14 | SCARNA10 | small Cajal body-specific RNA 10 | 0.59 | 0.32 |
| 15 | PTGS2 | prostaglandin-endoperoxide synthase 2 | 0.22 | 0.33 |
| 16 | RPL24 | ribosomal protein L24 | 0.58 | 0.33 |
| 17 | KLHDC10 | kelch domain containing 10 | 0.39 | 0.35 |
| 18 | SNORD105 | small nucleolar RNA, C/D box 105 | 0.64 | 0.38 |
| 19 | EVI2A | ecotropic viral integration site 2A | 0.72 | 0.39 |
| 20 | SNORD46 | small nucleolar RNA, C/D box 46 | 0.33 | 0.39 |
| 21 | IGF1R | insulin like growth factor 1 receptor | 0.64 | 0.42 |
| 22 | BBS12 | Bardet-Biedl syndrome 12 | 0.66 | 0.42 |
| 23 | RPS27A | ribosomal protein S27a | 0.53 | 0.43 |
| 24 | LSM14B | LSM family member 14B | 0.35 | 0.45 |
| 25 | MMP16 | matrix metallopeptidase 16 | 0.79 | 0.45 |
| 26 | WDR78 | WD repeat domain 78 | 0.24 | 0.48 |
| 27 | PFDN6 | prefoldin subunit 6 | 0.51 | 0.49 |
| 28 | CENPBD1P1 | CENPB DNA-binding domains containing 1 pseudogene 1 | 0.31 | 0.50 |
| 29 | OARD1 | O-acyl-ADP-ribose deacylase 1 | 0.40 | 0.50 |
| 30 | FAM35A |  | 0.63 | 0.50 |
| 31 | P4HA1 | prolyl 4-hydroxylase subunit alpha 1 | 0.91 | 0.53 |
| 32 | CSPG4 | chondroitin sulfate proteoglycan 4 | 0.76 | 0.53 |
| 33 | MRPS22 | mitochondrial ribosomal protein S22 | 0.63 | 0.54 |
| 34 | ST6GALNAC3 | ST6 N-acetylgalactosaminide alpha-2,6-sialyltransferase 3 | 0.59 | 0.54 |
| 35 | PFDN5 | prefoldin subunit 5 | 0.49 | 0.55 |
| 36 | S100A6 | S100 calcium binding protein A6 | 0.81 | 0.55 |
| 37 | CHMP1B | charged multivesicular body protein 1B | 0.21 | 0.55 |
| 38 | FLOT1 | flotillin 1 | 0.53 | 0.56 |
| 39 | SNORD104 | small nucleolar RNA, C/D box 104 | 0.30 | 0.58 |
| 40 | DMXL1 | Dmx like 1 | 0.51 | 0.59 |
| 41 | MRPS10 | mitochondrial ribosomal protein S10 | 0.77 | 0.60 |
| 42 | PRKXP1 | PRKX pseudogene 1 | 0.42 | 0.60 |
| 43 | ARPC1A | actin related protein 2/3 complex subunit 1A | 0.83 | 0.60 |
| 44 | ZNF322 | zinc finger protein 322 | 0.50 | 0.61 |
| 45 | CARF | calcium responsive transcription factor | 0.56 | 0.61 |
| 46 | CEP19 | dynein axonemal assembly factor 2 | 0.73 | 0.63 |

^1^Comparison of gene expression between sensitive spheroids and sensitive monolayer cultures.

^2^Comparison of gene expression between resistant spheroids and resistant monolayer cultures.
