# Supplementary material for: Gene Expression Signature of BRAF Inhibitor Resistant Melanoma Spheroids
Source: Pathol Oncol Res. 2020 Jul 1;26(4):2557–66. doi: 10.1007/s12253-020-00837-9 (PMC7471197; doi:10.1007/s12253-020-00837-9)
Supplement: Supplementary file 8 — (DOCX 13 kb) [file 12253_2020_837_MOESM8_ESM.docx]

**Supplementary Table 8. Relative mRNA expression of BRAFi sensitive and resistant cell lines**

**cultured in 2D and 3D Method**

|  | WM983A | | WM983B | | WM983A | | WM983B | |
| --- | --- | --- | --- | --- | --- | --- | --- | --- |
|  | (sensitive) | | (sensitive) | | (resistant) | | (resistant) | |
| Genes | 2D | 3D | 2D | 3D | 2D | 3D | 2D | 3D |
| ABHD4 | 0.007 | 0.018 | 0.010 | 0.006 | 0.026 | 0.039 | 0.007 | 0.069 |
| HIST1H2BB | 0.196 | 0.167 | 0.184 | 0.211 | 0.112 | 0.013 | 0.038 | 0.025 |
| SCN8A | 0.000 | 0.000 | 0.000 | 0.006 | 0.009 | 0.012 | 0.004 | 0.011 |
| CMSS1 | 0.057 | 0.045 | 0.030 | 0.055 | 0.027 | 0.010 | 0.009 | 0.021 |
| DCUN1D1 | 0.062 | 0.068 | 0.105 | 0.112 | 0.033 | 0.019 | 0.020 | 0.032 |
| IKBIP | 0.015 | 0.033 | 0.038 | 0.011 | 0.015 | 0.012 | 0.012 | 0.023 |
| SMC3 | 0.053 | 0.045 | 0.023 | 0.089 | 0.011 | 0.011 | 0.005 | 0.018 |
| ZNF639 | 0.031 | 0.037 | 0.027 | 0.085 | 0.021 | 0.019 | 0.010 | 0.029 |

PCR data were analysed using Livak method with *GAPDH* as a reference gene.
